# Fluoranthene-Containing Distorted Nanographenes Exhibiting Two-Photon Absorption Response

**DOI:** 10.1021/acs.orglett.5c02904

**Published:** 2025-09-02

**Authors:** John Bergner, José L. Páez, Ermelinda Maçôas, Maria Álvaro-Martins, Jan Borstelmann, Erik Misselwitz, Frank Rominger, Carlos M. Cruz, Milan Kivala, Araceli G. Campaña

**Affiliations:** † Organisch-Chemisches Institut, Universität Heidelberg, Im Neuenheimer Feld 270, 69120 Heidelberg, Germany; ‡ Department of Organic Chemistry, Faculty of Sciences, Unidad de Excelencia de Química (UEQ), University of Granada, Avda. Fuente Nueva s/n, 18071 Granada, Spain; § Centro de Química Estrutural and Institute of Molecular Science, Instituto Superior Técnico, Universidade de Lisboa, Av. Rovisco Pais 1, 1049-001 Lisboa, Portugal

## Abstract

Two distorted nanographenes combining helicenes and a fluoranthene unit within their polycyclic scaffolds were synthesized. Their structural and electronic properties were elucidated by various spectroscopic methods, and the experimental data were corroborated computationally. The optical and electrochemical properties of the nanographenes were evaluated. The compounds exhibit an improved two-photon absorption cross-section compared to their seven-membered ring-containing counterparts reported previously by us. These results provide important insights into how the incorporation of a pentagonal ring affects two-photon absorption properties of nanographenes. Moreover, one of the nanographenes is chiral and was obtained enantiomerically pure after chiral separation, allowing the study of its chiroptical properties both in absorption (electronic circular dichroism) and emission (circularly polarized luminescence).

## Graphical abstract

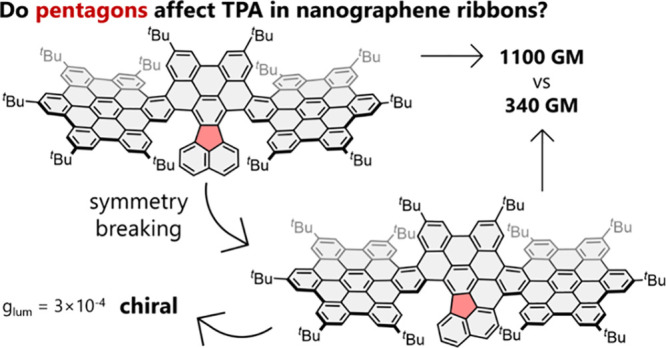

Nanographenes (NGs), nanometer-sized graphene fragments with defined structure, have captured the attention of the scientific community due to the versatility in their structure, magnetic, electronic and optical properties.^1^ The high degree of tunability of NGs is closely linked to their topology, which influences their magnetic and optoelectronic response.^2–4^ Thus, by controlling the shape, edge configuration and/or heteroatom-doping of NGs it becomes possible to finely adjust their response.^5,6^ One of the interesting properties of NGs is their nonlinear absorption, due to their potential applications in microfabrication^7,8^ or volumetric imaging.^9–11^ The simultaneous absorption of two photons is a noteworthy third-order nonlinear optical process observed in polycyclic conjugated hydrocarbons with a highly delocalized π-conjugated structure. The two absorbed photons can be converted into a single higher-energy (shorter wavelength) photon resulting in upconverted emission. Thus, intense two-photon induced fluorescence is commonly observed in carbon and graphene quantum dots.^12–15^ This fact makes NGs good candidates for the development of two-photon absorption- (TPA)-responsive carbon-based materials. It is well-known that π-conjugation is a critical structural requirement to induce a high TPA cross-section (σ_2_), which can be further enhanced if the system exhibits excited state charge transfer, usually associated with the presence of donor and acceptor groups.^16^ Apart from functionalization, symmetry plays a key role. Centrosymmetric molecules are expected to exhibit an enhanced σ_2_ as recently reported by Tan and co-workers in unfunctionalized hexa-branched NGs.^17^ Also, topology plays a key role and, recently, examples of NGs bearing five-membered rings have shown interesting TPA responses.^17,18^ Previous observations in corannulene-thiophene hybrid oligomers suggested an additive effect to some extent.^19^ Additionally, some of us have previously proved that inducing topological modifications in NGs can lead to an increase in the TPA response. Thus, the inclusion of non-hexagonal rings into the structure of NGs leads to an increase in the σ_2_ values of NGs presenting a seven-membered ring over the ones comprising exclusively benzenoid rings (Figure 1).^20^ These observations raise intriguing questions about the effect of the incorporation of five-membered rings on the TPA response of distorted NGs. Therefore, we designed and synthesized two NGs (**1** and **2**, Figure 1) incorporating fluoranthene moiety and comprehensively investigated their structural, optoelectronic and chiroptical properties. We identified an increase of the TPA response upon the incorporation of a pentagonal ring into the structure of NGs.

**Figure 1:**
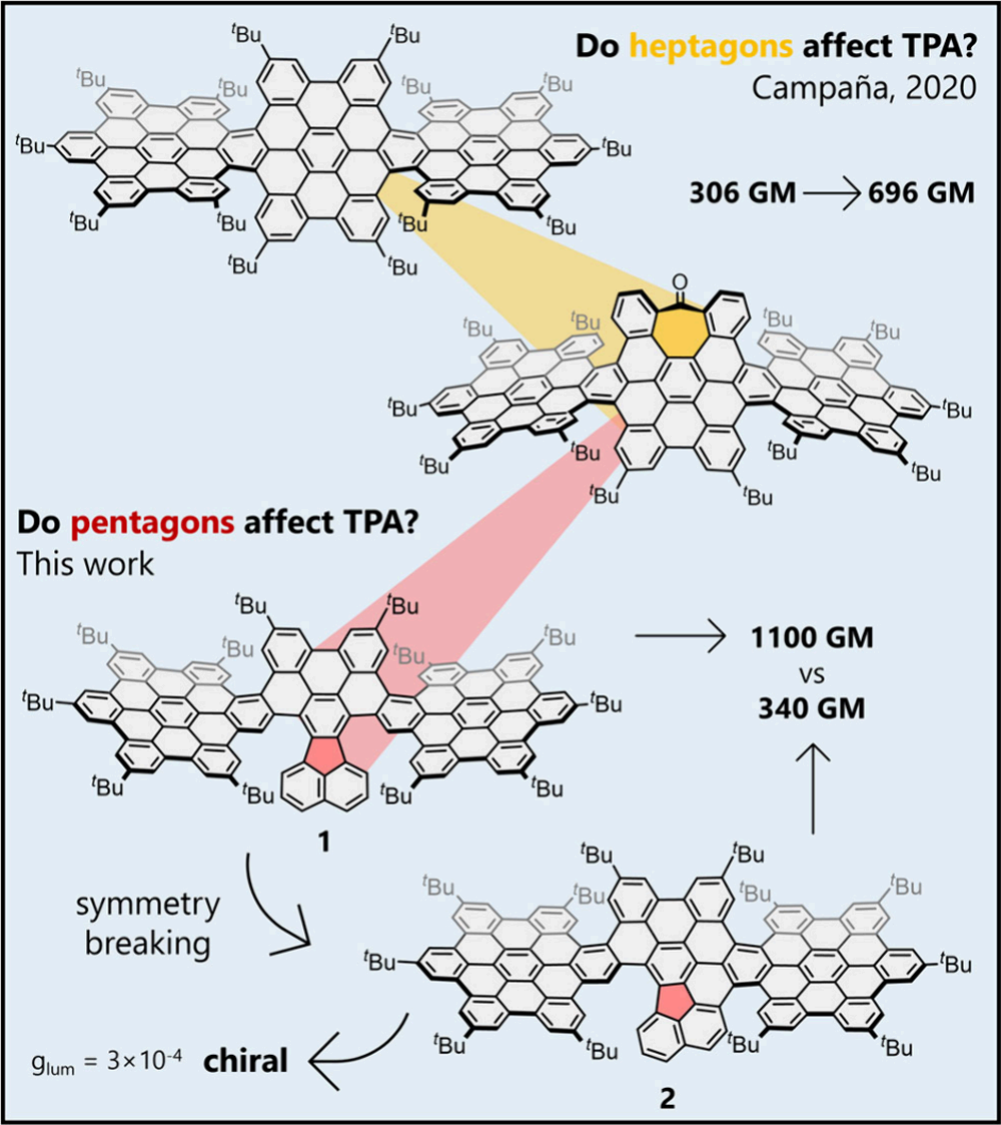
Examples of NGs exhibiting TPA response, their TPA cross-section values, and structures of NGs **1** and **2** reported herein.

The synthesis of compounds **1** and **2** started from cyclopentadienone **3** (Scheme 1), which was prepared from acenaphthene quinone.^21^ Subsequent cycloaddition with bis­(4-*tert*-butylphenyl)­acetylene generated fluoranthene **4**. Upon cyclodehydrogenation of **4** with DDQ and trifluoromethanesulfonic acid, π-expanded fluoranthene **5** was formed in 55% yield. Subsequently, the two-fold Sonogashira coupling of *tert*-butylphenylacetylene to the aromatic framework of **5** generated diethynylated compound **6**, which readily underwent a Diels–Alder cycloaddition with tetracyclone S1 (Supporting Information, Scheme S1) to form oligophenylene **7**. The structure of **7** was confirmed by single-crystal X-ray diffraction, exhibiting negative curvature in the central subunit with the fluoranthene unit standing out of the rest of the structure (Supporting Information, Figure S24–S25). Finally, the twelve-fold cyclodehydrogenation of **7** afforded orange compound **1** in 61% yield. In addition, compound **2**, which contains an additional C–C bond, was isolated in 10% yield as a red solid (Scheme 1).

**Scheme 1:**
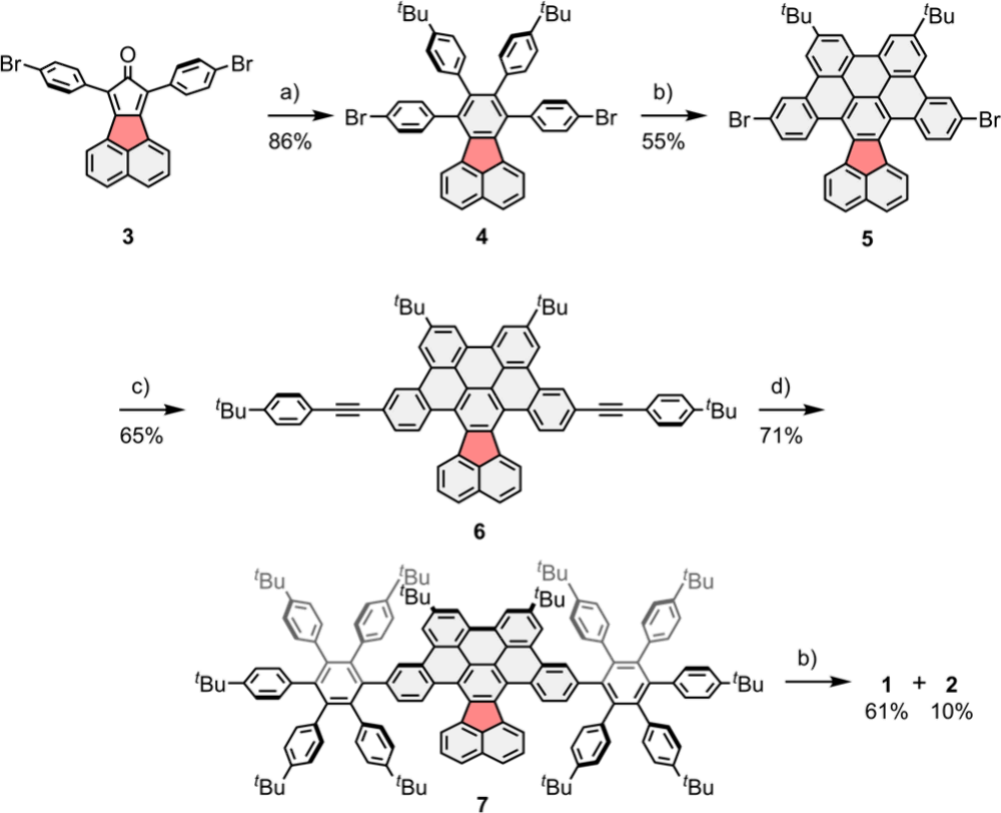
Synthetic Route Towards Compounds **1** and **2** ^a^

a Reagents and conditions: a) bis­(4-*tert*-butylphenyl)­acetylene, 250 °C, 18 h, Ar; b) DDQ, TfOH (1 vol%), CH_2_Cl_2_, 0 °C, 30 min, Ar; c) 4-(*tert*-butylphenyl)­ethynylbenzene, CuI, [PdCl_2_(PPh_3_)_2_], NEt_3_/THF (2:1), 100 °C, 18 h, Ar; d) 2,3,4,5-tetrakis­(4-(*tert*-butyl)­phenyl)­cyclopenta-2,4-dien-1-one (S1), 250 °C, 18 h, Ar.

The structures of **1** and **2** were confirmed by NMR spectroscopy (Supporting Information, Figure S11–S13) and HR-MS (Supporting Information, Figure S18–S19). The measured ^1^H NMR spectrum of **1** could fit within two different symmetric diastereomers: *meso* (*P,M*)-**1** (*meso*-**1**) or the *C*_2_-symmetric axially chiral (*P,P*)/(*M,M*)-**1** (Supporting Information, Figure S11). Computational optimization of the geometries of both diastereomers and analysis of their relative energies (B3LYP/6–31G­(d,p)) suggest *meso*-**1** to be 3.02 kcal mol^–1^ lower in energy than (*P,P*)-**1**. On the other hand, compound **2** exhibits a more complex ^1^H NMR spectrum (Supporting Information, Figure S13) due to its *C*_1_-symmetric structure. The ^1^H NMR spectrum of **2** could fit within either the (*P,M,M*)/(*M,P,P*) or (*M,M,M*)/(*P,P,P*) diastereomeric pairs. DFT optimizations suggest that (*P,M,M*)/(*M,P,P*)-**2** is only 1.68 kcal mol^–1^ lower in energy than their respective diastereomers. However, one should not discard a Curtin-Hammett scenario during the final Scholl reaction that might yield the (*P,P,P*)/(*M,M,M*)-**2** pair.^22^ According to DFT, the [5]­helicene moieties of *meso*-**1** feature a torsion angle (φ) of 24.1°.

In (*M,M,M*)-**2** these helicenes show a φ = 24.9° and the helicene moiety fused to the fluoranthene subunit a φ = 21.2°, since the fused five-membered ring increases the fjord wideness. In *meso*-**1**, the central *seco*-HBC-like unit is twisted with respect to the lateral HBC units by 45.7° and, remarkably, these HBCs are not coplanar, exhibiting a bending angle of 22.3° (Supporting Information, Figure S41). In (*M,M,M*)-**2** the superacene-like structure induces a torsion angle of 75.1° between the edge benzenoid rings (Supporting Information, Figure S43).

Aromaticity indexes were analyzed through the harmonic oscillator model of aromaticity (HOMA), the nucleus independent chemical shift (NICS) and the anisotropy of the induced current density (ACID) (Supporting Information, sections 15 and 16). The HOMA and NICS(0)_iso_ analyses show the expected values for an armchair-edged NG with well-localized Clar sextets, also confirmed in the ACID analysis (Figure 2). The five-membered ring shows antiaromatic character in *meso*-**1** according to the HOMA and NICS values; however, no paratropic ring current was observed in the ACID plot, where the diatropic currents of the naphthalene subunit of the fluoranthene moiety are clearly independent to those of the rest of the NG. Compound **2** exhibits the same localized Clar sextets, however, the five-membered ring shows a paratropic ring current (Figure 2). This is in accordance with the calculated NICS value of the pentagonal ring, which is higher in **2** than in *meso*-**1** (7.84 vs 5.58).

**Figure 2:**
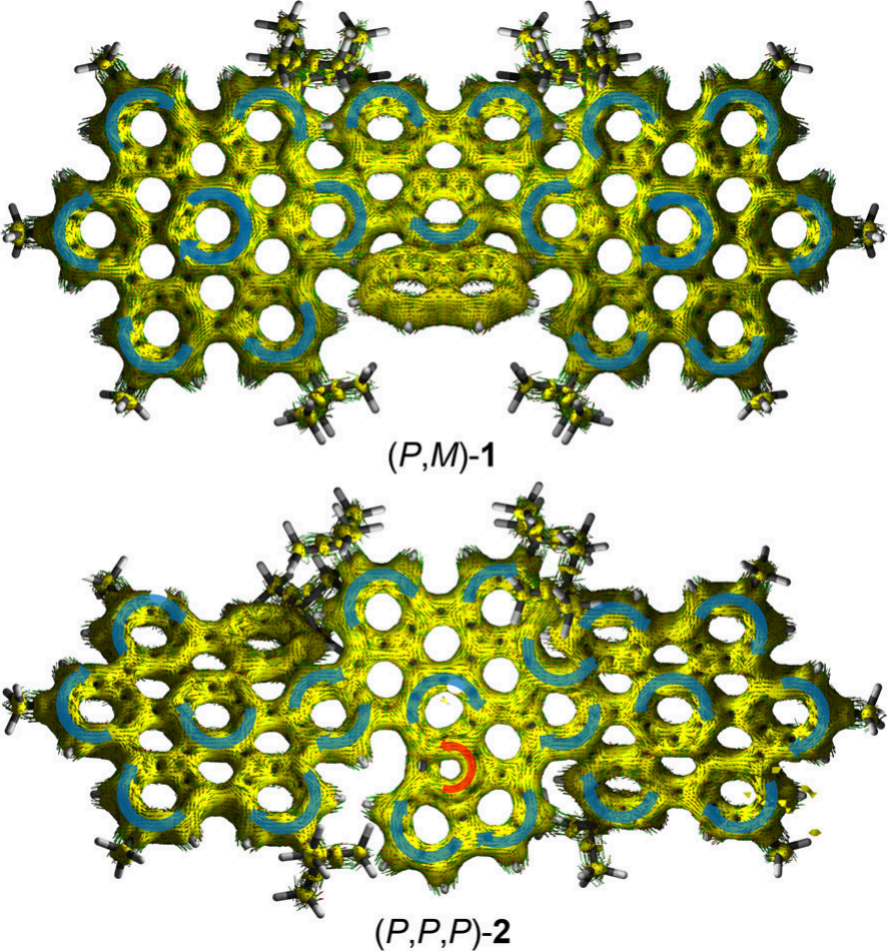
ACID plots (CSGT/B3LYP/6–31G­(d,p), isovalue: 0.05) of compound (*P*,*M*)-**1** and (*P*,*P*,*P*)-**2**, diatropic (blue) and paratropic (red) ring currents are depicted for better visibility. Magnetic field oriented toward the spectator.

The photophysical properties of **1** and **2** were evaluated in CH_2_Cl_2_ at room temperature. Compound **1** exhibits a UV–vis absorption up to 600 nm with maxima at 372, 417, and 446 nm, with ε = 1.79 × 10^5^, 1.73 × 10^5^ and 1.72 × 10^5^ M^–1^ cm^–1^, respectively (Figure 3). TD-DFT calculations (CAM-B3LYP/6–31G­(d,p)/PCM, correction = – 0.4 eV) predicted two main transitions at 427 (*f* = 2.4574) and 384 nm (*f* = 1.4888) for *meso*-**1**, in accordance with the experimental spectrum (Supporting Information, Figure S46). Compound **2** shows a very different absorption profile with two red-shifted maxima centered at 530 and 572 nm, with ε = 0.44 × 10^5^, 0.60 × 10^5^ M^–1^ cm^–1^, respectively. Interestingly, compound **2** exhibits a similar absorption profile to previously reported supertwistacenes.^20,22^ Calculated electronic transitions for (*M*,*M*,*M*)-**2** predict a red-shifted UV–vis absorption profile, with a transition at 480 nm (*f* = 1.1793) dominated by a HOMO→LUMO character (Supporting Information, Figure S49). Remarkably, HOMO and LUMO are located over the extra helicene moiety in (*M*,*M*,*M*)-**2**, which supports the red-shifted absorption, as previously reported for a NIR emitting NG.^23,24^ Fluorescence spectra of **1** and **2** were recorded in CH_2_Cl_2_ upon excitation at 370 nm at room temperature. Compound **1** exhibits an emission band centered at 532 nm, with a clear vibronic progression, and a fluorescence quantum yield (ϕ_*F*_) of 0.15 with a fluorescence lifetime (τ) of 7.98 ns. In contrast, compound **2** shows an emission centered at 601 nm (ϕ_*F*_ = 0.42, τ = 3.95 ns) with a shoulder around 650 nm. The less marked vibronic progression might be due to the lower symmetry. The increased ϕ_*F*_ and reduced τ is indicative of a faster radiative decay rate of **2** (*k*_r_ = 1.1 × 10^8^ s^–1^) when compared to **1** (*k*_r_ = 0.19 × 10^8^ s^–1^).

**Figure 3:**
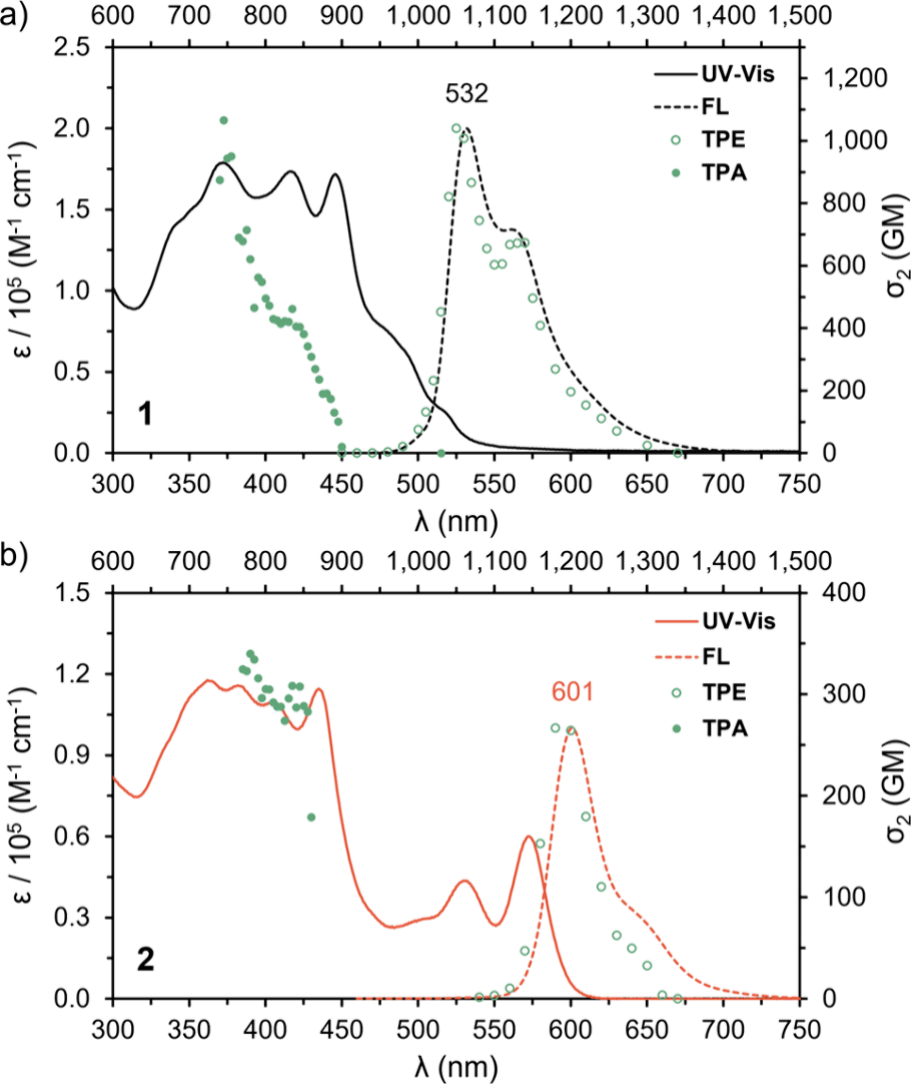
a) UV–vis absorption (one photon absorption, OPA, black line) and emission (one photon emission, OPE, black dashed line, λ_exc_ = 370 nm) spectra of compound **1** measured at 10^–5^ M in CH_2_Cl_2_. Two-photon absorption (TPA, green dots) and two-photon emission (TPE, green circles) spectra of compound **1** measured at 10^–6^ M in CH_2_Cl_2_. b) UV–vis absorption (OPA, orange line) and emission (OPE, orange dashed line, λ_exc_ = 370 nm) spectra of compound **2** measured at 10^–5^ M in CH_2_Cl_2_. Two-photon absorption (TPA, green dots) and two-photon emission (TPE, green circles) spectra of compound **1** measured at 10^–6^ M in CH_2_Cl_2_.

The TPA spectra of **1** and **2** were recorded in CH_2_Cl_2_ using the two-photon induced fluorescence method. Compound **1** exhibits a larger σ_2_ value of 1100 GM at 750 nm (Figure 3a) when compared with the 340 GM of compound **2** at 780 nm (Figure 3b). Based on the larger extent of conjugation of compound **2**, the opposite would be expected. The larger σ_2_ values of compound **1** might originate from a vibronic contribution to the TPA into higher energy state.^25^ Vibronic coupling is typically evoked to explain cooperative enhancements in the TPA cross-section of branched structures that are not well conjugated.^25^ Indeed, the vibronic progression in the emission spectra is more pronounced in compound **1**.

To evaluate the effect of the fluoranthene on the TPA response, we can compare the σ_2_ value of compounds **1** and **2** with those of other linear NGs.^20^ The σ_2_ value of compound **1** is higher than any of the three linear HBC-based NGs reported earlier (306–696 GM). Compound **1** has a lower number of conjugated carbon atoms and exhibits no dipolar carbonyl group compared to the tropone-containing NG (Figure 1), features that would enhance the TPA response.^20^ Thus, the higher TPA response suggests also a positive effect of the inclusion of a five-membered ring into the delocalized π-system in these particular compounds. Conversely, the σ_2_ value of compound **2** is similar to that obtained for the fully hexagonal, *meso* supertwistacene comprising three HBC units (Figure 1).^20^ Based on the similarities between their UV–vis absorption spectra, both compounds appear to have similar conjugation length, which in the absence of any push–pull effect in these molecules, is one of the main factors determining the magnitude of the TPA cross-section. The design guidelines for enhancing the TPA response in 2D-materials seem to be rather elusive. Comprehensive investigations on well-defined structures are essential to identify the key structural factors that govern TPA efficiency.

Remarkably, the high ϕ_*F*_ of **2** compensates its lower σ_2_ resulting in a TPA brightness (*B*_*TPA*_ = σ_2_ × ϕ_*F*_) of 143 GM, similar to that of **1** with 165 GM. In both compounds, the obtained emission spectra after TPA excitation matched the ones obtained after single-photon excitation, suggesting a similar excited state for TPE and OPE.

The enantiomers of compound **2** were obtained after separation on a chiral stationary phase HPLC (CSP-HPLC) with recycling system. The chiroptical properties, electronic circular dichroism (ECD) and circularly polarized luminescence (CPL), were evaluated for both enantiomers in CH_2_Cl_2_ solutions. The obtained ECD spectra are mirror-imaged (Figure 4). Intense Cotton effects were recorded at 260 nm (|Δε| = 400 M^–1^ cm^–1^) and 371 nm (|Δε| = 332 M^–1^ cm^–1^). The ECD spectra are characterized by a weak band centered at 580 nm (15 M^–1^ cm^–1^), matching the lowest energy band observed in the UV–vis spectrum. The determination of the absolute configuration was addressed using TD-DFT calculations. Thus, the ECD spectrum with negative Δε at the lowest energy Cotton effect was assigned to (*M,M,M*)-**2** (Supporting Information, Figure S50). Additionally, CPL spectra of both enantiomers were recorded after irradiation with 370 nm light in the 550–700 nm range. The obtained luminescence dissymmetry factor (|*g*_*lum*_|) was evaluated as 3 × 10^–4^ (Supporting Information, Figure S38), with a CPL brightness (*B*_*CPL*_) of 7.22 M^–1^ cm^–1^. These values are comparable to other chiral NGs reported previously.^26–31^

**Figure 4:**
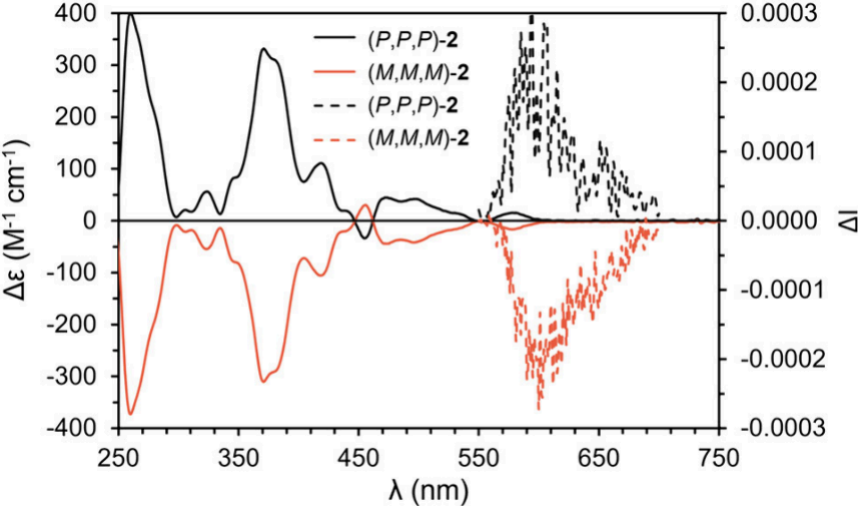
Experimental ECD spectra of (*P*,*P*,*P*)-**2** (black) and (*M*,*M*,*M*)-**2** (orange) measured in CH_2_Cl_2_ and experimental CPL spectra (λ_exc_ = 370 nm) of (*P*,*P*,*P*)-**2** (black, dashed) and (*M*,*M*,*M*)-**2** (orange, dashed) measured in CH_2_Cl_2_.

Redox properties of **1** were evaluated by cyclic voltammetry (CV), differential pulse voltammetry (DPV) and square wave voltammetry (SWV) in THF with ferrocene/ferrocenium (Fc/Fc^+^) redox couple as a reference (Supporting Information, Figure S35). Compound **1** exhibits reversible reductions at – 2.33, – 2.15 and – 1.96 V and reversible oxidations at +0.70 and +0.79 V. This translates into an electrochemical gap of 2.66 eV, which is in accordance with DFT calculations (2.86 eV, B3LYP/6–31g­(d,p)) and the optical gap (2.43 eV, calculated from the crossing point between absorption and emission). Unfortunately, the low availability of **2** prevented its electrochemical characterization. However, its optical gap of 2.14 eV was estimated from the UV–vis absorption data, which is in good agreement with the DFT calculated value of 2.50 eV.

In conclusion, we report the synthesis of two nanographenes with an overall twisted geometry resulting from the careful combination of helicene moieties and a five-membered ring in their sp^2^ carbon polycyclic backbone. Comprehensive characterization by various methods confirmed the structural and electronic differences resulting from the altered connectivity of the two nanographenes. A small structural change (an additional C–C bond) has a pronounced impact on both the linear and nonlinear photophysical properties that were evaluated spectroscopically. Nanographene **2** shows a red-shifted absorption due to its π-extended structure and a lower two-photon absorption as compared to nanographene **1**. Nevertheless, its increased emission quantum yield leverages the two-photon brightness to similar values as those obtained for **1**. The high two-photon absorption cross-section evaluated for **1** compared with structurally related carbon-based nanographenes suggests a beneficial effect of the incorporation of a five-membered ring into nanographenes with a linear overall geometry. These observations open a new path for tuning the properties of nanographenes, paving the way for their application as two-photon absorption molecular materials. They also provide deeper insights into their structure–property relationships.

## Supplementary Material



## Data Availability

The data underlying this study are available in the published article and its Supporting Information.

## Notes

The data underlying this study are available in the published article and its Supporting Information. The Supporting Information is available free of charge on the ACS Publications Web site. Experimental details; characterization data; crystallographic data of compounds **4**, **6** and **7**; photophysical, electrochemical and chiroptical data; computational procedures; and copies of NMR and HRMS spectra (PDF)
