# Supplementary material for: Fluoranthene-Containing Distorted Nanographenes Exhibiting Two-Photon Absorption Response
Source: Org Lett. 2025 Sep 2;27(36):9933–8. doi: 10.1021/acs.orglett.5c02904 (PMC12442070; doi:10.1021/acs.orglett.5c02904)
Supplement: Supplementary file 1 [file ol5c02904_si_001.pdf]

# Supporting Information

# Fluoranthene-containing distorted nanographenes exhibiting two-photon absorption response

John Bergner[a], José L. Páez[b], Ermelinda Maçôas[c], Maria Álvaro-Martins[c], Jan Borstelmann[a], Erik Misselwitz[a], Frank Rominger[a], Carlos M. Cruz[b]*, Milan Kivala[a]* and Araceli G. Campaña[b]*

[a] Organisch-Chemisches Institut, Universität Heidelberg, Im Neuenheimer Feld 270, 69120 Heidelberg (Germany).
[b] Departament of Organic Chemistry, Faculty of Sciences, Unidad de Excelencia de Química (UEQ), University of Granada, Avda. Fuente Nueva s/n, 18071 Granada (Spain).
[c] Centro de Química Estrutural and Institute of Molecular Sciences, Instituto Superior Técnico, Universidade de Lisboa, Av. Rovisco Pais 1, 1049-001 Lisboa (Portugal).

## Table of Contents


| | | |
|---|---|---|
| 1. | General experimental methods | S3 |
| 2. | Synthesis | S6 |
| 3. | NMR data | S13 |
| 4. | HR-MS data | S19 |
| 5. | Crystallographic data | S22 |
| 6. | UV-vis absorption and fluorescence data | S28 |
| 7. | Quantum yield determination and fluorescence lifetimes | S31 |
| 8. | Two-photon absorption | S33 |
| 9. | Electrochemical data | S34 |
| 10. | Chiral stationary phase high performance liquid chromatography | S35 |
| 11. | Absorbance and luminescence dissymmetry factors | S36 |
| 12. | Geometrical aspects | S37 |
| 13. | Calculated frontier molecular orbitals | S40 |
| 14. | Theoretical UV-vis absorption and ECD data | S41 |
| 15. | Harmonic oscillator model of aromaticity (HOMA) and nucleus independent chemical shift (NICS) | S47 |
| 16. | Anisotropy of the induced current density (ACID) | S50 |
| 17. | Cartesian coordinates | S51 |
| 18. | References | S71 |

# 1. General Experimental Methods

## 1.1. General Reaction Conditions

All solvents and reagents were purchased at reagent grade from commercial suppliers (Merck/Sigma-Aldrich, TCI, Thermo Fisher Scientific, Acros Organics, Honeywell, BLD Pharmatech) and used without additional purification. All reactions were performed in sealed Biotage microwave reaction vials (10−20 mL, or 2.0−5.0 mL in combination with aluminum caps and septa). Thin layer chromatography was monitored on ALUGRAM aluminum plates from Macherey-Nagel, coated with 0.20 mm $SiO_2$, by irradiation with UV-light (λ= 365 and 254 nm). Flash column chromatography was carried out with SiO2 from Macherey-Nagel (technical grade 60 M, pore size 60 Å, 40−63 µm particle size).

## 1.2. Instruments Used

**Nuclear Magnetic Resonance Spectra** were recorded at room temperature (295 K) on a Bruker Avance III 300, 400, 500, 600 or 700 at the Institute of Organic Chemistry (Heidelberg University). Proton broad band decoupling was applied for $^{13}C$ measurements. Deuterated solvents were used as purchased from Merck/Sigma-Aldrich. Chemical shifts (reported in parts per million ppm) were referenced[S1] to $\delta_H$ = 7.26 ppm ($CDCl_3$) and 5.32 ppm ($CD_2Cl_2$) for $^1H$ and $\delta_C$ = 77.16 ppm ($CDCl_3$) and 53.84 ppm ($CD_2Cl_2$) for $^{13}C$ and interpreted with MestReNova Version 14.1.2-25024. Apparent signal multiplicity is reported as s (singlet), d (doublet), dd (doublet of doublets), t (triplet), or m (multiplet).

**Absorption and Emission Spectra.** UV/Vis spectra were recorded on an Agilent Cary 60 UV/Vis spectrophotometer or a JASCO V-540 spectrophotometer and measured in $CH_2Cl_2$ in the wavelength region of 230 to 800 nm under ambient conditions (rt: room temperature). The abbreviation br. (broad) sh. (shoulder) refers to saddle points or shoulders in the absorption spectrum. The photoluminescence spectra were collected on a Horiba Jobin Yvon Fluorolog 3-22 spectrofluorimeter or a JASCO FP-8500 Fluorescence Spectrometer with a JASCO ILF-835 (100 mm) integrating sphere. The data obtained was interpreted with Spectra Manager from JASCO.

**Single Photon Timing.** The emission lifetime was measured by the Single-Photon Timing technique in a home-built setup using a linear excitation source operating at 300 nm with a 4 MHz repetition rate (second harmonic of a Coherent Radiation Dye laser 700 series, 560-610 nm, 130 mW, 5 ps, 4 MHz), an Hamamatsu R2809U-01 MCP-PMT (290−700 nm) as the detector and an SPC-160 photon counting board from Becker & Hickl GmbH. The emission of compounds **1** (525 nm) and **2** (600 nm) was collected at the magic angle. The instrument response functions (IRF) for deconvolution were generated by scattering dispersions of colloidal silica in water. The solution was kept under gentle stirring during the data collection. Blank decays were acquired to ensure that dark photon counts were negligible. Decay curves were stored in 1024 channels with either 24.4 (compound **2**) or 48.8 ps (compound **1**) per channel and an accumulation of 10k counts in the peak channel. The fluorescence decays were analyzed by a non-linear least-squares reconvolution method using the TRFA DP software by SSTC (Scientific Software Technologies Center, Belarusian State University, Minsk, Belarus).

**Two-Photon Absorption.** The two-photon absorption spectrum was measured by two-photon excited fluorescence using Coumarin 153 in DMSO and Rhodamine 6G in methanol as a standard to account for collection efficiency and pulse characteristics.[S2] A modified setup that follows closely the one described by Xu and Webb was used. [S3] To select a narrow bandwidth of emission wavelengths an H20Vis Jobin Yvon monochromator was placed at the entrance of a PMC-100-4 photomultiplier tube (Becker and Hickl GmbH). The integrated intensity over the entire emission band was extrapolated using the emission spectra corrected by the detector sensitivity. The excitation source was a Ti:Sapphire laser (Tsunami BB, Spectra-Physics, 710-990 nm, 1.7 W, 100 fs, 82 MHz). In general, solutions of ca. 10 µM concentration in dichloromethane were used in the characterization of the non-linear properties.

The two-photon absorption cross-section was calculated from eq. 1:

$$\sigma_2 = \left(\frac{F_2}{\phi C n}\right)_s \left(\frac{\phi C n \sigma_2}{F_2}\right)_{ref} \qquad \text{eq. 1}$$

where $F_2$ stands for two-photon induced fluorescence intensity, ϕ is the one-photon excited fluorescence quantum yield, *n* refers to the refractive index in solution, *C* is the concentration and *s* and *ref* are relative to the unknown sample and the TPA standard, respectively. The emission intensity dependence of the excitation power was checked to be quadratic at the excitation maximum for all the compounds.

**Chiral Stationary Phase High-Performance Liquid Chromatography.** High performance liquid chromatography (HPLC) was performed on a setup from Shimadzu consisting of a LC20 AP preparative pump, a DGU 405 degassing unit, a CTO 40C column oven, an SPD M40 diode array detector, an FCV 20AH2 valve unit, an FRC 10A fraction collector and an CBM 20A communication bus module. On the analytical scale, a Daicel CHIRALPAK IA-3 (250 × 4.6 mm) column equipped with a column protection system and a Daicel precolumn (10 mm × 4.0 mm) was used. On the semi-preparative scale, a Daicel CHIRALPAK IA-5 (250 × 20 mm) column equipped with a column protection system and a Daicel 5 µm (20 mm × 10 mm) precolumn was employed. The column temperature was set at 40 °C and the flow rate was constant at 24 mL $min^{-1}$. As the mobile phase a mixture of n-heptane and isopropanol (85:15 v/v) was selected.

**Electronic Circular Dichroism.** Electronic Circular Dichroism (ECD) spectra of the enantiopure samples were recorded on a JASCO J-1700 spectrophotometer and measured at concentrations of $10^{-5}$ M in $CH_2Cl_2$ (HPLC grade) in the wavelength region 230 to 800 nm under ambient conditions at rt.

**CPL.** The CPL measurements were recorded in an Olis DSM172 spectrophotometer equipped with a 370 nm LED light source, a Hinds Instruments Photoelastic modulator (PEM90) and a Hamamatsu H10682-01 photon counting as detector. The spectra were recorded at ca. $1\times10^{-5}$ M concentration in HPLC grade DCM at 20.0 °C. The measurement range was selected between 550 and 700 nm, where 300 points were measured with an integration time of 1.0 s. The CPL spectra were obtained after averaging a total of 100 scans (for each enantiomer). The obtained spectra were not processed, and no filter or smoothing was applied.

**X-ray Crystallography.** Single crystals were obtained by slow gas phase diffusion under the given conditions. The Bruker APEX-II Quazar diffractometer (radiation MoKα, λ = 0.71073 Å) with a CCD area detector and the STOE Stadivari instrument (radiation CuKα, λ=1.54178 Å) with a Pilatus CCD area detector (0.5° ω-scans) were used for data collection by the X-ray crystallography department of the Institute of Organic Chemistry (Heidelberg University). Structures were solved with the ShelXT[S4] structure solution program and refined against F2 with a full-matrix least- squares algorithm with ShelXL.[S5] Hydrogen atoms were treated with riding models. Graphic visualization and measurement of torsion angles were done with Mercury 2020.1.[S6]

**Electrochemistry.** A BASi Cell Stand instrument with a glassy carbon disk working electrode (3.0 mm diameter), an Ag/AgCl (3 M NaCl) quasi-reference electrode, and a platinum wire auxiliary electrode were used to record cyclic voltammograms, differential pulse voltammograms and square wave voltammograms. Before each measurement, a 0.1 M electrolyte solution of $n\text{-}Bu_4NPF_6$ in anhydrous $CH_2Cl_2$ was degassed with nitrogen for 20 min. The respective compounds were measured either at a scan rate of 50 mV $s^{-1}$ or 149 mV $s^{-1}$, followed by the addition of ferrocene as the internal standard and re-measurement

**Infrared Spectroscopy.** A JASCO FT/IR-6000 FT-IR spectrometer was operated in ATR mode to record infrared spectra. The respective transmission spectra are baseline corrected, depicted in $cm^{-1}$ and labeled according to the following abbreviations: s (strong), m (medium), w (weak), and br (broad).

**Melting Point.** The melting point was determined on a Büchi M-560 melting point apparatus in open capillaries. Decomp. refers to decomposition.

**Mass Analysis.** Mass spectra were received from the facility of Heidelberg University and recorded on a JEOL AccuTOF GCx (electron ionization (EI)), a Bruker timsTOFfleX (matrix assisted laser desorption ionization (MALDI)) or a Bruker ApexQe FT-ICR (electrospray ionization (ESI)) instrument. Simulated MS spectra were generated using mMass software.[S7]

**Computational Details.** The Gaussian 16[S8] (revision C.02) software package was used to investigate the structural and electronic properties of compounds **1** and **2**. The geometry of **1** and **2** was obtained from DFT optimizations using the Becke three-parameters exchange function in combination with the Lee-Yang-Parr correlation functional (B3LYP).[S9] The basis set 6-31G(d,p) was used for the optimization. Solvent effects were included via the Polarizable Continuum Model (PCM) as implemented in Gaussian 16 with the dielectric constant of dichloromethane.[S10] Optimized geometries were confirmed to be stationary points by analysis of their vibrational frequencies. The 50 lowest energetic transitions were calculated by TD-DFT as implemented in Gaussian 16, using the Coulomb-attenuated B3LYP (CAM-B3LYP) functional, selecting the 6-31G(d,p) basis set and considering solvent effects. Electronic transitions were corrected by –0.4 eV to better fit experimental results. Nucleus independent chemical shifts (NICS) were calculated using the Gauge-Independent atomic orbital (GIAO) approach,[S11] as implemented in Gaussian 16 at the GIAO-B3LYP/6-31G(d,p) level of theory. Values were obtained from the centroid of each ring. Ring-current analysis was accomplished by using the continuous set of the gauge transformations (CSGT) method at the CSGT-B3LYP/6-31G(d,p) level of theory and visualized using the ACID program package of the Herges group.[S12] Aromaticity index was also calculated using the harmonic oscillator model of aromaticity (HOMA).

## 2. Synthesis

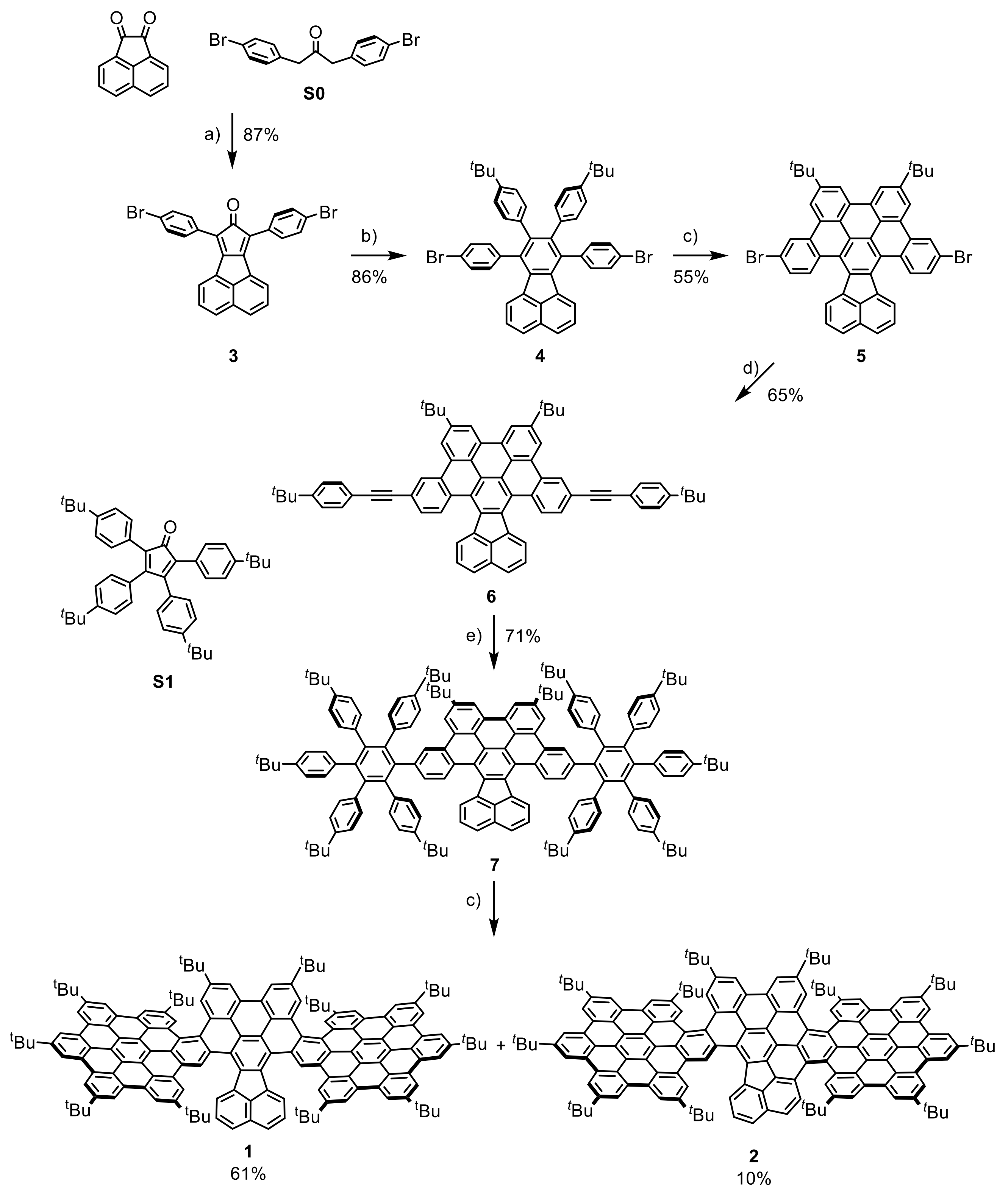


**Scheme S1.** Synthetic route towards **1** and **2**. Reagents and conditions: a) KOH, EtOH, 90 °C, 20 min; b) bis(4-*tert*-butylphenyl)acetylene, 250 °C, 18 h, Ar; c) DDQ, TfOH (1 vol%), $CH_2Cl_2$, 0 °C, 30 min, $N_2$; d) 4-(*tert*-butylphenyl)ethynylbenzene, CuI, $[PdCl_2(PPh_3)_2]$, $NEt_3$/THF (2:1), 100 °C, 18 h, Ar; e) **S1**, 250 °C, 18 h, Ar.

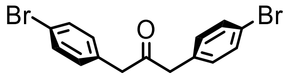

**S0**

**1,3-Bis(4-bromophenyl)propan-2-one** (**S0**). A 250 mL flask was charged with 4-DMAP (1.70 g, 14.0 mmol), DCC (10.6 g, 51.2 mmol) and $CH_2Cl_2$ (25.0 mL). Subsequently, (4-bromophenyl)acetic acid (10.0 g, 46.5 mmol) dissolved in $CH_2Cl_2$ (25.0 mL) was added dropwise over a time period of 20 min. The reaction mixture was stirred for 24 h at rt. The formed precipitate was filtered off and the crude product obtained after evaporation of the solvent was subjected to column chromatography ($SiO_2$, PE/EtOAc 10:1). **S0** (6.15 g, 16.7 mmol, 72%) was obtained as colorless crystals after evaporation of the solvent.

M.p.: 116–118 °C (lit. 116–118 °C).[1]

$R_f$ = 0.31 ($SiO_2$, PE/EtOAc 9:1).

$^1$H NMR (400 MHz, $CD_2Cl_2$): $\delta$ = 7.46 (dt, $J$ = 8.4, 1.9 Hz, 4H), 7.04 (dt, $J$ = 8.5, 1.9 Hz, 4H), 3.71 (s, 4H) ppm.

$^{13}$C NMR (101 MHz, $CD_2Cl_2$): $\delta$ = 204.5, 133.5, 132.1, 131.8, 121.4, 48.8 ppm.

IR (FT-ATR): $\tilde{\nu}$ = 3324 (w), 3094 (w), 3070 (w), 3039 (w), 2926 (m), 2886 (w), 2849 (w), 2360 (w), 2335 (w), 1715 (s), 1625 (m), 1575 (m), 1487 (s), 1415 (m), 1397 (m), 1340 (s), 1319 (m), 1306 (m), 1243 (w), 1180 (w), 1102 (w), 1070 (m), 1056 (s), 1011 (s), 957 (w), 942 (w), 892 (w), 839 (m), 823 (s), 800 (s), 791 (s), 768 (s), 730 (s), 668 (m) cm$^{-1}$.

HRMS (EI) m/z: [M$^+$] Calcd for $C_{15}H_{12}{}^{81}Br_2O$ 369.9209; Found 369.9207.

The obtained data are in agreement with those reported in literature.[S13, S14]

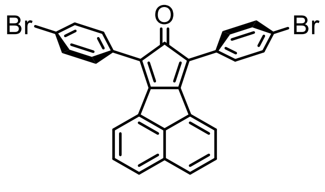

**3**

**7,9-Bis(4-bromophenyl)-8*H*-cyclopenta[*a*]acenaphthylen-8-one** (**3**). According to a modified literature procedure, a microwave tube was charged with acenaphthoquinone (850 mg, 4.67 mmol), 1,3-bis(4-bromophenyl)propan-2-one (**S0**) (1.89 g, 5.13 mmol), and EtOH (100 mL). The reaction mixture was ultra-sonicated to obtain a fine suspension and heated to 90 °C in an oil bath. KOH (262 mg, 4.67 mmol) dissolved in EtOH (5.00 mL) was added dropwise to the hot reaction mixture

under continuous stirring. The reaction mixture was heated for additional 20 min and then cooled to rt. Filtration of the crude reaction mixture obtained **3** (2.08 g, 4.05 mmol, 87%) as bluish insoluble solid.

M.p.: >400 °C.

$R_f$ = Insolubility of the compound in common solvents prevented to obtain a meaningful value.

NMR: Insolubility of the compound in common deuterated solvents prevented to obtain a meaningful spectrum.

IR (FT-ATR): $\tilde{\nu}$ = 3070 (w), 3038 (w), 2958 (w), 2176 (w), 2153 (w), 1949 (w), 1901 (w), 1742 (w), 1702 (s), 1650 (w), 1621 (w), 1585 (m), 1487 (s), 1444 (w), 1410 (w), 1394 (s), 1351 (w), 1304 (m), 1293 (s), 1224 (w), 1204 (w), 1179 (w), 1121 (s), 1070 (s), 1009 (s), 983 (w), 956 (m), 918 (w), 871 (w), 822 (s), 777 (s), 761 (s), 727 (m), 701 (m), 689 (m), 628 (m) $cm^{-1}$.

HRMS (LDI) m/z: [$M^+$] Calcd for $C_{27}H_{14}{}^{79}Br_2O$ 511.9400; Found 511.9405.

The obtained data are in agreement with those reported in literature.[S15,S16]

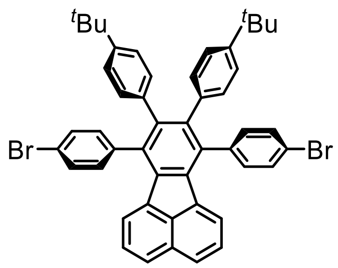

**4**

**7,10-Bis(4-bromophenyl)-8,9-bis(4-*tert*-butylphenyl)fluoranthene** (**4**). A microwave tube was charged with **3** (750 mg, 1.46 µmol) and bis(4-*tert*-butylphenyl)acetylene (466 mg, 1.60 mmol) under argon atmosphere. The sealed tube was maintained at 250 °C in a sand bath for 18 h. After cooling to rt, the crude material was subjected to column chromatography ($SiO_2$, PE/$CH_2Cl_2$ 10:1) to afford the desired compound **4** (973 mg, 1.25 µmol, 86%) as yellow solid after evaporation of the solvent.

M.p.: 280–282 °C.

$R_f$ = 0.63 ($SiO_2$, PE/$CH_2Cl_2$ 3:1).

$^1$H NMR (600 MHz, $CD_2Cl_2$): $\delta$ = 7.76 (d, $J$ = 8.1 Hz, 2H), 7.48 (dt, $J$ = 8.4, 1.9 Hz, 4H), 7.35 (dd, $J$ = 8.1, 7.3 Hz, 2H), 7.22 (dt, $J$ = 8.4, 1.9 Hz, 4H), 6.92 (dt, $J$ = 8.4, 1.9 Hz, 4H), 6.77 (dt, $J$ = 8.4, 1.9 Hz, 4H), 6.65 (d, $J$ = 7.1 Hz, 2H), 1.13 (s, 18H) ppm.

$^{13}$C NMR (151 MHz, $CD_2Cl_2$): $\delta$ = 148.8, 141.4, 139.4, 136.9, 136.6, 136.49, 136.45, 133.4, 132.3, 131.7, 131.2, 130.1, 128.1, 127.1, 123.9, 123.5, 121.4, 34.4, 31.3 ppm.

IR (FT-ATR): $\tilde{\nu}$ = 3028 (w), 2951 (s), 2900 (m), 2863 (w), 2362 (m), 2341 (w), 1902 (w), 1610 (w), 1589 (w), 1511 (w), 1492 (s), 1463 (m), 1423 (s), 1390 (s), 1362 (s), 1266 (m), 1227 (w), 1200 (w), 1178 (w), 1111 (m), 1092 (m), 1068 (s), 1043 (w), 1011 (s), 923 (w), 854 (w), 821 (s), 776 (s), 729 (m), 715 (m), 676 (m), 631 (m), 612 (w) $cm^{-1}$.

UV/vis ($CH_2Cl_2$, rt): $\lambda_{max}$ (ε) 292 (21700), 325 (5900), 369 (6600) nm ($M^{-1}$ $cm^{-1}$).

Fluorescence ($CH_2Cl_2$, rt): $\lambda_{ex}$ = 300 nm, $\lambda_{em}$ = 466 nm, $\phi_F$: 0.22.

HRMS (MALDI, DCTB) m/z: [$M^+$] Calcd for $C_{48}H_{40}{}^{79}Br_2$ 774.1491; Found 774.1493.

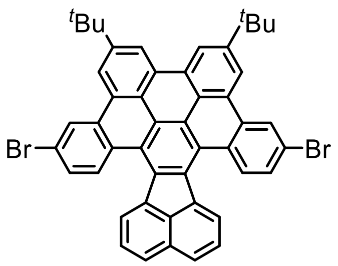

**5**

**9,18-Dibromo-12,15-di-*tert*-butyldibenzo[*fg,ij*]fluorantheno[7,8,9,10-*rst*]pentaphene** (**5**).
Compound **4** (250 mg, 322 µmol) and DDQ (241 mg, 1.06 mmol) were dissolved in dry $CH_2Cl_2$ (500 mL), then the solution was cooled to 0 °C and deoxygenated with nitrogen for 10 min. To the cooled solution was added TfOH (5.00 mL, 1 v%) dropwise over 5 min under continuous stirring. After additional 30 min of stirring at this temperature the reaction mixture was quenched via dropwise addition of sat. aq. $Na_2CO_3$ (50.0 mL). The reaction was extracted with $CH_2Cl_2$ (3×200 mL), dried over $Na_2SO_4$, and filtered. The solvent was removed to give the crude material which was purified by column chromatography ($SiO_2$, PE/$CH_2Cl_2$ 10:1). Compound **5** (137 mg, 178 µmol, 55%) was obtained as orange solid upon evaporation of the solvent under reduced pressure.

M.p.: >400 °C.

$R_f$ = 0.54 ($SiO_2$, PE/$CH_2Cl_2$ 3:1).

$^1H$ NMR (600 MHz, $CD_2Cl_2$): $\delta$ = 9.06 (s, 2H), 8.91 (d, $J$ = 8.5 Hz, 2H), 8.75 (dd, $J$ = 8.7, 1.6 Hz, 4H), 8.39 (d, $J$ = 7.2 Hz, 2H), 7.75 (d, $J$ = 7.9 Hz, 2H), 7.40–7.35 (m, 4H), 1.74 (s, 18H) ppm.

$^{13}C$ NMR (151 MHz, $CD_2Cl_2$): $\delta$ = 149.9, 137.5, 133.6, 133.4, 132.3, 131.2, 130.5, 129.5, 128.78, 128.75, 128.5, 128.0, 127.5, 126.6, 125.4, 123.6, 123.5, 122.9, 122.4, 120.1, 119.3, 36.0, 32.0 ppm.

IR (FT-ATR): $\tilde{\nu}$ = 2952 (m), 2923 (m), 2851 (w), 1593 (w), 1577 (w), 1463 (w), 1449 (w), 1423 (m), 1388 (m), 1362 (m), 1335 (w), 1258 (m), 1242 (m), 1199 (w), 1173 (w), 1090 (m), 1069 (w), 1051 (w), 1011 (m), 903 (s), 868 (s), 847 (w), 819 (s), 787 (m), 769 (s), 742 (m), 660 (w), 644 (w), 609 (m) $cm^{-1}$.

UV/vis ($CH_2Cl_2$, rt): $\lambda_{max}$ (ε) 287 (13900), 333 (14600), 360 (16200), 378 (23000), 450 (2800), 478 (2700) nm ($M^{-1}$ $cm^{-1}$).

Fluorescence ($CH_2Cl_2$, rt): $\lambda_{ex}$ = 395 nm, $\lambda_{em}$ = 501 nm, $\phi_F$: 0.11.

HRMS (MALDI, DCTB) m/z: [$M^+$] Calcd for $C_{48}H_{34}{}^{79}Br_2$ 768.1022; Found 768.1015.

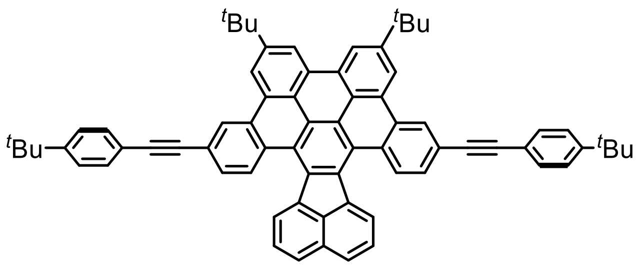


**6**

**12,15-Di-*tert*-butyl-9,18-bis[(4-*tert*-butylphenyl)ethynyl]dibenzo[*fg*,*ij*]fluoranthene [7,8,9,10-*rst*]pentaphene** (**6**). Compound **5** (50.0 mg, 64.9 µmol) and *p*-(*tert*-butyl)-ethynylbenzene (58.5 µL, 51.3 mg, 324 µmol) were dissolved in $Et_3N$/THF (5 mL, 2:1) and purged with Ar for 10 min. [$PdCl_2(PPh_3)_2$] (2.28 mg, 3.24 µmol) and CuI (2.47 mg, 13.0 µmol) were added, and the reaction mixture was stirred for 18 h at 100 °C in an aluminum heating block. After cooling to rt, the reaction mixture was extracted with $CH_2Cl_2$ (3×75.0 mL) and washed with sat. aq. $NH_4Cl$ (50.0 ml). The combined organic phases were dried over $Na_2SO_4$, filtered, and the solvent was removed under reduced pressure. The crude product was purified by column chromatography ($SiO_2$, hexanes/$CH_2Cl_2$ 9:1) to obtain **10.9** (39.0 mg, 42.2 µmol, 65%) as yellow solid upon evaporation of the solvent.

M.p.: 340–342 °C.

$R_f$ = 0.42 ($SiO_2$, PE/$CH_2Cl_2$ 3:1).

$^1$H NMR (400 MHz, $CDCl_3$): $\delta$ = 9.49 (d, $J$ = 8.1 Hz, 2H), 9.12 (s, 2H), 8.96 (d, $J$ = 7.1 Hz, 4H), 8.74 (d, $J$ = 7.1 Hz, 2H), 7.86–7.80 (m, 4H), 7.64 (d, $J$ = 8.4 Hz, 4H), 7.53 (t, $J$ = 7.6 Hz, 2H), 7.46 (d, $J$ = 8.5 Hz, 4H), 1.75 (s, 18H), 1.38 (s, 18H) ppm.

$^{13}$C NMR (126 MHz, $CDCl_3$): $\delta$ = 151.9, 149.4, 137.7, 133.8, 132.3, 131.7, 131.6, 130.2, 129.7, 129.5, 129.3, 128.5, 127.6, 127.3, 127.0, 125.8, 125.6, 124.0, 123.4, 123.0, 120.4, 119.4, 119.1, 117.3, 90.9, 89.7, 35.9, 35.0, 32.1, 31.4 ppm (one aromatic signal coincident or not observed).

IR (FT-ATR): $\tilde{\nu}$ = 3056 (w), 2954 (s), 2922 (s), 2852 (s), 1605 (m), 1583 (m), 1516 (m), 1459 (m), 1431 (m), 1392 (m), 1376 (m), 1362 (m), 1265 (m), 1244 (m), 1198 (m), 1103 (m), 1016 (m), 952 (w), 867 (m), 850 (m), 832 (m), 819 (m), 767 (m) cm$^{-1}$.

UV/vis ($CH_2Cl_2$, rt): $\lambda_{max}$ (ε) 272 (38600), 310 (32400), 388 (31100), 423 (21600), 489 (2600) nm (M$^{-1}$ cm$^{-1}$).

Fluorescence ($CH_2Cl_2$, rt): $\lambda_{ex}$ = 375 nm, $\lambda_{em}$ = 514 nm, $\phi_F$: 0.25.

HRMS (MALDI, DCTB) m/z: [M$^+$] Calcd for $C_{72}H_{60}$ 924.4690; Found 924.4689.

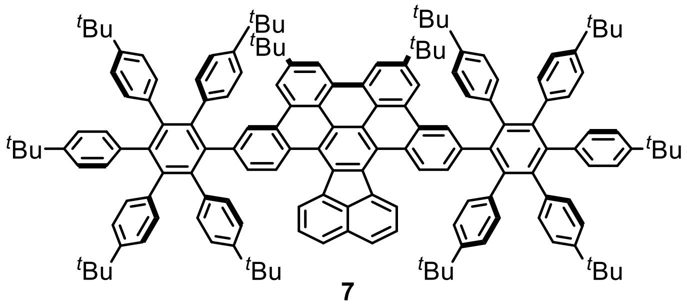

**7**

**Compound 7**. A microwave tube was charged with **6** (41.0 mg, 44.3 µmol) and *p-tert*-butyl-tetracyclone **S1** (67.5 mg, 111 µmol) under argon atmosphere. The sealed tube was maintained at 250 °C in a sand bath for 18 h. After cooling to rt, the crude material was subjected to column chromatography ($SiO_2$, hexanes/$CH_2Cl_2$ 9:1) to afford the desired compound **7** (66.0 mg, 31.6 µmol, 71%) as yellow crystalline solid upon evaporation of the solvent.

M.p.: >400 °C.

$R_f$ = 0.44 ($SiO_2$, PE/$CH_2Cl_2$ 3:1).

$^1$H NMR (700 MHz, $CD_2Cl_2$): $\delta$ = 8.97 (d, $J$ = 8.3 Hz, 2H), 8.96 (d, $J$ = 0.6 Hz, 2H), 8.47 (s, 2H), 8.29 (d, $J$ = 7.2 Hz, 2H), 8.17 (s, 2H), 7.75 (d, $J$ = 8.0 Hz, 2H), 7.37 (t, $J$ = 7.6 Hz, 2H), 7.21 (dd, $J$ = 8.3, 1.5 Hz, 2H), 6.94–6.89 (m, 20H), 6.86–6.83 (m, 10H), 6.80 (d, $J$ = 8.0 Hz, 10H), 1.68 (s, 18H), 1.15 (s, 18H), 1.14 (s, 72H) ppm.

$^{13}$C NMR (176 MHz, $CD_2Cl_2$): δ = 149.2, 148.5, 148.3, 141.34, 141.29, 141.2, 141.0, 140.7, 138.43, 138.36, 133.5, 132.6, 131.7, 131.5, 131.4, 130.39, 130.38, 130.0, 129.7, 129.6, 127.91, 127.90, 127.7, 127.4, 127.0, 125.9, 123.84, 123.79, 123.68, 123.66, 123.4, 122.6, 119.2, 118.8, 35.9, 34.42, 34.42, 32.1, 31.4 ppm (three aromatic and three aliphatic signals coincident or not observed).

IR (FT-ATR): $\tilde{\nu}$ = 3086 (w), 3053 (w), 3035 (w), 2957 (s), 2923 (s), 2854 (s), 1609 (m), 1584 (m), 1510 (m), 1461 (m), 1425 (m), 1392 (m), 1362 (m), 1267 (m), 1201 (m), 1153 (m), 1117 (m), 1102 (m), 1018 (m), 870 (m), 859 (m), 832 (s), 823 (m), 774 (m), 740 (m), 639 (m) $cm^{-1}$.

UV/vis ($CH_2Cl_2$, rt): $\lambda_{max}$ (ε) 383 (38400), 457 (4300), 490 (4200) nm ($M^{-1}$ $cm^{-1}$).

Fluorescence ($CH_2Cl_2$, rt): $\lambda_{ex}$ = 383 nm, $\lambda_{em}$ = 528 nm, $\phi_F$: 0.28.

HRMS (MALDI, DCTB) m/z: [$M^+$] Calcd for $C_{160}H_{164}$ 2085.2828; Found 2085.2807.

**Compounds 1 and 2**. Compound **7** (10.0 mg, 4.79 µmol) and DDQ (14.4 mg, 63.3 µmol) were dissolved in dry $CH_2Cl_2$ (20.0 mL). The solution was cooled to 0 °C and deoxygenated with nitrogen for 10 min. To the cooled solution TfOH (200 µL, 1 v%) was added dropwise over 5 min under continuous stirring. After additional 30 min of stirring at this temperature the reaction mixture was quenched via dropwise addition of sat. aq. $Na_2CO_3$ (50.0 mL). The reaction mixture was extracted with $CH_2Cl_2$ (3×100 mL), dried over $Na_2SO_4$, and filtered. The solvent was evaporated under reduced pressure to give the crude material which was firstly purified by column chromatography ($SiO_2$, hexanes/$CH_2Cl_2$ 9:1) to obtain a mixture of **1** and **2** that was further purified as indicated.

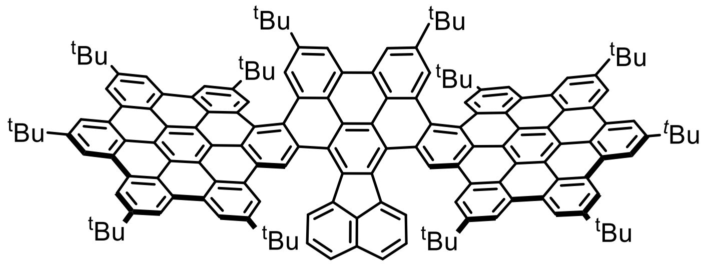


**1**

**Compound 1**. After preparative TLC (hexanes/EtOAc 9:1), from the TLC plate the fluorescent baseline spot was scratched off and eluted with $CH_2Cl_2$ upon filtration. Subsequently, the solvent was evaporated to obtain **1** (6.00 mg, 2.91 µmol, 61%) as orange solid.

M.p.: >400 °C.

$R_f$ = 0.14 ($SiO_2$, PE/$CH_2Cl_2$ 3:1).

$^1$H NMR (500 MHz, $CDCl_3$): $\delta$ = 11.17 (s, 2H), 9.69 (s, 2H), 9.43 (s, 2H), 9.40 (s, 10H), 9.38 (s, 2H), 9.36 (s, 2H), 9.32 (s, 2H), 9.25 (s, 2H), 9.08 (d, $J$ = 7.6 Hz, 2H), 9.04 (s, 2H), 7.91 (d, $J$ = 8.0 Hz, 2H), 7.43 (t, $J$ = 7.8 Hz, 2H), 1.91 (s, 18H), 1.89 (s, 18H), 1.87 (s, 18H), 1.76 (s, 18H), 1.54 (s, 18H), 1.34 (s, 18H) ppm.

$^{13}$C NMR (126 MHz, $CDCl_3$) $\delta$ 150.1, 149.6, 149.5, 149.4, 148.0, 145.2, 138.4, 137.1, 135.5, 133.9, 131.8, 131.2, 131.1, 130.9, 130.9, 130.7, 130.7, 130.7, 130.6, 130.5, 130.4, 129.4, 128.5, 128.1, 128.1, 127.8, 127.4, 127.0, 126.6, 125.4, 124.6, 124.4, 124.3, 124.3, 124.3, 123.4, 123.0, 121.3, 121.2, 121.1, 121.1, 120.6, 120.5, 120.2, 119.7, 119.6, 119.3, 119.2, 36.0, 36.0, 36.0, 35.6, 35.4, 32.3, 32.2, 32.2, 32.2, 32.0, 31.8 (nine aromatic and one aliphatic signals coincident or not observed).

IR (FT-ATR): $\tilde{\nu}$ = 2953 (s), 2922 (s), 2852 (s), 1659 (w), 1634 (w), 1605 (w), 1577 (w), 1459 (m), 1375 (m), 1260 (m), 941 (w), 869 (m), 821 (w), 770 (m), 748 (m), 629 (w) cm$^{-1}$.

UV/vis ($CH_2Cl_2$, rt): $\lambda_{max}$ (ε) 372 (178800), 417 (173400), 446 (171700) nm (M$^{-1}$ cm$^{-1}$).

Fluorescence ($CH_2Cl_2$, rt): $\lambda_{ex}$ = 448 nm, $\lambda_{em}$ = 532 nm, $\phi_F$: 0.15.

HRMS (MALDI, DCTB) m/z: [M$^+$] Calcd for $C_{160}H_{140}$ 2061.0950; Found 2061.0880.

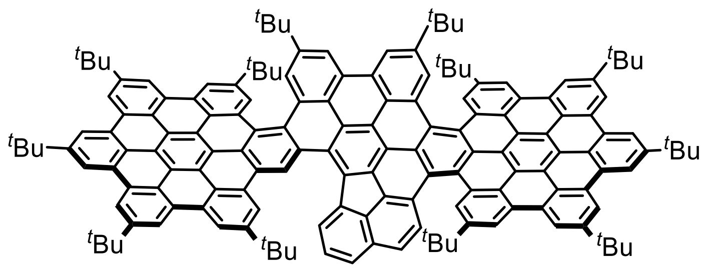


**2**

**Compound 2**. After preparative TLC (hexanes/$CH_2Cl_2$ 8:2), additional HPLC (Agilent Zorbax Rx-Sil, 250 mm, 20.0 mL $min^{-1}$, hexanes/$CH_2Cl_2$ 9:1) obtained **2** (1.00 mg, 0.48 µmol, 10%) as red solid. Separation of the enantiomers was achieved upon chiral HPLC (CHIRALPAK IA-5, 250 mm, 24.0 mL $min^{-1}$, heptane/*i*-propanol 85:15).

M.p.: >400 °C.

$R_f$ = 0.16 ($SiO_2$, PE/$CH_2Cl_2$ 3:1).

$^1$H NMR (500 MHz, $CD_2Cl_2$): $\delta$ = 11.40 (s, 1H), 9.70 (s, 1H), 9.58 (s, 2H), 9.54 (s, 1H), 9.51 (s, 2H), 9.49 (d, *J* = 4.5 Hz, 2H), 9.47–9.45 (m, 8H), 9.43 (s, 1H), 9.41 (s, 2H), 9.40 (s, 2H), 9.27 (s, 1H), 9.26 (s, 1H), 9.23 (s, 1H), 9.21 (s, 1H), 8.91 (d, *J* = 8.8 Hz, 1H), 8.24 (d, *J* = 7.6 Hz, 1H), 8.17 (d, *J* = 8.8 Hz, 1H), 8.00 (s, 1H), 1.92 (s, 9H), 1.91 (s, 9H), 1.91 (s, 9H), 1.90 (s, 9H), 1.90 (s, 9H), 1.89 (s, 9H), 1.79 (s, 9H), 1.60 (s, 9H), 1.48 (s, 9H), 1.45 (s, 9H), 1.43 (s, 9H), 1.37 (s, 9H) ppm.

$^{13}$C NMR: the available quantity did not allow to acquire a meaningful spectrum.

IR (FT-ATR): $\tilde{\nu}$ = 2957 (s), 2923 (s), 2867 (m), 1685 (w), 1604 (m), 1578 (m), 1477 (m), 1464 (m), 1393 (m), 1368 (m), 1347 (m), 1307 (m), 1259 (m), 1229 (m), 1202 (m), 1099 (w), 1024 (w), 941 (w), 871 (m), 824 (m), 810 (m), 632 (m) $cm^{-1}$.

UV/vis ($CH_2Cl_2$, rt): $\lambda_{max}$ (ε) 362 (117700), 382 (115700), 406 (109500), 435 (114500), 530 (43600), 572 (60000) nm ($M^{-1}$ $cm^{-1}$).

Fluorescence ($CH_2Cl_2$, rt): $\lambda_{ex}$ = 439 nm, $\lambda_{em}$ = 601 nm, $\phi_F$: 0.42.

HRMS (MALDI, DCTB) m/z: [$M^+$] Calcd for $C_{160}H_{138}$ 2059.0793; Found 2059.0787.

## 3. NMR data

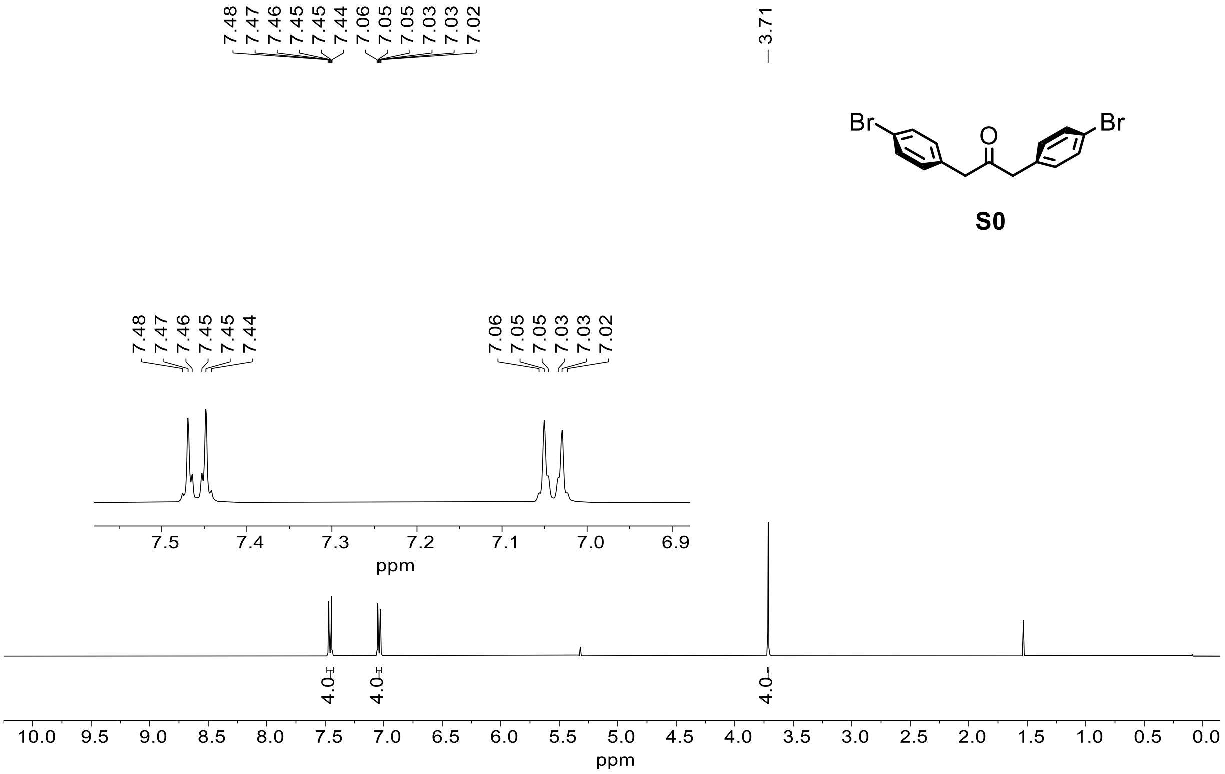


**Fig. S1** $^{1}$H NMR spectrum of compound **S0** (400 MHz, $CDCl_3$, °$H_2O$).

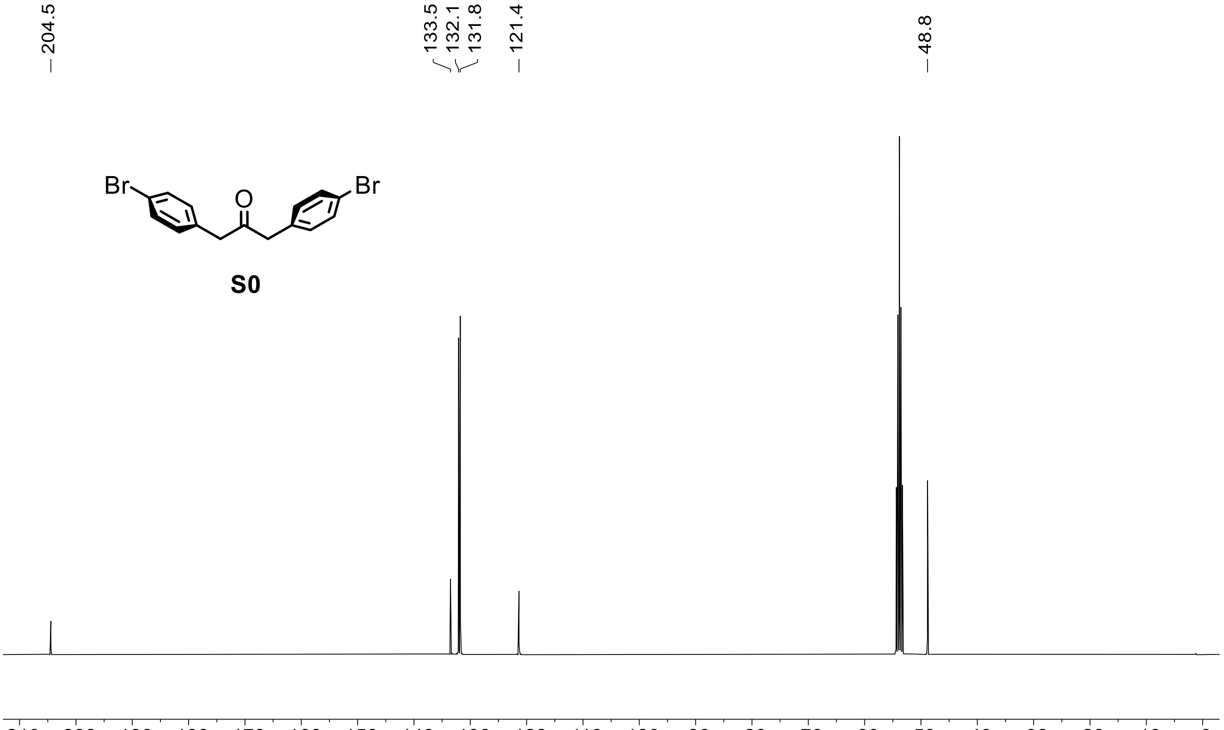


**Fig. S2** $^{13}$C NMR spectrum of compound **S0** (101 MHz, $CDCl_3$).

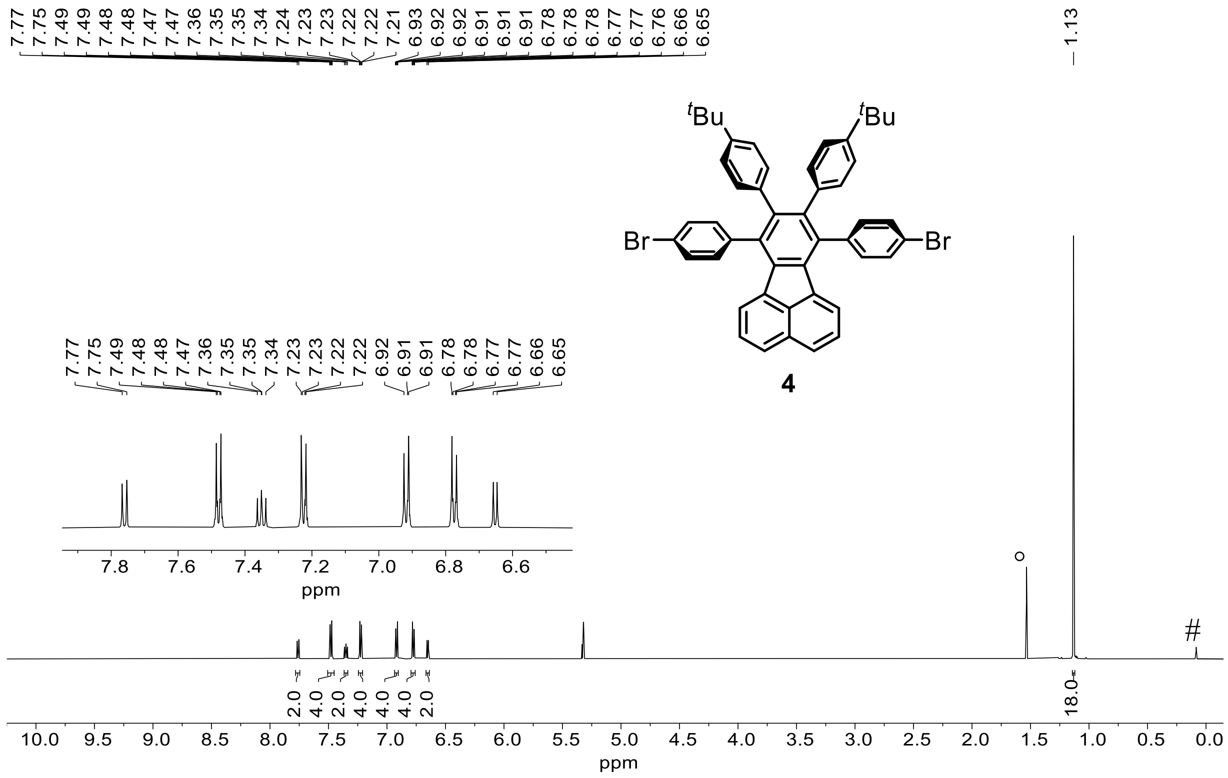

**Fig. S3** $^{1}$H NMR spectrum of compound **4** (600 MHz, $CD_2Cl_2$, °$H_2O$, #silicone grease).

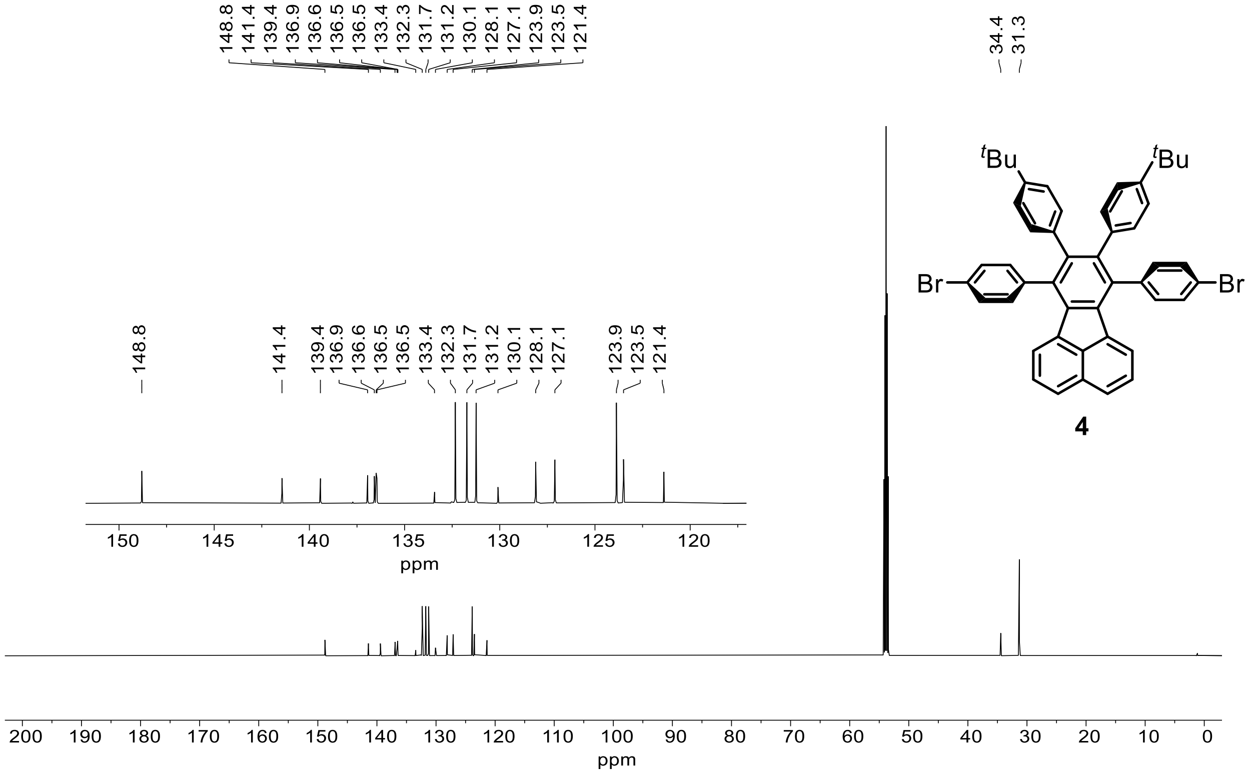

**Fig. S4** $^{13}$C NMR spectrum of compound **4** (151 MHz, $CD_2Cl_2$).

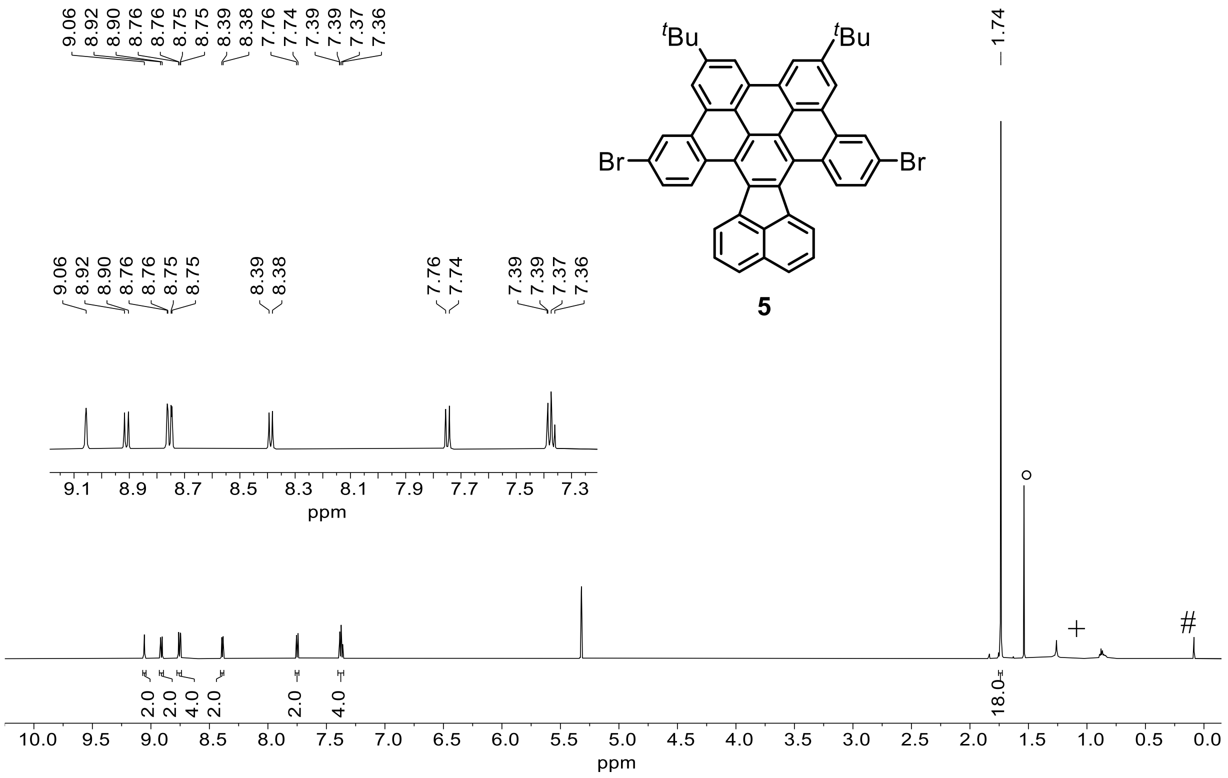


**Fig. S5** $^{1}H$ NMR spectrum of compound **5** (600 MHz, $CD_2Cl_2$, °$H_2O$, $^{+}$hexanes, $^{\#}$silicone grease).

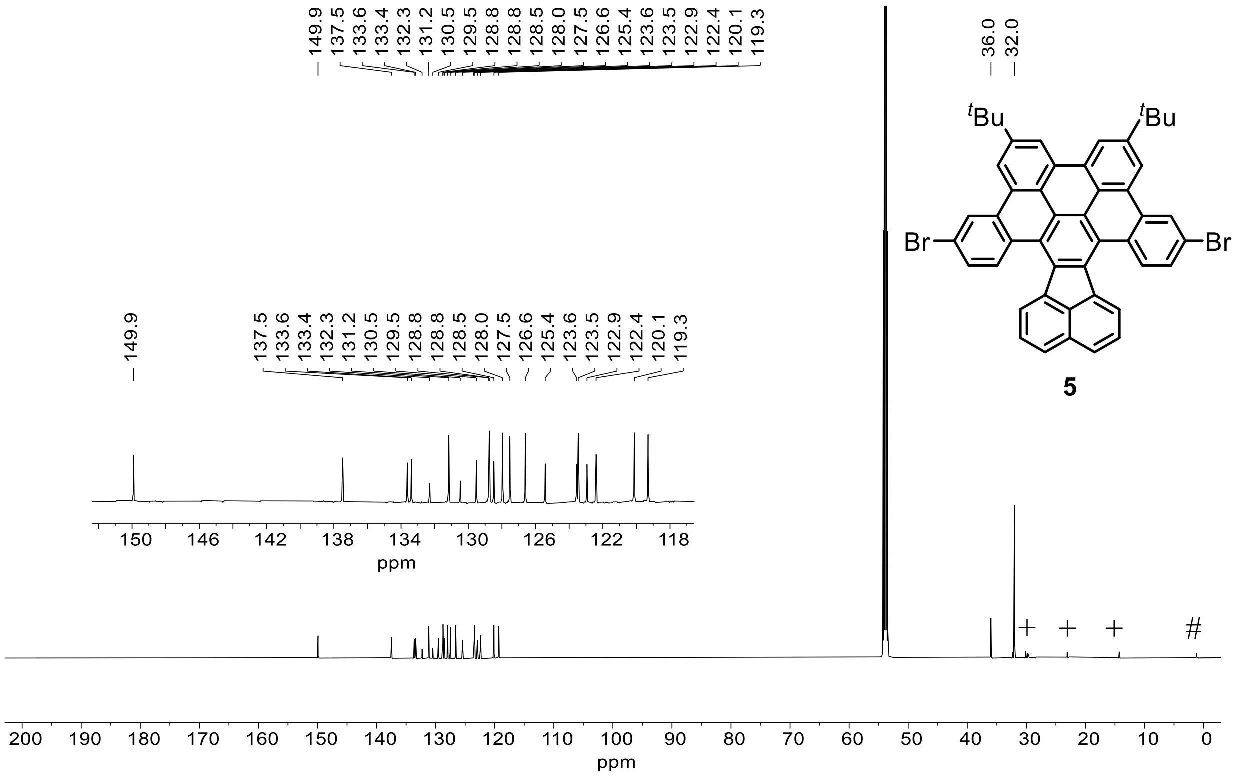


**Fig. S6** $^{13}C$ NMR spectrum of compound **5** (151 MHz, $CD_2Cl_2$, $^{+}$hexanes, $^{\#}$silicone grease).

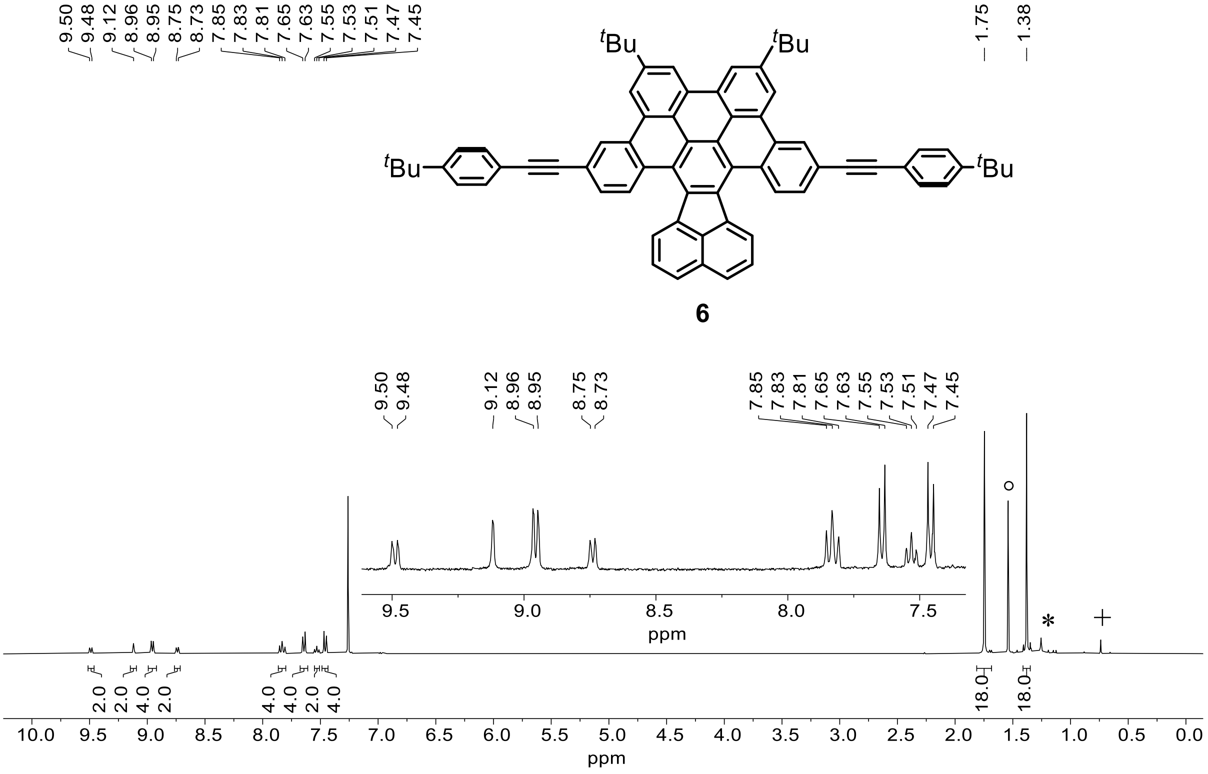


**Fig. S7** $^{1}H$ NMR spectrum of compound **6** (400 MHz, $CDCl_3$, °$H_2O$, *PE, ⁺hexanes).

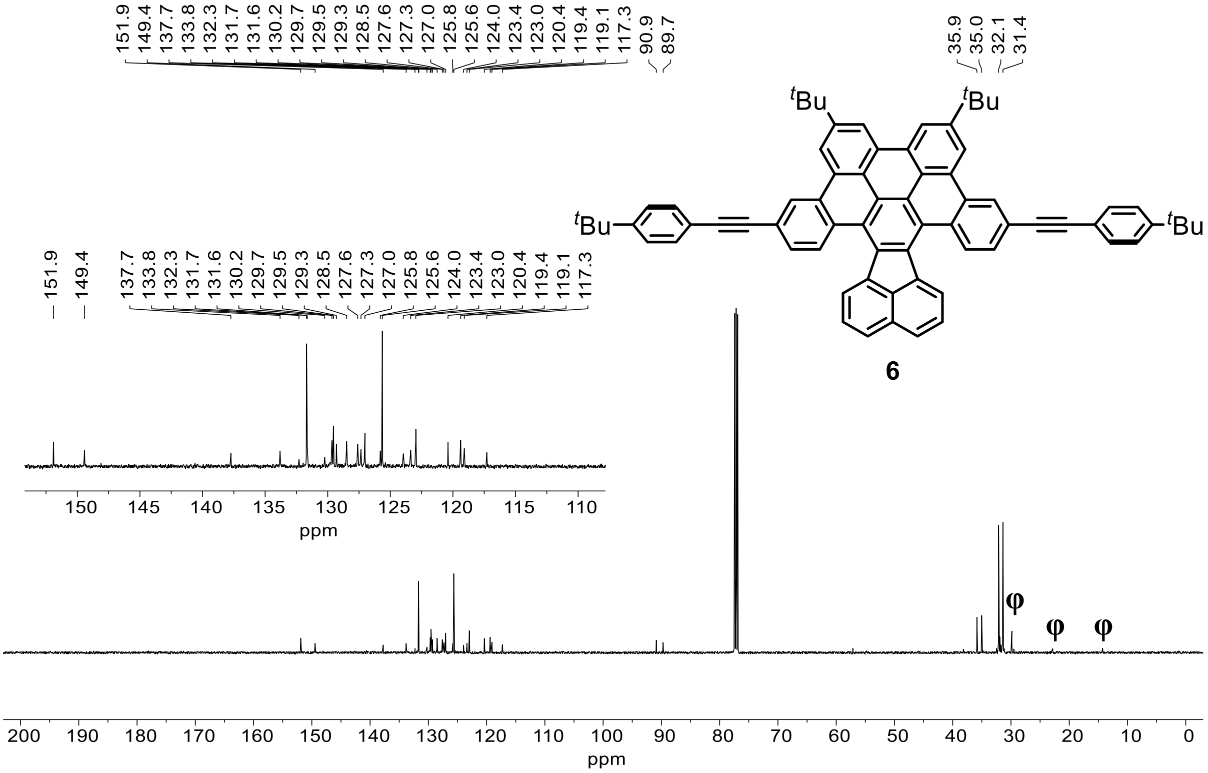


**Fig. S8** $^{13}C$ NMR spectrum of compound **6** (126 MHz, $CDCl_3$, $^{\Phi}$hexanes).

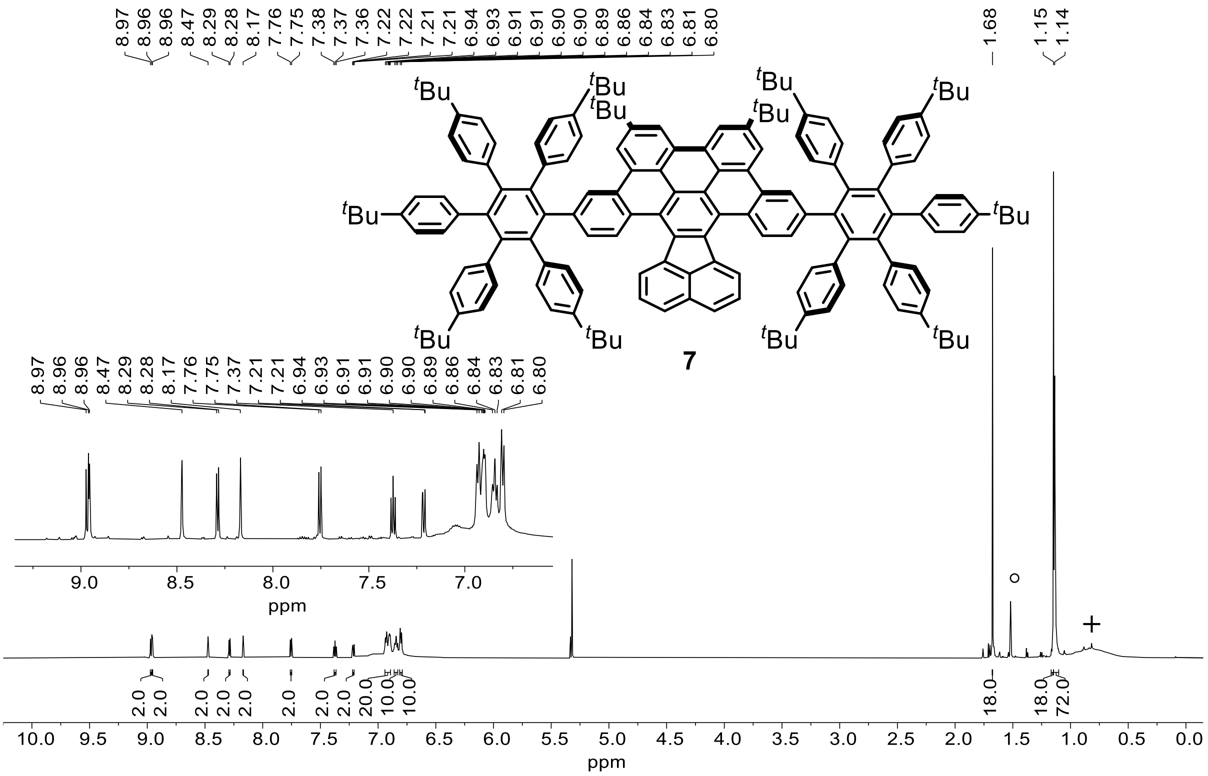


**Fig. S9** $^1$H NMR spectrum of compound **7** (700 MHz, $CD_2Cl_2$, °$H_2O$, $^+$hexanes).

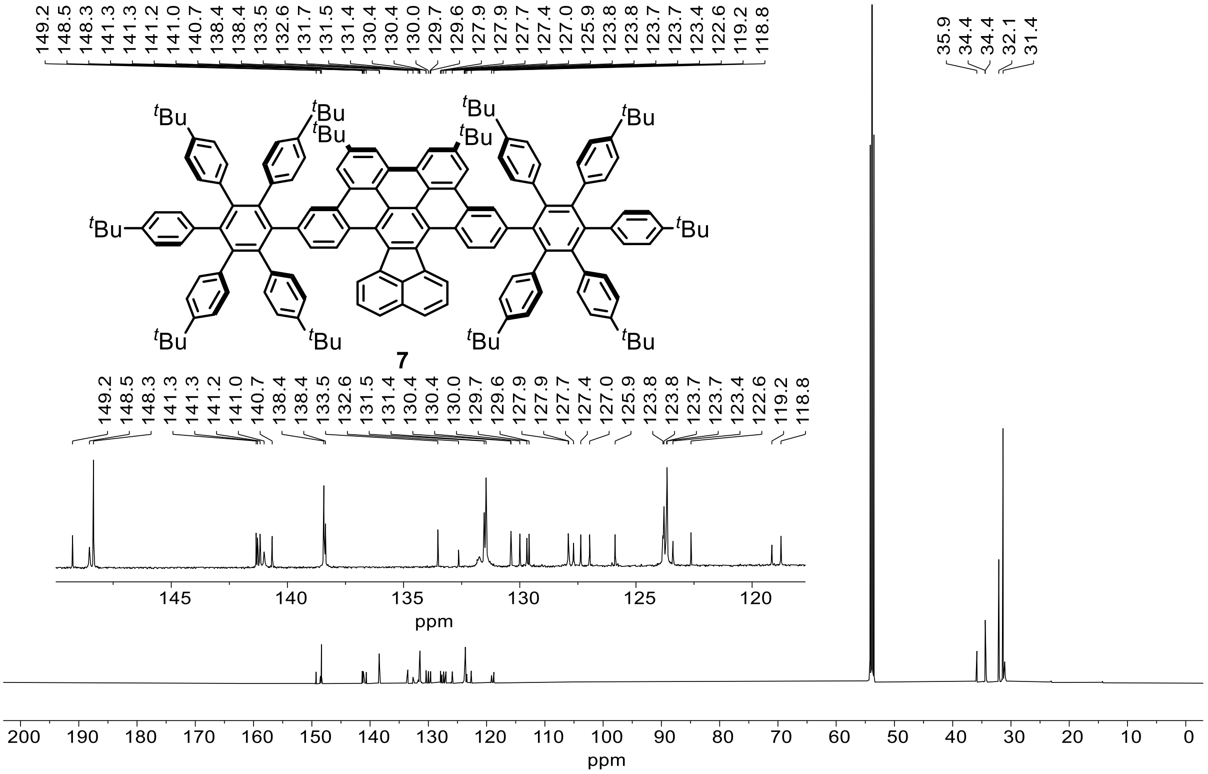


**Fig. S10** $^{13}$C NMR spectrum of compound **7** (176 MHz, $CD_2Cl_2$).

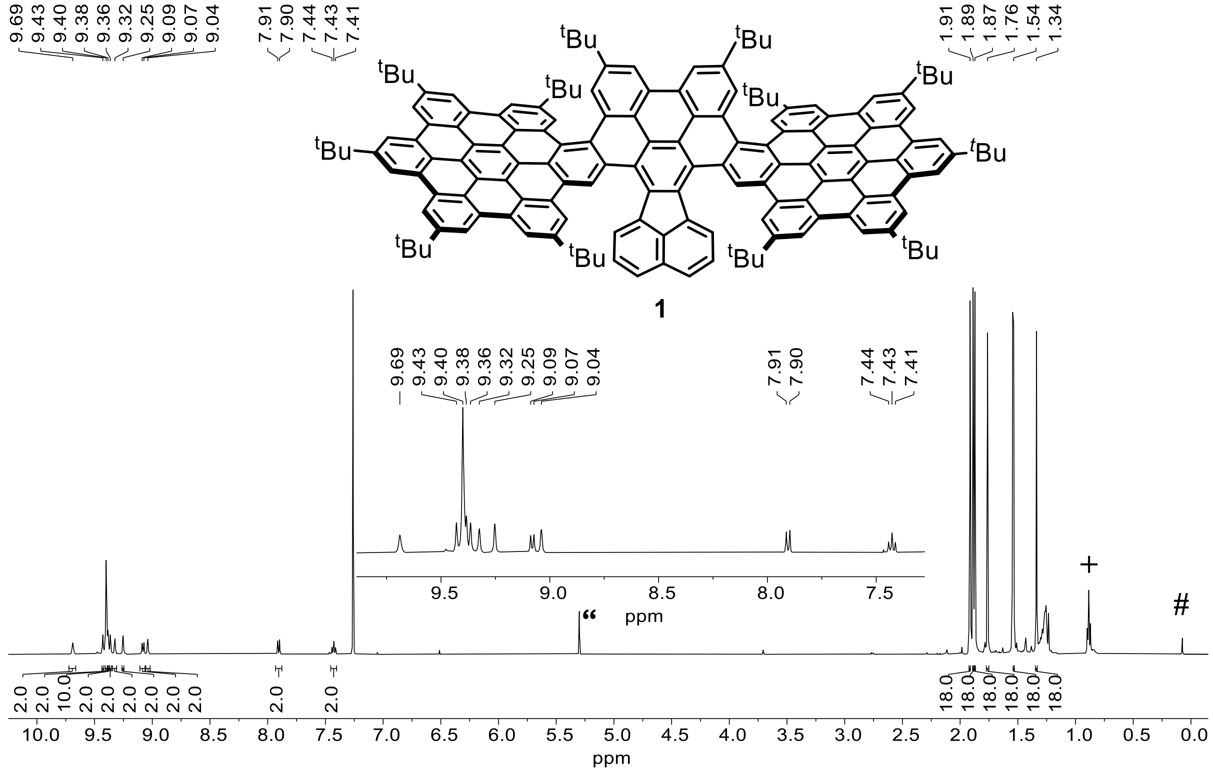


**Fig. S11** $^{1}H$ NMR spectrum of compound **1** (500 MHz, $CDCl_3$, “$CH_2Cl_2$, $^{+}$hexanes, $^{\#}$silicone grease).

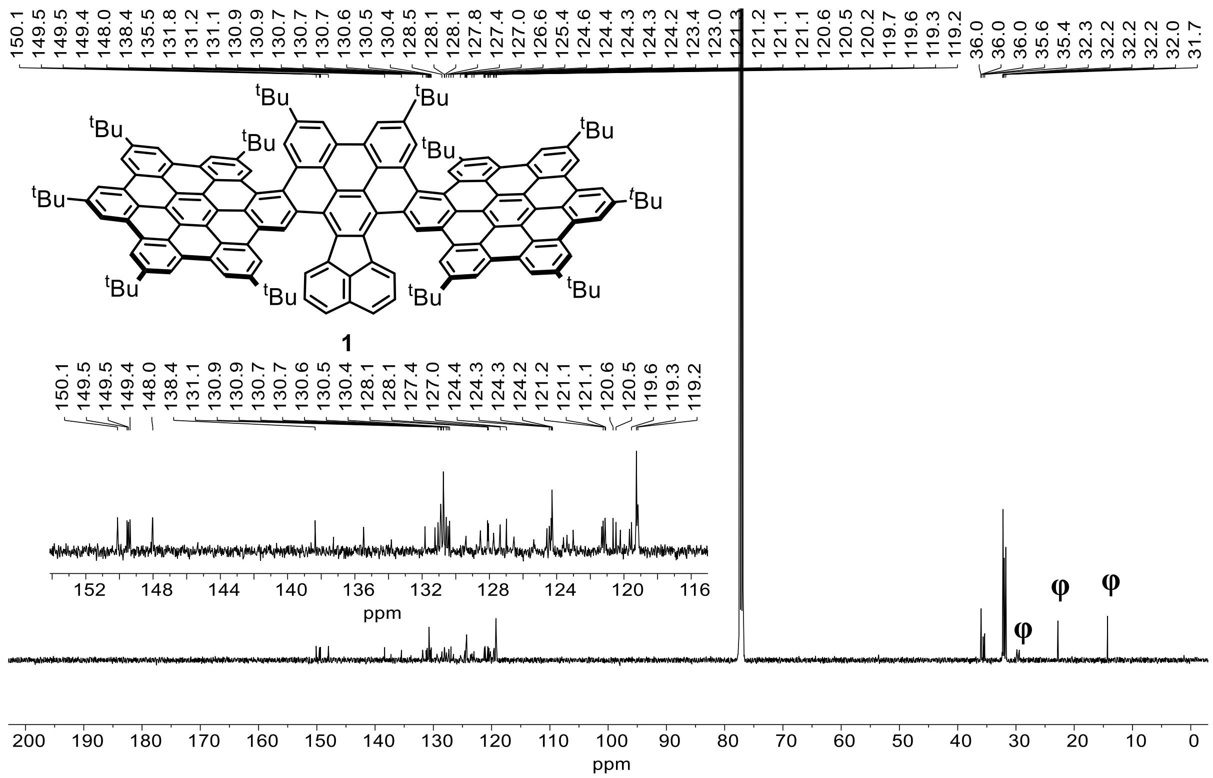


**Fig. S12** $^{13}C$ NMR spectrum of compound **1** (126 MHz, $CDCl_3$, $^{\Phi}$hexanes).

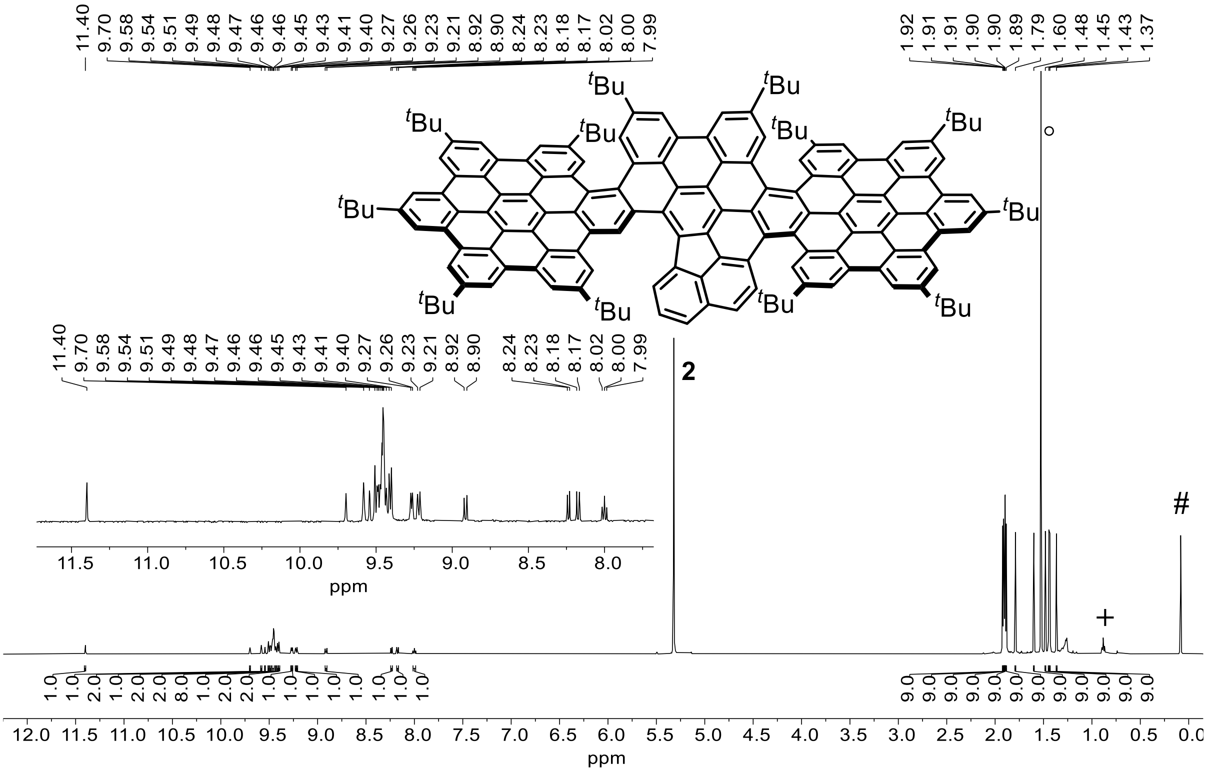

**Fig. S13** $^{1}$H NMR spectrum of compound **2** (500 MHz, $CD_2Cl_2$, °$H_2O$, $^{+}$hexanes, $^{\#}$silicone grease).

## 4. HR-MS data

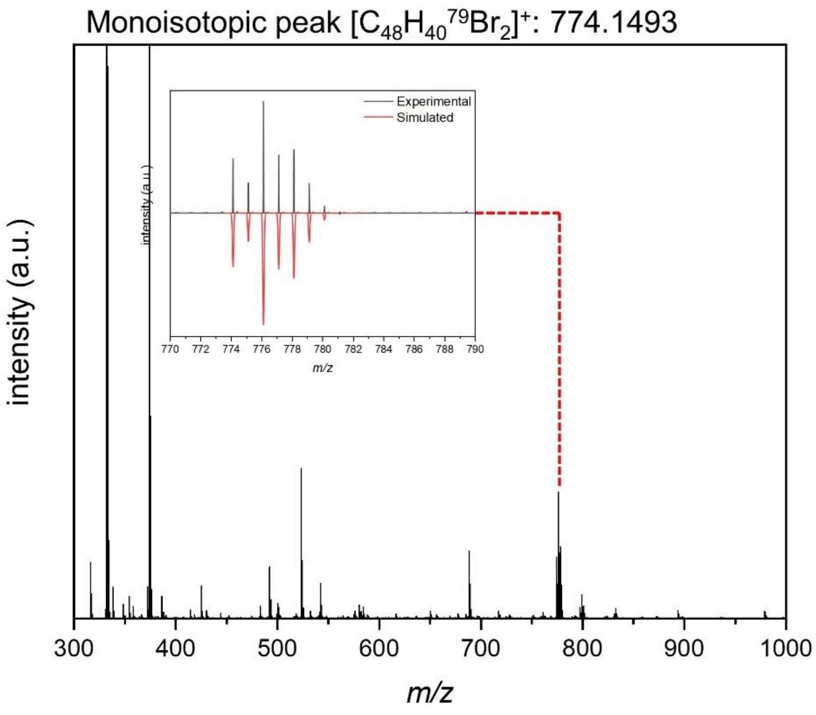


**Fig. S14** HR-MS (MALDI, DCTB) spectrum of **4**. Inset: experimental (black) and theoretical (red) isotope distribution of the signal corresponding to **4**.

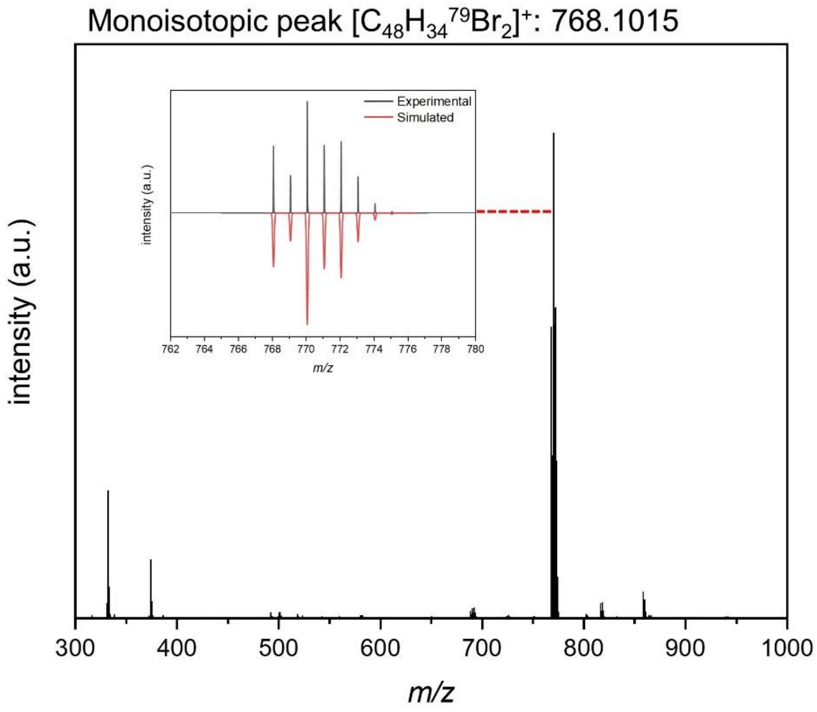


**Fig. S15** HR-MS (MALDI, DCTB) spectrum of **5**. Inset: experimental (black) and theoretical (red) isotope distribution of the signal corresponding to **5**.

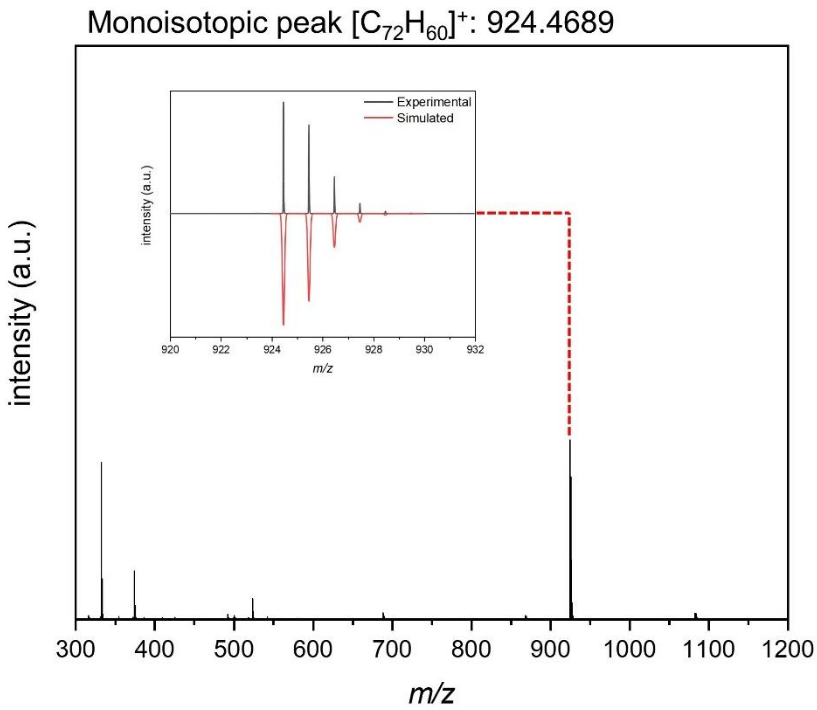


**Fig. S16** HR-MS (MALDI, DCTB) spectrum of **6**. Inset: experimental (black) and theoretical (red) isotope distribution of the signal corresponding to **6**.

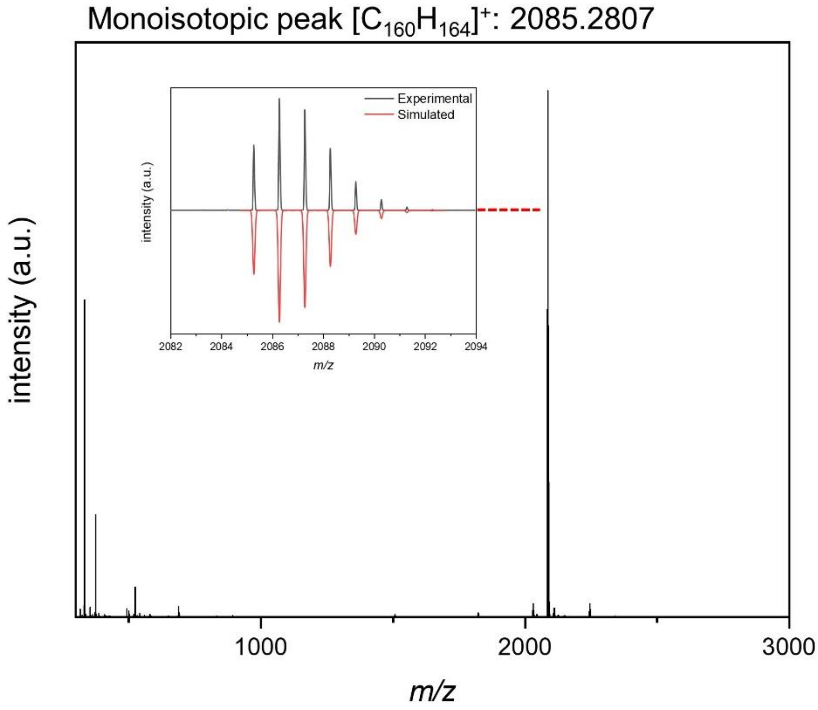


**Fig. S17** HR-MS (MALDI, DCTB) spectrum of **7**. Inset: experimental (black) and theoretical (red) isotope distribution of the signal corresponding to **7**.

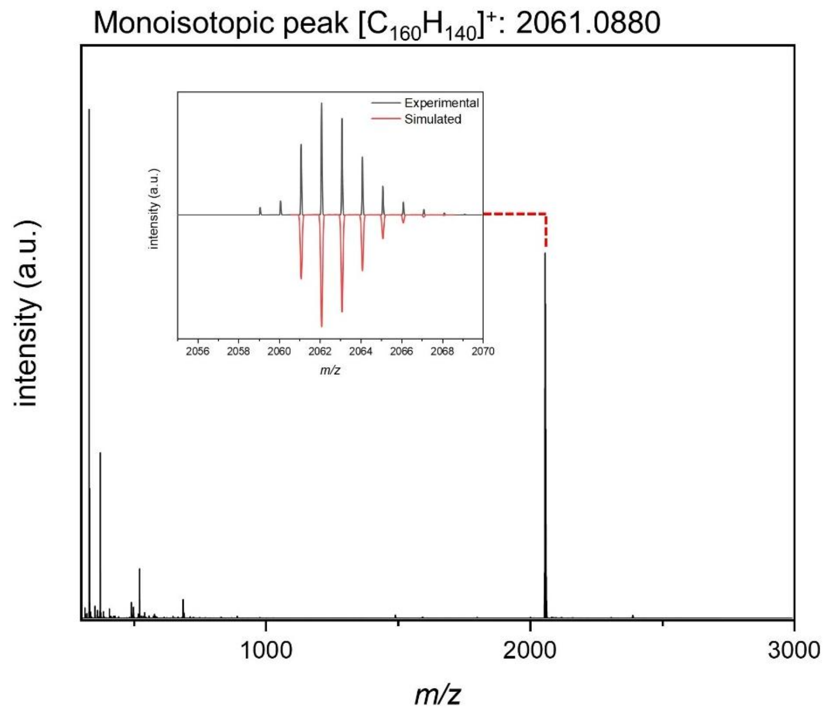


**Fig. S18** HR-MS (MALDI, DCTB) spectrum of **1**. Inset: experimental (black) and theoretical (red) isotope distribution of the signal corresponding to **1**.

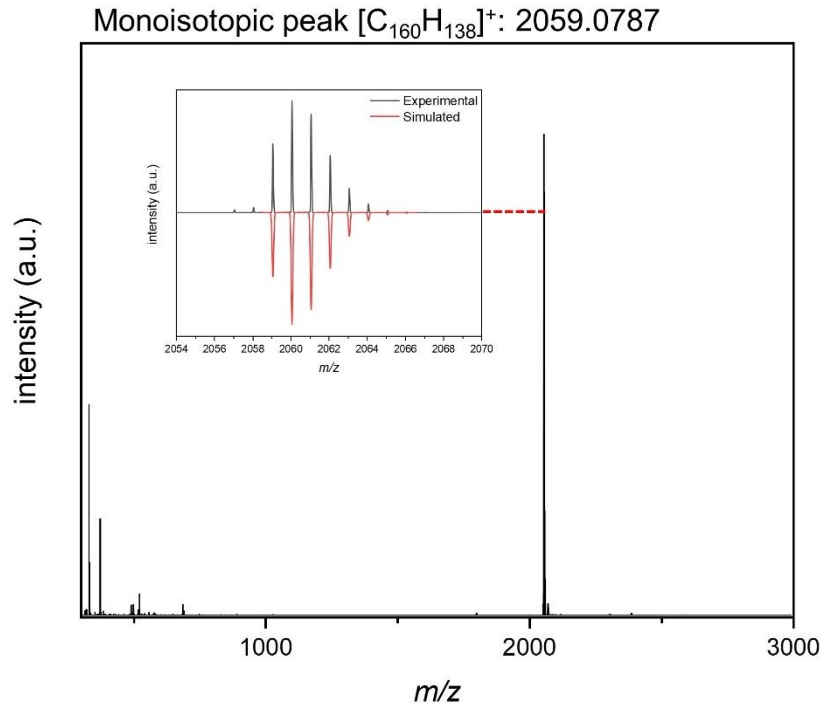


**Fig. S19** HR-MS (MALDI, DCTB) spectrum of **2**. Inset: experimental (black) and theoretical (red) isotope distribution of the signal corresponding to **2**.

## 5. Crystallographic Data

Compound **4** was crystallized from slow gas phase diffusion of MeOH into a saturated solution of the compound in toluene at rt. Species **4** crystallizes as yellow plates in the monoclinic space group *P2₁/n*.

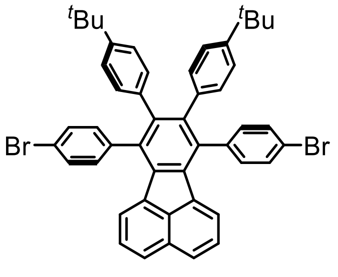


**4**

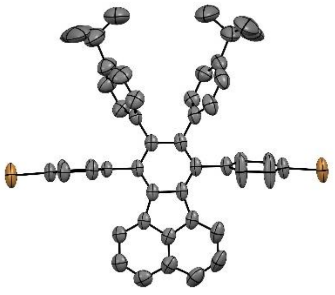

**Table S1.** Crystal data and structure refinement for **4** (ellipsoids at 50% probability).

| | | | |
|---|---|---|---|
| CCDC | | 2407547 | |
| Empirical Formula | | $C_{48}H_{40}Br_2$ | |
| Formula Weight | | 776.62 | |
| Temperature / K | | 200(2) | |
| Wavelength / Å | | 1.54178 | |
| Crystal System | | monoclinic | |
| Space Group | | *P2₁/n* | |
| Z | | 4 | |
| *a* / Å | *α* / deg | 19.9815(10) | 90 |
| *b* / Å | *β* / deg | 12.3000(3) | 114.727(3) |
| *c* / Å | *γ* / deg | 20.3827(8) | 90 |
| Volume / $Å^3$ | | 4550.2(3) | |
| Density (Calculated) / $g/cm^3$ | | 1.13 | |
| Absorption Coefficient / $mm^{-1}$ | | 2.46 | |
| Crystal Shape (Color) | | plate (pale yellow) | |
| Crystal Size / $mm^3$ | | 0.219 × 0.081 × 0.024 | |
| Theta Range for Data Collection / deg | | 2.6 to 68.4 | |
| Index Ranges | | –23≤h≤20, –14≤k≤6, –24≤l≤24 | |
| Reflections Collected | | 27975 | |
| Reflections (Independent) | | 8155 (R(int) = 0.0360) | |
| Reflections (Observed) | | 4774 (I > 2σ(I)) | |
| Goodness-of-fit on $F^2$ | | 1.00 | |
| Final R Indices (I > 2sigma(I)) | | $R_1$ = 0.043, $wR_2$ = 0.091 | |
| Largest Diff. Peak and Hole / $eÅ^{-3}$ | | 0.46 and –0.41 | |

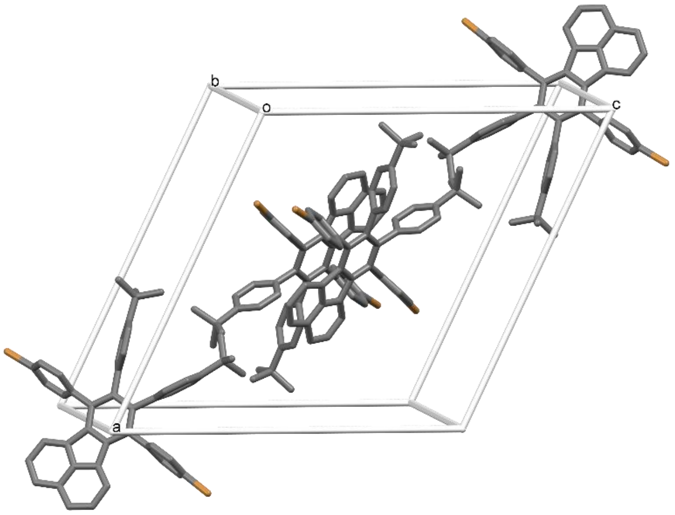


**Fig. S20** Unit cell of **4** along the b-axis. Hydrogen atoms omitted for clarity.

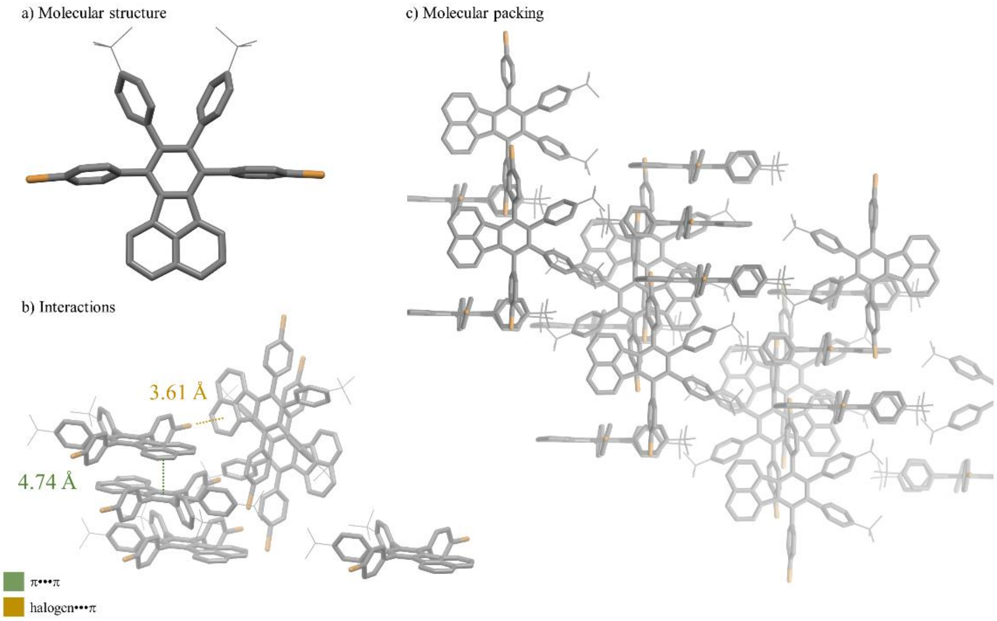


**Fig. S21** X-ray crystal structure of **4**. a) Front view. b) π···π interactions (green) and halogen···π interactions (yellow) between individual molecules of **4**. c) Virtual arrangement of the packing motif. Color code: carbon (grey), bromine (orange). Hydrogen atoms and solvent molecules are omitted for clarity; *tert*-butyl groups are depicted as wire.

Compound **6** was crystallized from slow gas phase diffusion of MeOH into a saturated solution of the compound in toluene at rt. Species **6** crystallizes as orange columns in the monoclinic space group *C2/c*.

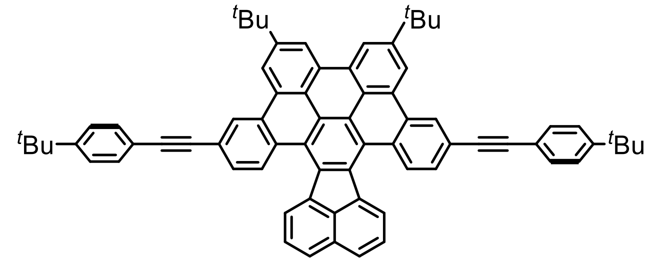


**6**

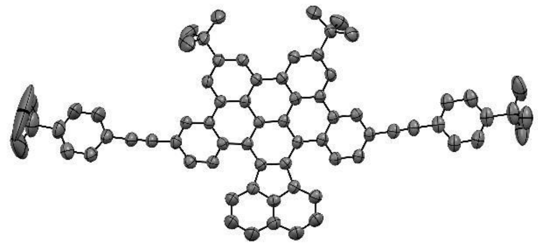

**Table S1.** Crystal data and structure refinement for **6** (ellipsoids at 50% probability).

| | | | |
|---|---|---|---|
| CCDC | | 2407548 | |
| Empirical Formula | | $C_{72}H_{60}$ | |
| Formula Weight | | 925.20 | |
| Temperature / K | | 200(2) | |
| Wavelength / Å | | 1.54178 | |
| Crystal System | | monoclinic | |
| Space Group | | *C2/c* | |
| Z | | 8 | |
| *a* / Å | *α* / deg | 31.8938(12) | 90 |
| *b* / Å | *β* / deg | 22.0783(10) | 95.247(3) |
| *c* / Å | *γ* / deg | 17.0400(7) | 90 |
| Volume / Å$^3$ | | 11948.6(9) | |
| Density (Calculated) / g/cm$^3$ | | 1.03 | |
| Absorption Coefficient / mm$^{-1}$ | | 0.44 | |
| Crystal Shape (Color) | | column (orange) | |
| Crystal Size / mm$^3$ | | 0.248 × 0.065 × 0.050 | |
| Theta Range for Data Collection / deg | | 2.4 to 66.6 | |
| Index Ranges | | –37≤h≤30, –26≤k≤22, –12≤l≤20 | |
| Reflections Collected | | 40296 | |
| Reflections (Independent) | | 10411 (R(int) = 0.0872) | |
| Reflections (Observed) | | 6158 (I > 2σ(I)) | |
| Goodness-of-fit on F$^2$ | | 1.05 | |
| Final R Indices (I > 2sigma(I)) | | $R_1$ = 0.093, $wR_2$ = 0.225 | |
| Largest Diff. Peak and Hole / eÅ$^{-3}$ | | 0.45 and –0.35 | |

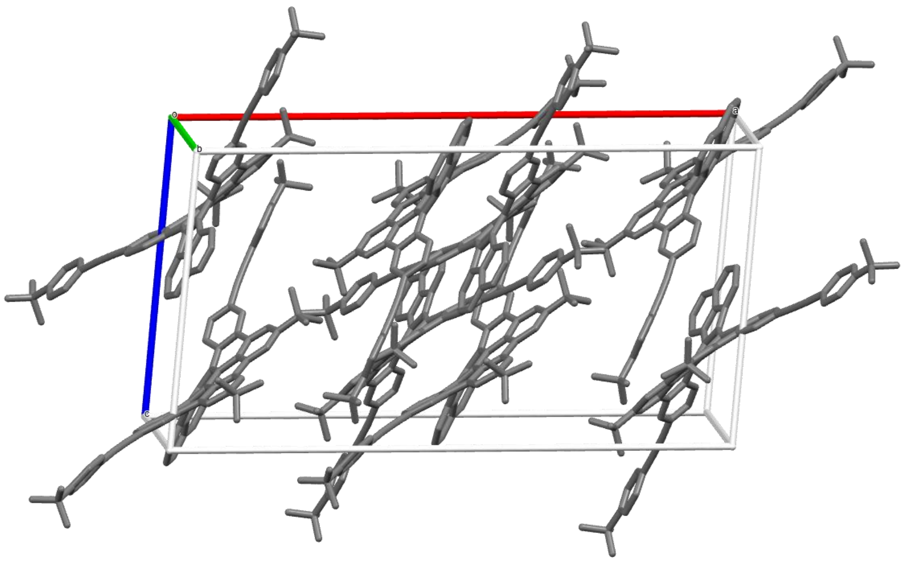

**Fig. S22** Unit cell of **6** along the b-axis. Hydrogen atoms omitted for clarity.

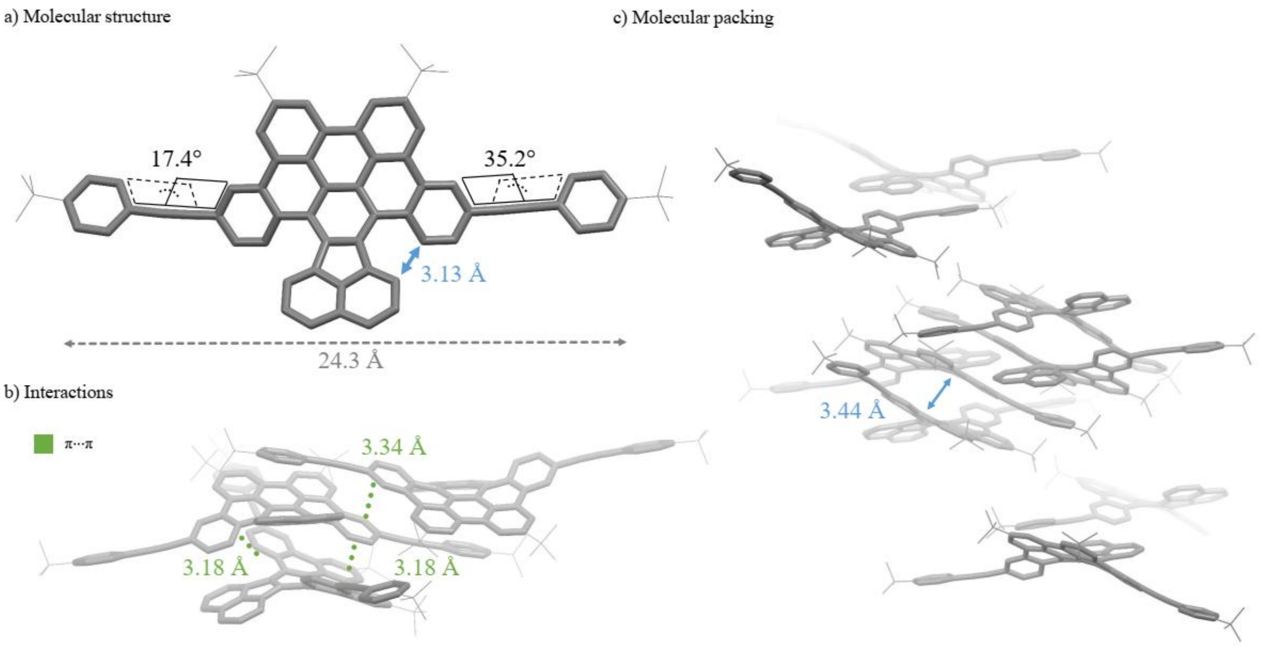


**Fig. S23** X-ray crystal structure of **6**. a) Front view. b) π···π interactions (green) between individual molecules of **6**. c) Virtual arrangement of the packing motif. Color code: carbon (grey). Hydrogen atoms and solvent molecules are omitted for clarity; *tert*-butyl groups are depicted as wire.

Compound **7** was crystallized from slow gas phase diffusion of MeOH into a saturated solution of the compound in toluene at rt. Species **7** crystallizes as yellow planks in the monoclinic space group *P2₁/n*.

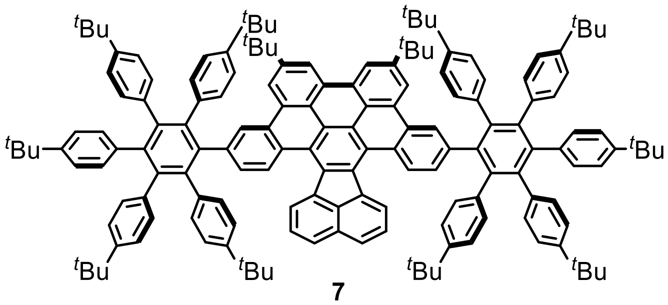


**7**

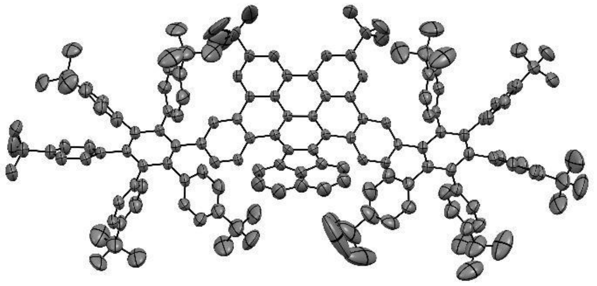

**Table S2.** Crystal data and structure refinement for **7** (ellipsoids at 50% probability).

| | | | |
|---|---|---|---|
| CCDC | | 2407549 | |
| Empirical Formula | | $C_{202}H_{212}$ | |
| Formula Weight | | 2639.70 | |
| Temperature / K | | 200(2) | |
| Wavelength / Å | | 1.54178 | |
| Crystal System | | monoclinic | |
| Space Group | | *P2₁/n* | |
| Z | | 4 | |
| *a* / Å | *α* / deg | 18.3531(8) | 90 |
| *b* / Å | *β* / deg | 34.8218(10) | 92.894(3) |
| *c* / Å | *γ* / deg | 29.2243(13) | 90 |
| Volume / $Å^3$ | | 18653.1(13) | |
| Density (Calculated) / $g/cm^3$ | | 0.94 | |
| Absorption Coefficient / $mm^{-1}$ | | 0.39 | |
| Crystal Shape (Color) | | plank (yellow) | |
| Crystal Size / $mm^3$ | | 0.600 × 0.110 × 0.056 | |
| Theta Range for Data Collection / deg | | 2.0 to 53.4 | |
| Index Ranges | | –19≤h≤19, –36≤k≤23, –30≤l≤30 | |
| Reflections Collected | | 91695 | |
| Reflections (Independent) | | 22012 (R(int) = 0.1103) | |
| Reflections (Observed) | | 13487 (I > 2σ(I)) | |
| Goodness-of-fit on $F^2$ | | 1.08 | |
| Final R Indices (I > 2sigma(I)) | | $R_1$ = 0.146, $wR_2$ = 0.344 | |
| Largest Diff. Peak and Hole / $eÅ^{-3}$ | | 0.72 and –0.37 | |

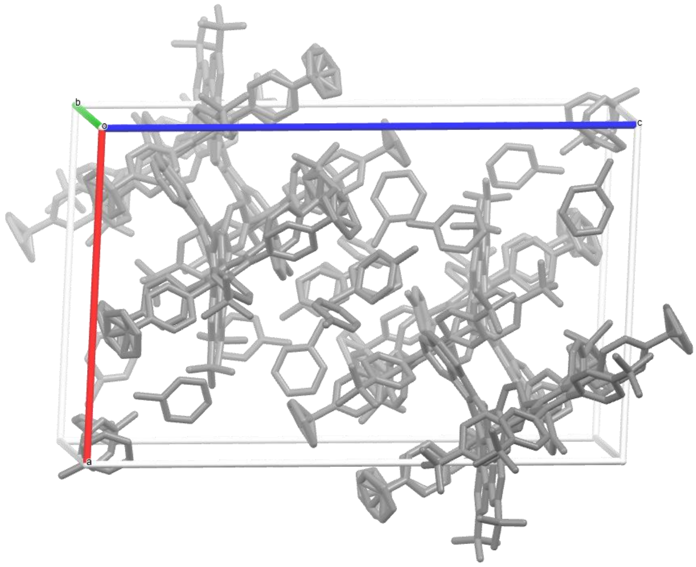


**Fig. S24** Unit cell of **7** along the b-axis. Hydrogen atoms omitted for clarity.

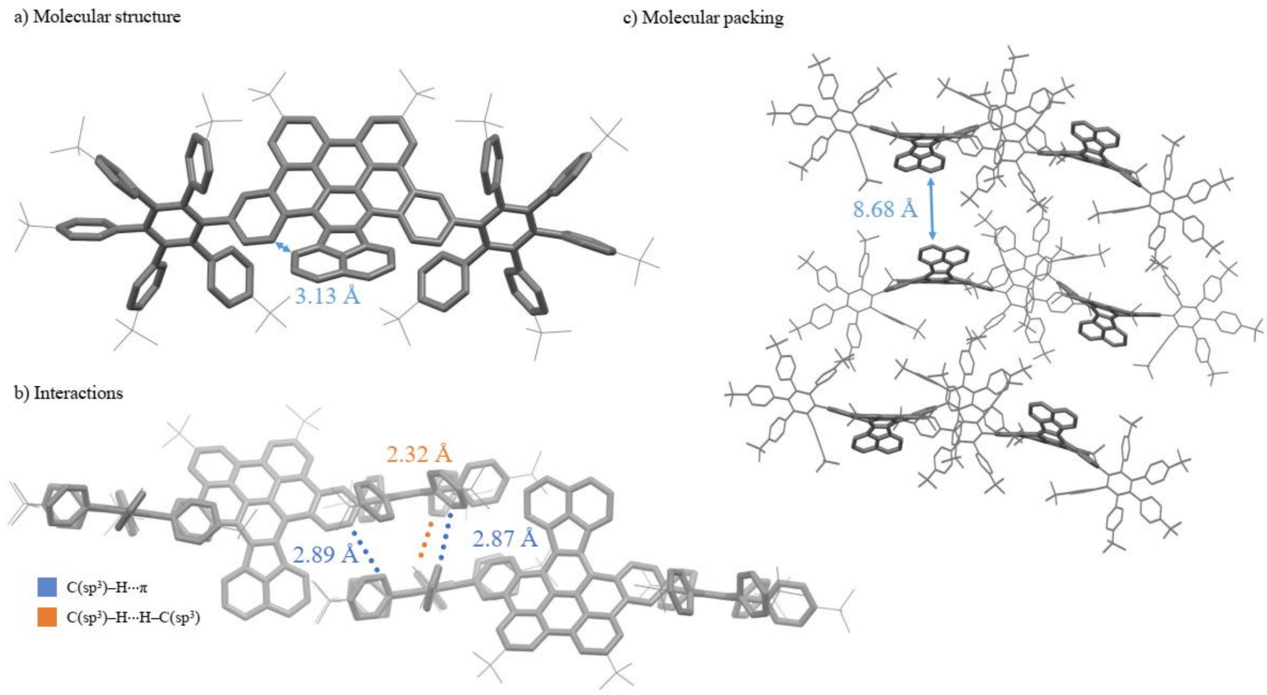


**Fig. S25** X-ray crystal structure of **7**. a) Front view. b) C(sp$^3$)–H···π (blue), C(sp$^3$)–H···H–C(sp$^3$) interactions (orange) between individual molecules of **7**. c) Virtual arrangement of the packing motif; lateral hexaphenylbenzene substituents are depicted as wire. Color code: carbon (grey). Hydrogen atoms and solvent molecules are omitted for clarity; *tert*-butyl groups are depicted as wire.

## 6. UV-vis absorption and fluorescence data

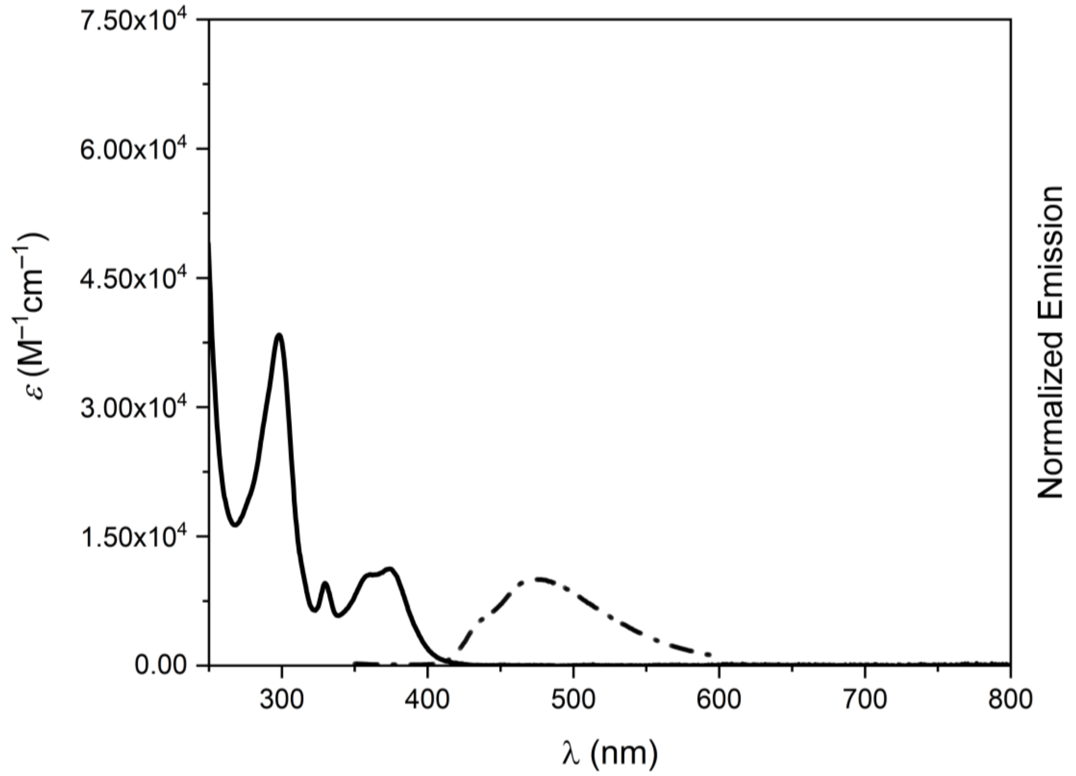


**Fig. S26** UV-Vis absorption and emission spectra of **4** in $CH_2Cl_2$.

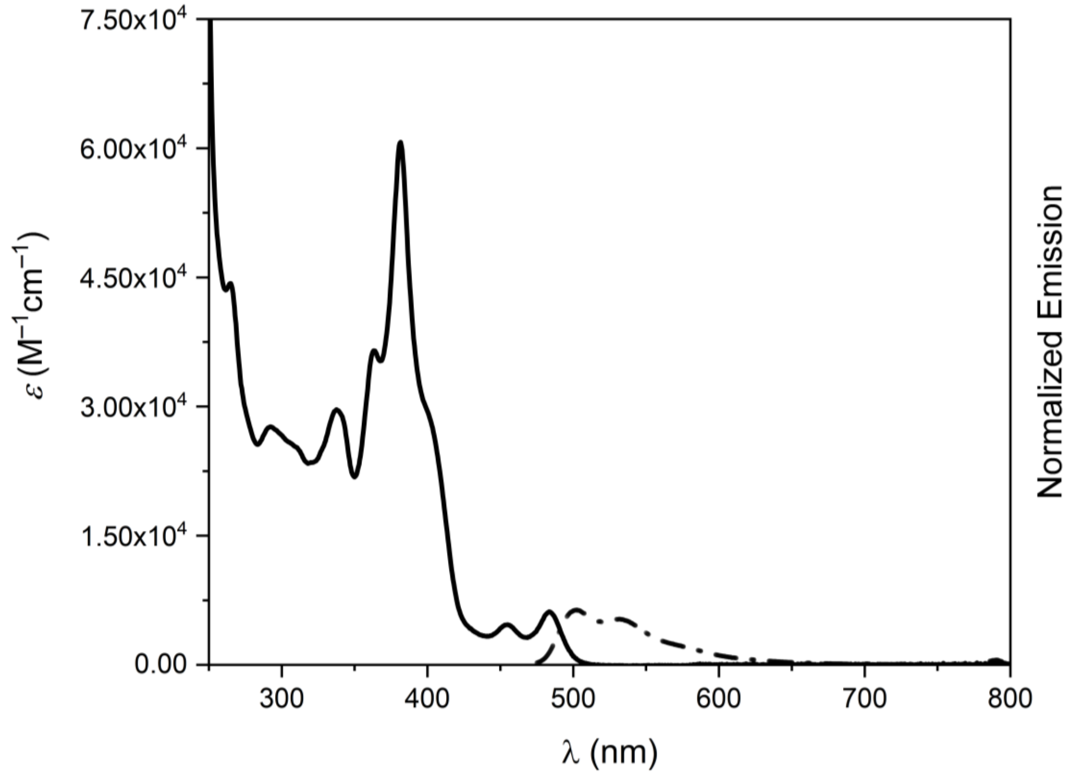


**Fig. S27** UV-Vis absorption and emission spectra of **5** in $CH_2Cl_2$.

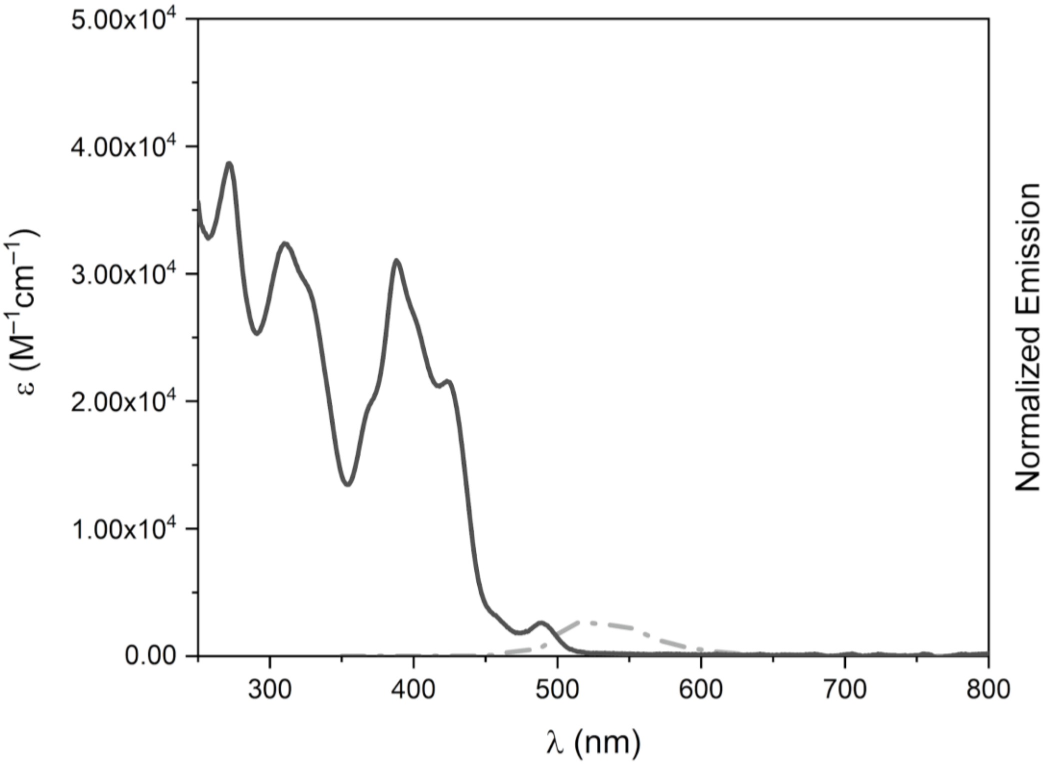


**Fig. S28** UV-Vis absorption and emission spectra of **6** in $CH_2Cl_2$.

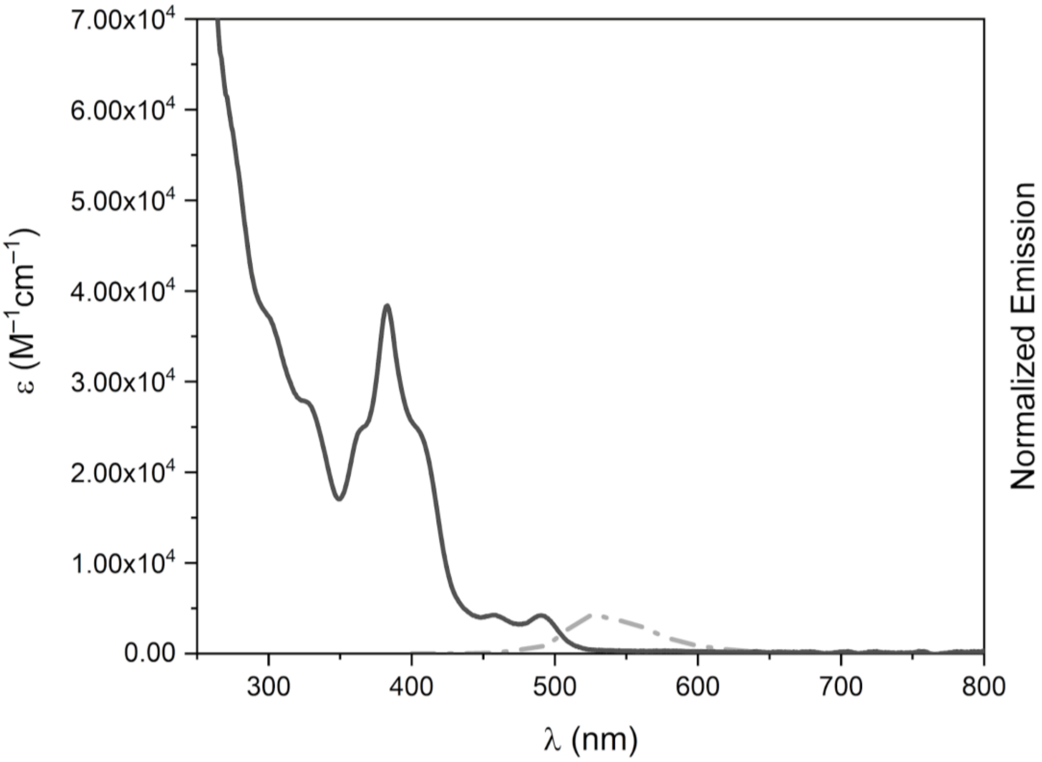


**Fig. S29** UV-Vis absorption and emission spectra of **7** in $CH_2Cl_2$.

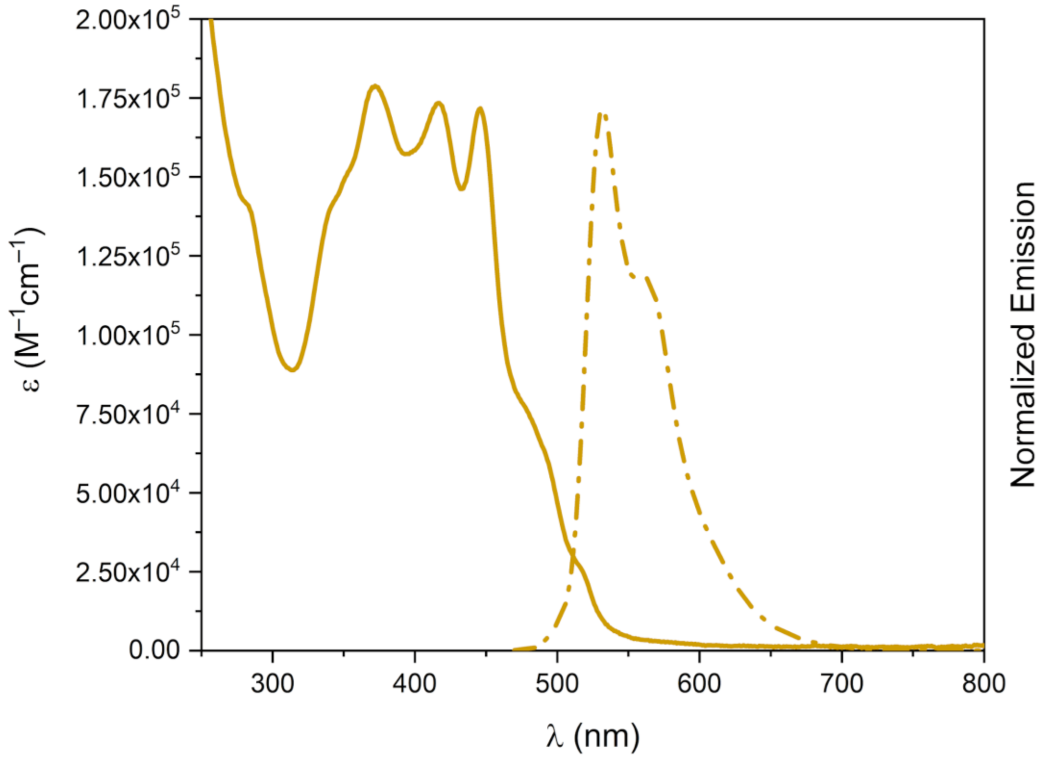


**Fig. S30** UV-Vis absorption and emission spectra of **1** in $CH_2Cl_2$.

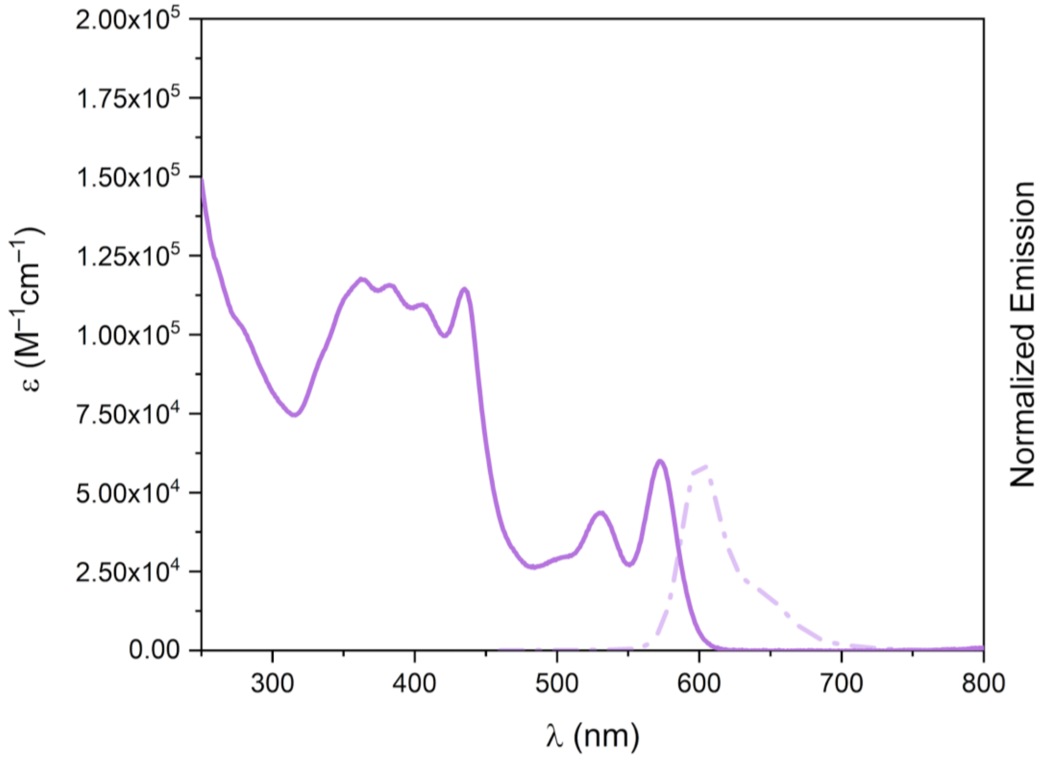


**Fig. S31** UV-Vis absorption and emission spectra of **2** in $CH_2Cl_2$.

## 7. Quantum yield determination and fluorescence lifetimes

The fluorescence quantum yields ($\phi_F$) were estimated using Coumarin 153 in dimethyl sulfoxide (DMSO) as standard ($\phi_F$ = 0.482, $\lambda_{em}$ = 500-600 nm). Several solutions of the compounds in DCM and the standard were prepared with concentration on the micromolar range. To avoid non-linear effects, the absorbances in the 5 mm quartz cuvettes were kept below 0.1 at the excitation wavelength (440 nm). The absorption and fluorescence spectrum were measured for each sample and standard using the same conditions. The slopes ($m$) obtained from the linear fit of integrated fluorescence intensity versus the absorbance (Fig. S30) were used to calculate the emission quantum yield according to eq. 2:

$$\phi_F = \phi_S \frac{m}{m_S}\frac{n^2}{n_S^2} \qquad \text{(eq. 2)}$$

where the subscript $s$ denotes the standard and $n$ the refractive index of the solvent.

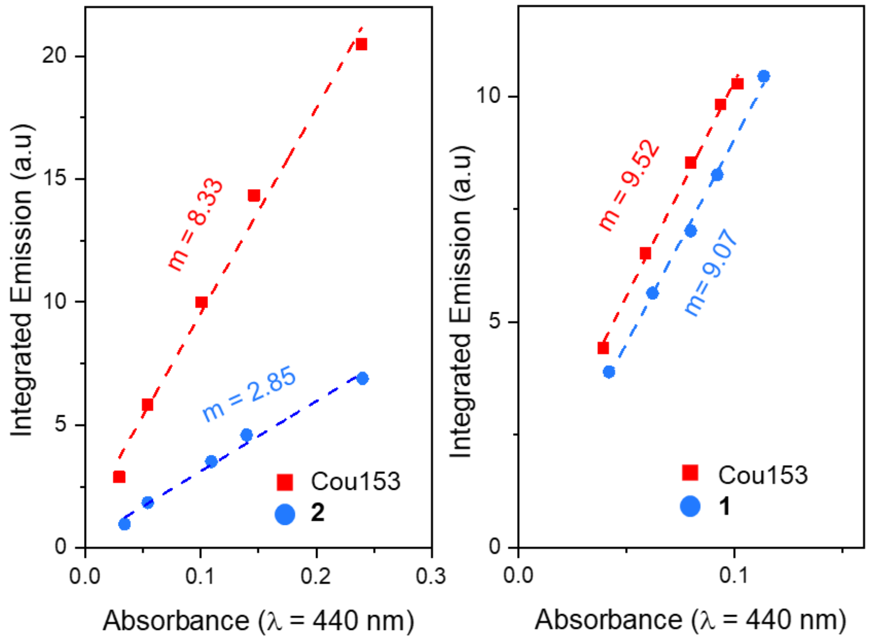


**Fig. S32** Integrated fluorescence intensity plotted as the function of the absorbance for compounds **1** and **2** and the standard (cou153). The linear fit is shown as a dashed line. The legend shows the slope.

The fluorescence emission decays recorded in DCM are shown in Fig. S31. The fluorescence lifetime is shorter in compound **2** ($\tau$ = 3.95 ns) than in compound 1 ($\tau$ =7.98 ns).

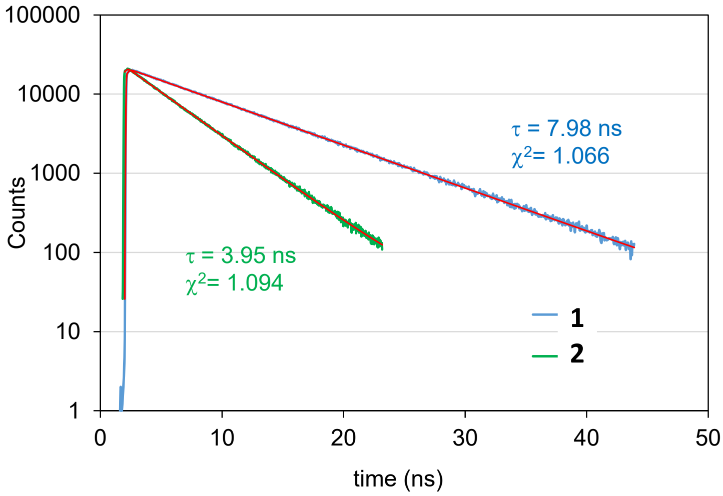


**Fig. S33** Fluorescence emission decay of compounds **1** (in blue) and **2** (in green) in dichloromethane. The monoexponential fitting of the decays are shown in red. The $\chi^2$ values close to unit are indicative of the good quality of the fittings.

## 8. Two-photon absorption

The two-photon absorption cross-section is shown on Figure S32. The two-photon absorption cross sections were estimated as an average between the values obtained using two different cross-section standards, Coumarin 153 in DMSO and Rhodamine 6G in methanol. The error bars shown in the spectra are mainly connected with the values obtained from the two standards. In general, the estimated cross section values are lower by 50% when using Coumarin 153.

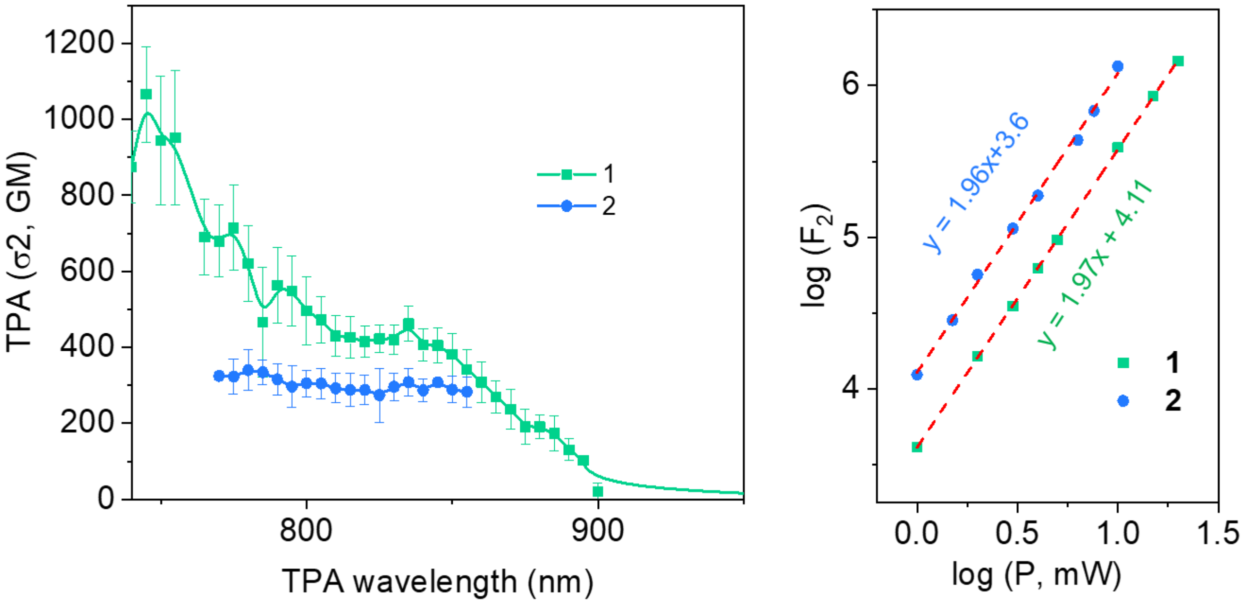


**Fig. S34** Two-photon absorption spectra of **1** and **2** (a) and the corresponding log-log plot of the two-photon emission as a function of the excitation power at 800 nm. A slope of ~2 is indicative of the quadratic dependence of the emission intensity on the excitation power.

## 9. Electrochemical data.

A BASi Cell Stand instrument with a glassy carbon disk working electrode (3.0 mm diameter), an Ag/AgCl (3 M NaCl) quasi-reference electrode, and a platinum wire auxiliary electrode were used to record cyclic voltammograms, differential pulse voltammograms and square wave voltammograms. Before each measurement, a 0.1 M electrolyte solution of *n*-$Bu_4NPF_6$ in anhydrous $CH_2Cl_2$ was degassed with nitrogen for 20 min. The respective compounds were measured either at a scan rate of 50 mV $s^{-1}$ or 149 mV $s^{-1}$, followed by the addition of ferrocene as the internal standard and re-measurement.

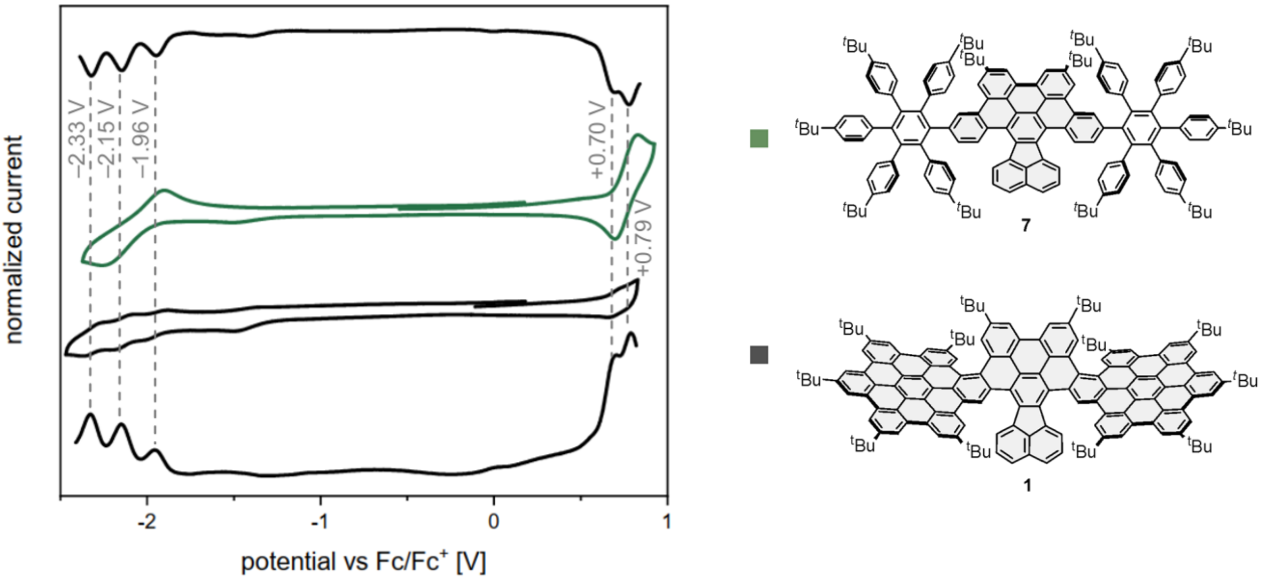


**Fig. S35** Electrochemical analysis of **1** (black) and **7** (green) at rt (~2 mM, vs Fc/Fc$^+$, THF, *n*-$Bu_4NPF_6$ as supporting electrolyte). Top: DPV of **1**, center: CV (scan rate 149 mV $s^{-1}$), bottom: SWV of **1**.

**10. Chiral stationary phase high performance liquid chromatography**

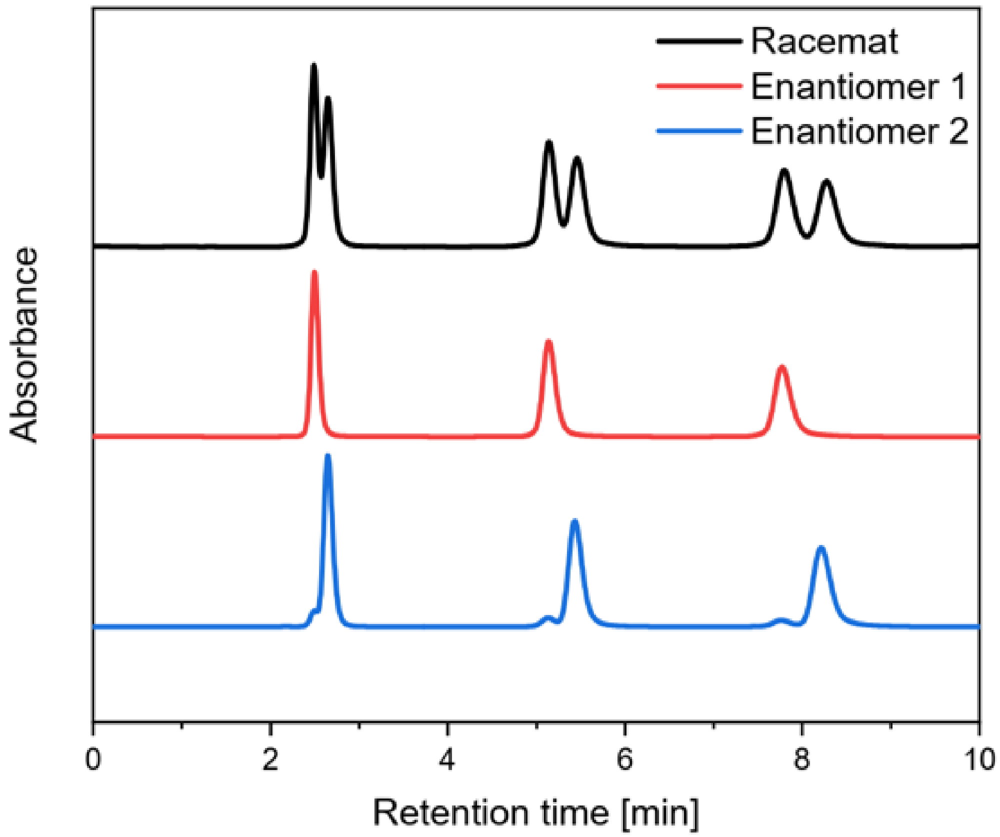


**Fig. S36** Chromatograms of a recycling run for the racemic resolution of compound **2** (black) and reinjection runs after collecting each chromatographic peak separately (red and blue). Enantiomer 2 %ee = 85%

## 11. Absorbance and luminescence dissymmetry factors

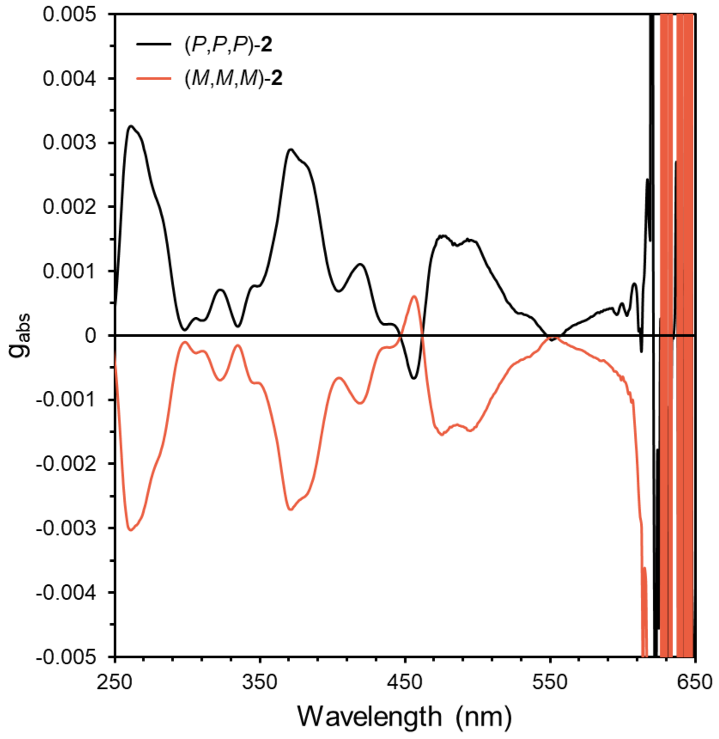


**Fig. S37** Absorbance dissymmetry factor spectra of enantiomers (*P*,*P*,*P*) and (*M*,*M*,*M*)-**2**

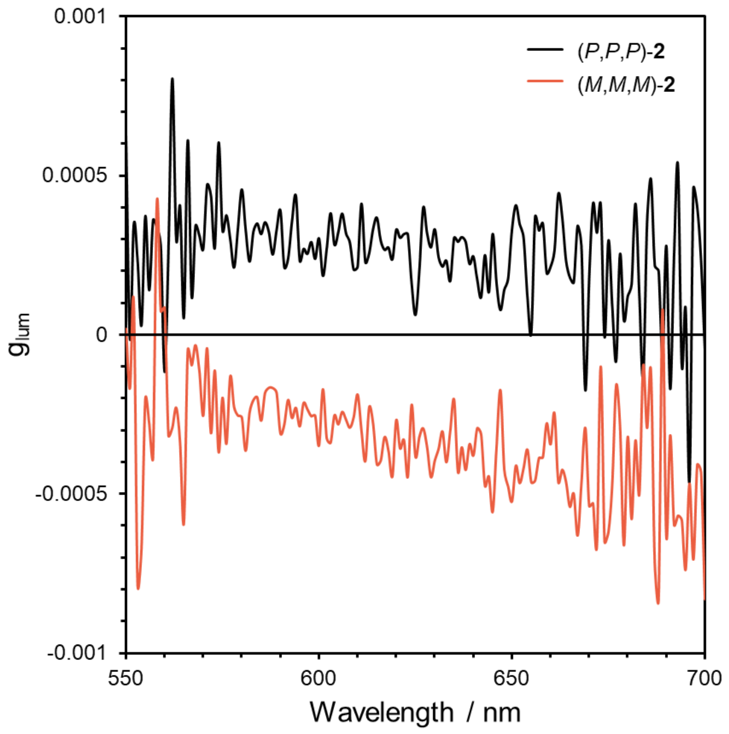


**Fig. S38** Luminescence dissymmetry factor spectra of enantiomers (*P*,*P*,*P*) and (*M*,*M*,*M*)-**2**

## 12. Geometrical aspects

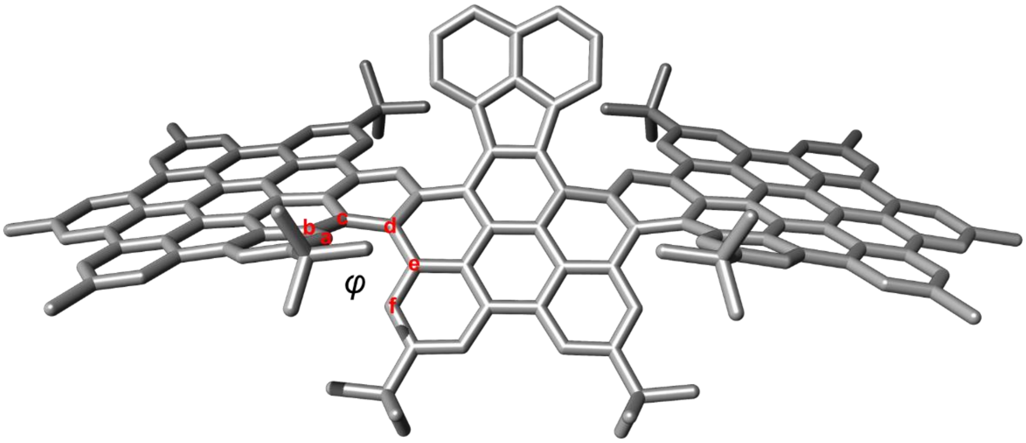


$\varphi = 24.13°$

**Fig. S39** Calculated torsion angle of the [5]helicene moiety in *meso*-**1**. Calculated as the average dihedral angle between carbons a–f.

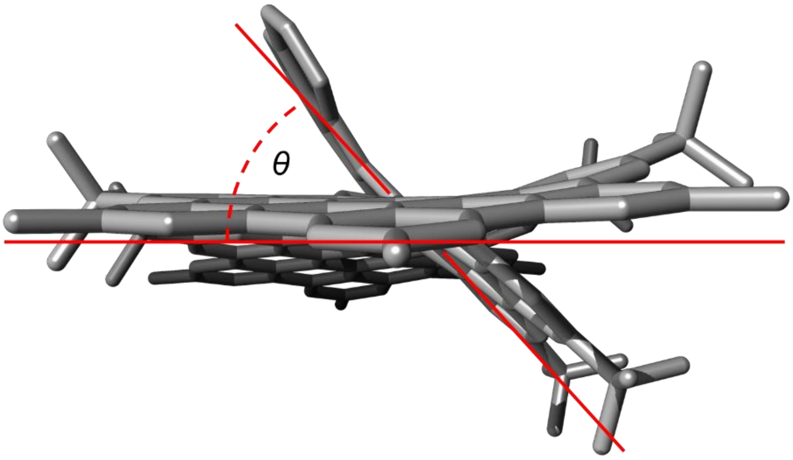


$\theta = 45.68°$

**Fig. S40** Side view of the optimized geometry of *meso*-**1**. Calculated bent angle between the lateral HBC unit and the central *seco*-HBC-like moiety in *meso*-**1**. Calculated from the planes of the edge benzenoid ring and the central benzenoid ring.

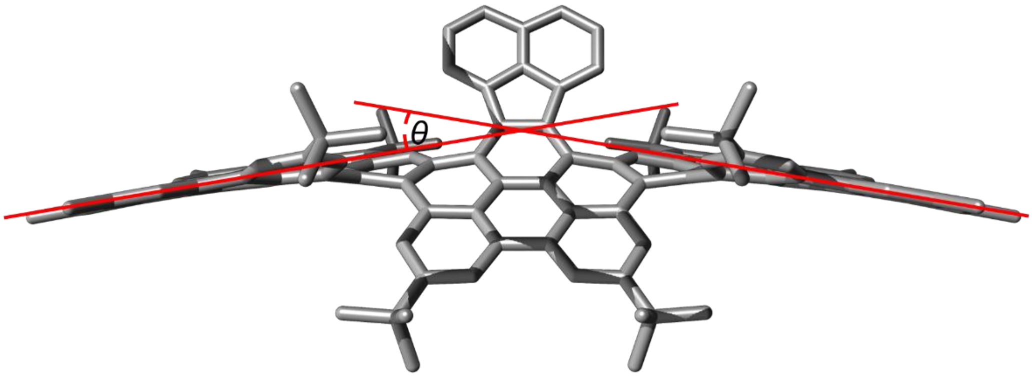


$\theta = 22.29°$

**Fig. S41** Front view of the optimized geometry of *meso*-**1**. Calculated bent angle between the lateral HBC units in *meso*-**1**. Calculated from the planes of the terminal benzenoid rings.

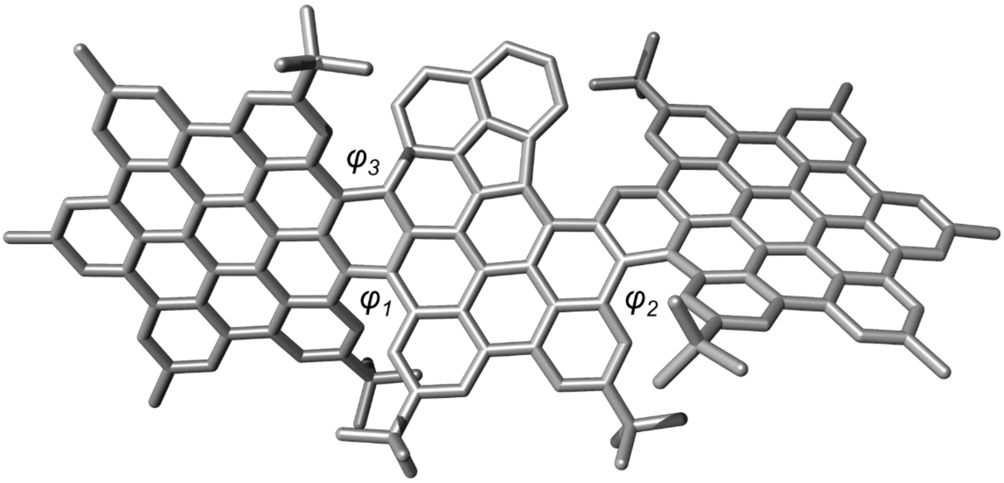


$\varphi_1 = 24.91°$ $\varphi_2 = 24.87°$ $\varphi_3 = 21.15°$

**Fig. S42** Calculated torsion angle of the [5]helicene moiety in (*M*,*M*,*M*)-**1**.

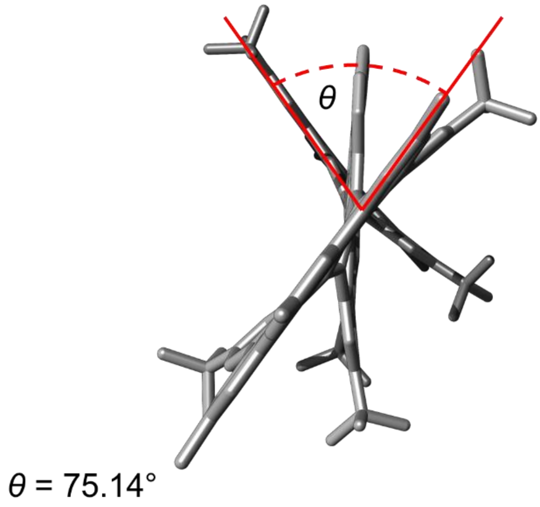


**Fig. S43** Side view of the optimized geometry of (*M*,*M*,*M*)-**2**. Calculated torsion angle between the terminal benzenoid rings.

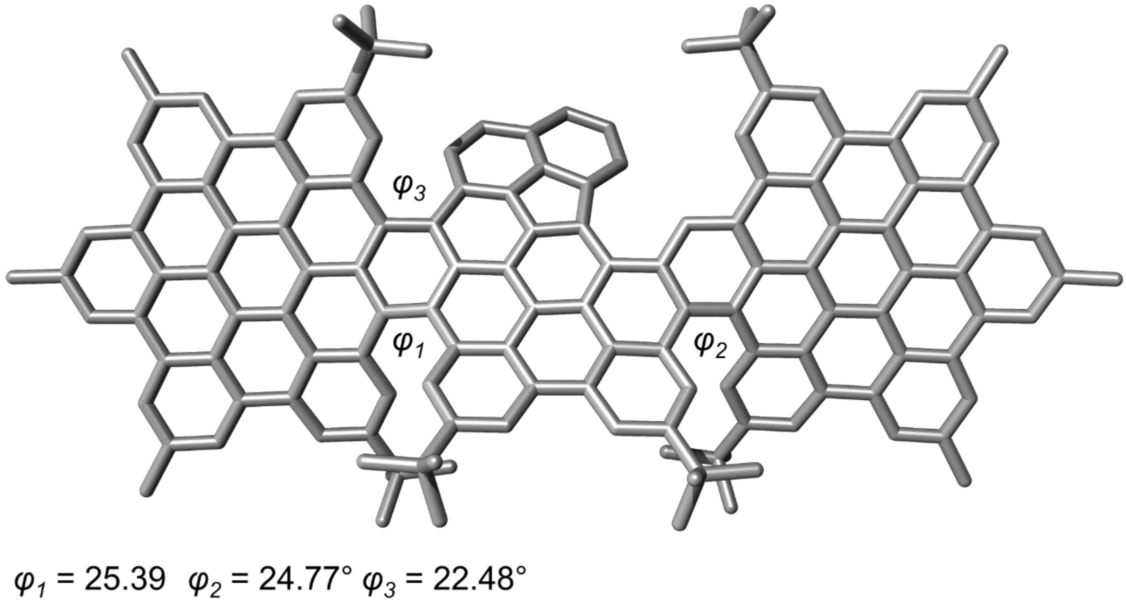


**Fig. S44** Calculated torsion angle of the [5]helicene moieties in (*P*,*M*,*M*)-**2**.

## 13. Calculated frontier molecular orbitals

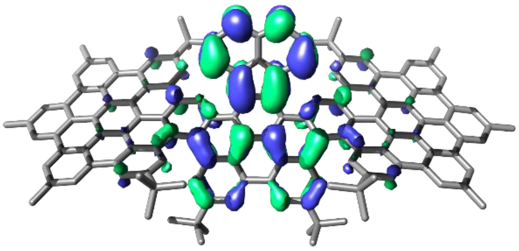

**LUMO**
**(-2.20 eV)**

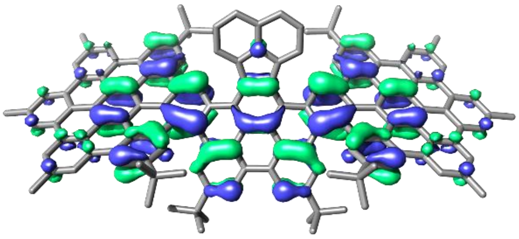

**HOMO**
**(-5.05 eV)**

(*P*, *M*)-**1**

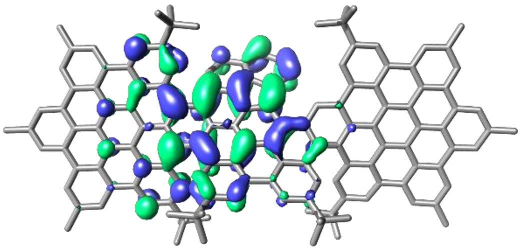

**LUMO**
**(-2.46 eV)**

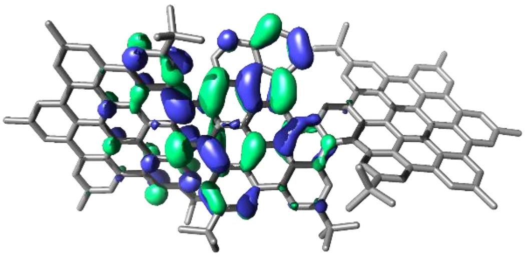

**LUMO**
**(-2.47 eV)**

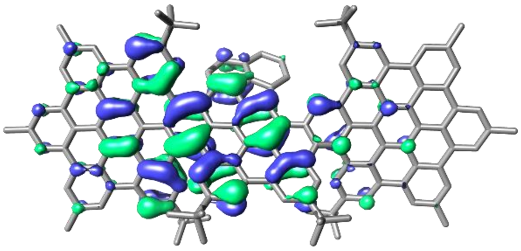

**HOMO**
**(-4.99 eV)**

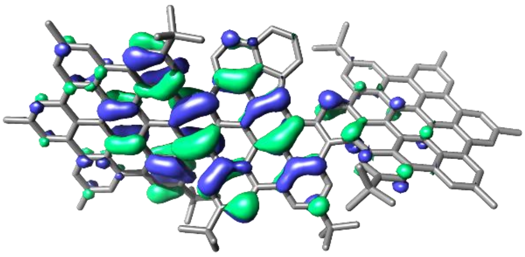

**HOMO**
**(-4.97 eV)**

(*P*, *M*, *M*)-**2**

(*M*, *M*, *M*)-**2**

**Fig. S45** Calculated frontier molecular orbitals for compounds (*P*,*M*)-**1**, (*M*,*M*,*M*)-**2** and (*P*,*M*,*M*)-**2**.

## 14. Theoretical UV-vis absorption and ECD data

**Table S3.** Calculated 50 lowest electronic transitions for compound (*P*,*M*)-**1**, their energies (λ, in nm) and oscillator strength (*f*, in cgs units). Correction: –0.4 eV.

| Transition number | λ | *f* | Transition number | λ | *f* |
|---|---|---|---|---|---|
| 1 | 490.12 | 0.8911 | 26 | 333.57 | 0.3926 |
| 2 | 489.42 | 0.0585 | 27 | 333.45 | 0.0026 |
| 3 | 470.08 | 0.0329 | 28 | 329.60 | 0.0594 |
| 4 | 468.43 | 0.0228 | 29 | 327.26 | 0.0678 |
| 5 | 446.37 | 0.1514 | 30 | 324.85 | 0.0793 |
| 6 | 435.57 | 0.5157 | 31 | 317.85 | 0.0583 |
| 7 | 427.47 | 2.4574 | 32 | 314.73 | 0.0159 |
| 8 | 404.86 | 0.1054 | 33 | 313.65 | 0.0036 |
| 9 | 397.42 | 0.0303 | 34 | 313.53 | 0.1532 |
| 10 | 390.97 | 0.6180 | 35 | 312.06 | 0.0176 |
| 11 | 383.80 | 1.4888 | 36 | 310.15 | 0.0151 |
| 12 | 383.55 | 0.3447 | 37 | 308.72 | 0.0117 |
| 13 | 380.59 | 0.1580 | 38 | 308.66 | 0.1986 |
| 14 | 379.36 | 0.0004 | 39 | 306.97 | 0.0065 |
| 15 | 362.76 | 0.0070 | 40 | 306.36 | 0.0011 |
| 16 | 362.14 | 0.0271 | 41 | 304.42 | 0.1214 |
| 17 | 359.78 | 0.0491 | 42 | 304.29 | 0.1830 |
| 18 | 359.66 | 0.2952 | 43 | 301.04 | 0.0035 |
| 19 | 356.60 | 0.4315 | 44 | 300.13 | 0.0208 |
| 20 | 353.02 | 0.0044 | 45 | 297.63 | 0.0149 |
| 21 | 351.97 | 0.7792 | 46 | 297.46 | 0.0052 |
| 22 | 351.24 | 0.8200 | 47 | 294.38 | 0.0138 |
| 23 | 344.95 | 0.5229 | 48 | 294.33 | 0.0513 |
| 24 | 335.75 | 0.0004 | 49 | 293.58 | 0.0693 |
| 25 | 334.94 | 0.0556 | 50 | 292.46 | 0.0187 |

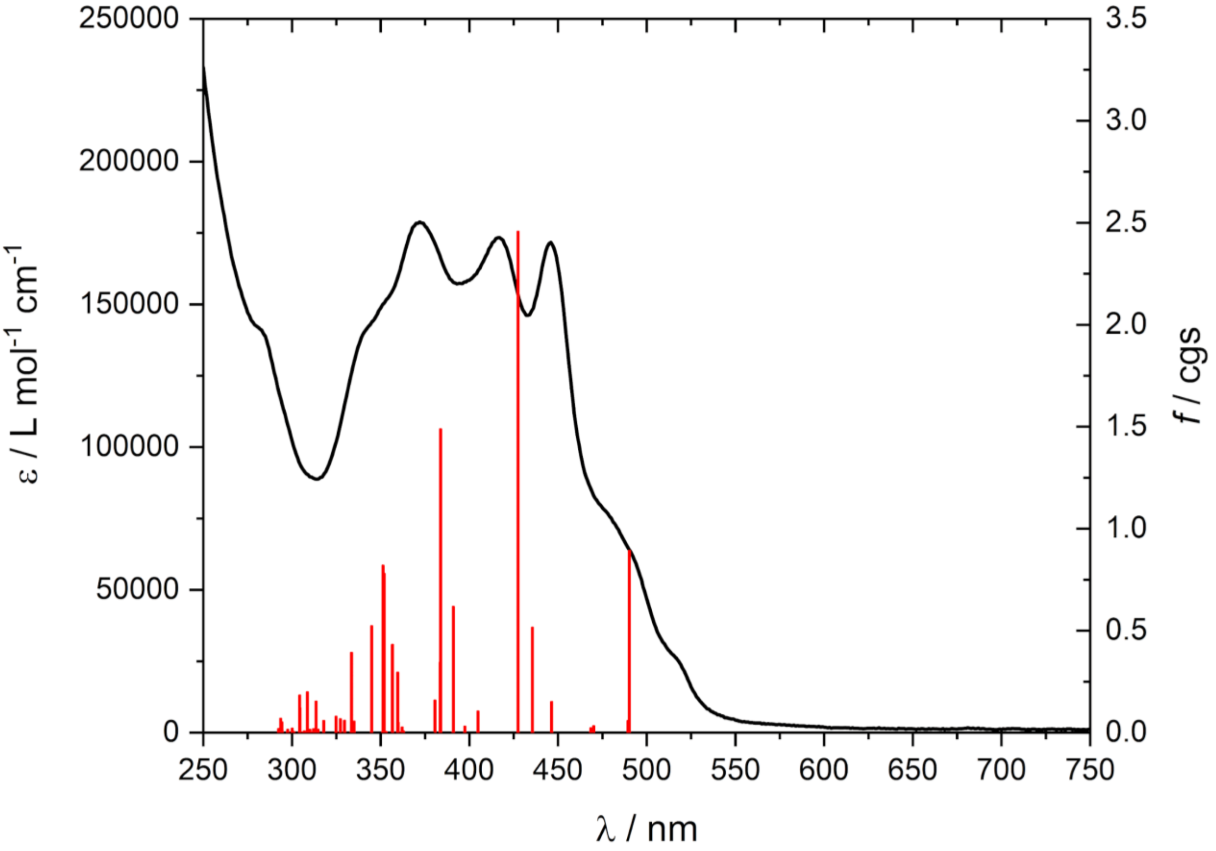


**Fig. S46** Experimental UV-Vis spectrum of compound **1** in dichloromethane and calculated oscillatory strength (*f*) of the 50 lowest energetic electronic transitions of (*P*,*M*)-**1**.

**Table S4.** Calculated 50 lowest electronic transitions for compound (*P*,*M*,*M*)-**2**, their energies (λ, in nm), oscillator strength (*f*, in cgs units) and rotatory strength (*R*, $10^{-40}$ $esu^2$ $cm^2$).

| Transition number | λ | *f* | *R* | Transition number | λ | *f* | *R* |
|---|---|---|---|---|---|---|---|
| 1 | 571.64 | 1.1803 | -7.2125 | 26 | 337.27 | 0.1235 | -144.5781 |
| 2 | 512.70 | 0.0894 | 64.8434 | 27 | 333.01 | 0.1239 | -22.5846 |
| 3 | 482.83 | 0.0838 | -22.5932 | 28 | 329.97 | 0.0661 | 57.0335 |
| 4 | 469.48 | 0.0429 | -96.1869 | 29 | 327.93 | 0.0801 | 0.9205 |
| 5 | 452.89 | 0.5676 | -122.7432 | 30 | 327.16 | 0.1126 | -218.7114 |
| 6 | 444.07 | 0.0338 | 84.5349 | 31 | 322.00 | 0.1809 | -100.832 |
| 7 | 417.02 | 1.8882 | -35.6685 | 32 | 317.84 | 0.0258 | -1.1171 |
| 8 | 414.56 | 0.4373 | 256.6748 | 33 | 315.30 | 0.1220 | -98.3711 |
| 9 | 406.29 | 0.1208 | -47.1007 | 34 | 313.96 | 0.0087 | 42.9276 |
| 10 | 395.89 | 0.3299 | -465.6631 | 35 | 313.66 | 0.0143 | -2.6222 |
| 11 | 392.21 | 0.2921 | 448.6436 | 36 | 310.99 | 0.1330 | 34.0334 |
| 12 | 389.88 | 0.2416 | -23.7475 | 37 | 309.22 | 0.0856 | 42.9372 |
| 13 | 386.56 | 0.5767 | -109.1148 | 38 | 307.81 | 0.0335 | 17.6799 |
| 14 | 379.95 | 0.3186 | 16.558 | 39 | 305.60 | 0.1804 | 184.5473 |
| 15 | 377.89 | 0.498 | 47.4141 | 40 | 305.36 | 0.0530 | 72.3886 |
| 16 | 374.91 | 0.4713 | 668.5559 | 41 | 304.55 | 0.0457 | 3.4605 |
| 17 | 364.22 | 0.0596 | -195.0922 | 42 | 303.75 | 0.0388 | -16.7004 |
| 18 | 361.40 | 0.1104 | 2.9956 | 43 | 302.50 | 0.1523 | 43.9084 |
| 19 | 360.07 | 0.0386 | -47.5799 | 44 | 301.30 | 0.1745 | 179.6492 |
| 20 | 356.26 | 0.4038 | -478.7486 | 45 | 300.92 | 0.0062 | -17.6866 |
| 21 | 353.13 | 0.5101 | 49.9219 | 46 | 298.51 | 0.0226 | -63.338 |
| 22 | 349.79 | 0.5935 | 88.1571 | 47 | 297.21 | 0.0319 | 86.6765 |
| 23 | 347.83 | 0.2063 | -277.4857 | 48 | 296.47 | 0.0507 | -56.667 |
| 24 | 345.88 | 0.2343 | 252.4719 | 49 | 295.90 | 0.0459 | 38.5018 |
| 25 | 339.70 | 0.3737 | 157.0906 | 50 | 293.57 | 0.0474 | -69.5676 |

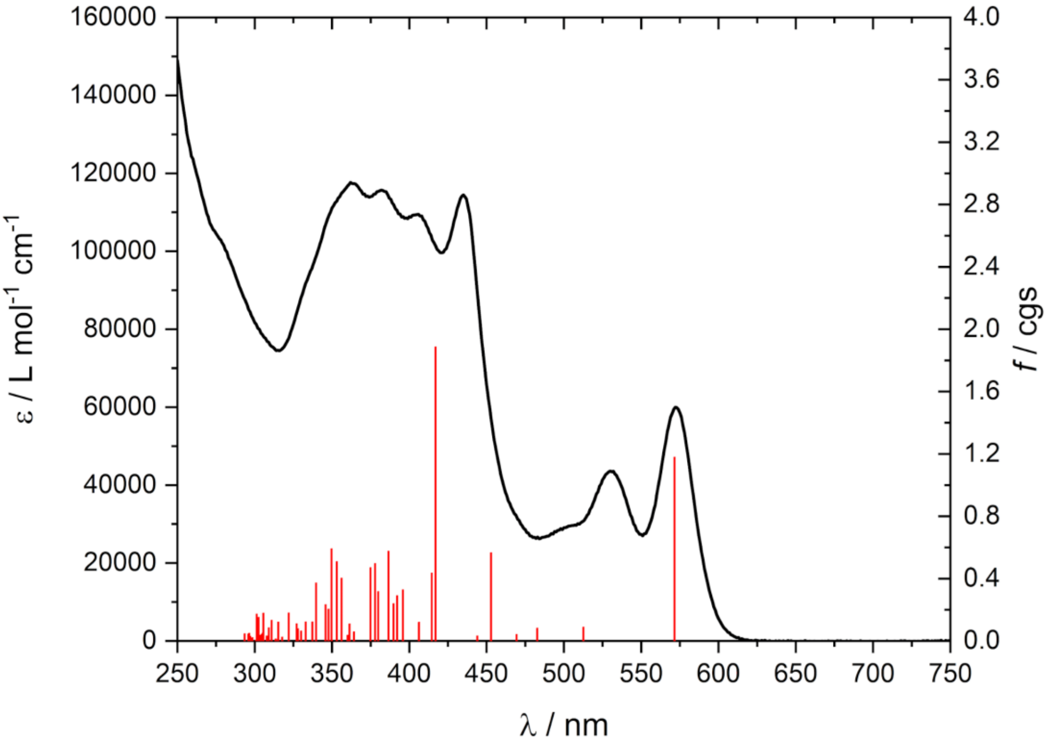


**Fig. S47** Experimental UV-Vis spectrum of compound (*P*,*M*,*M*)-**2** in $CH_2Cl_2$ and calculated oscillator strength (*f*) of the 50 lowest energetic electronic transitions of (*P*,*M*,*M*)-**2**. Correction: -0.4 eV.

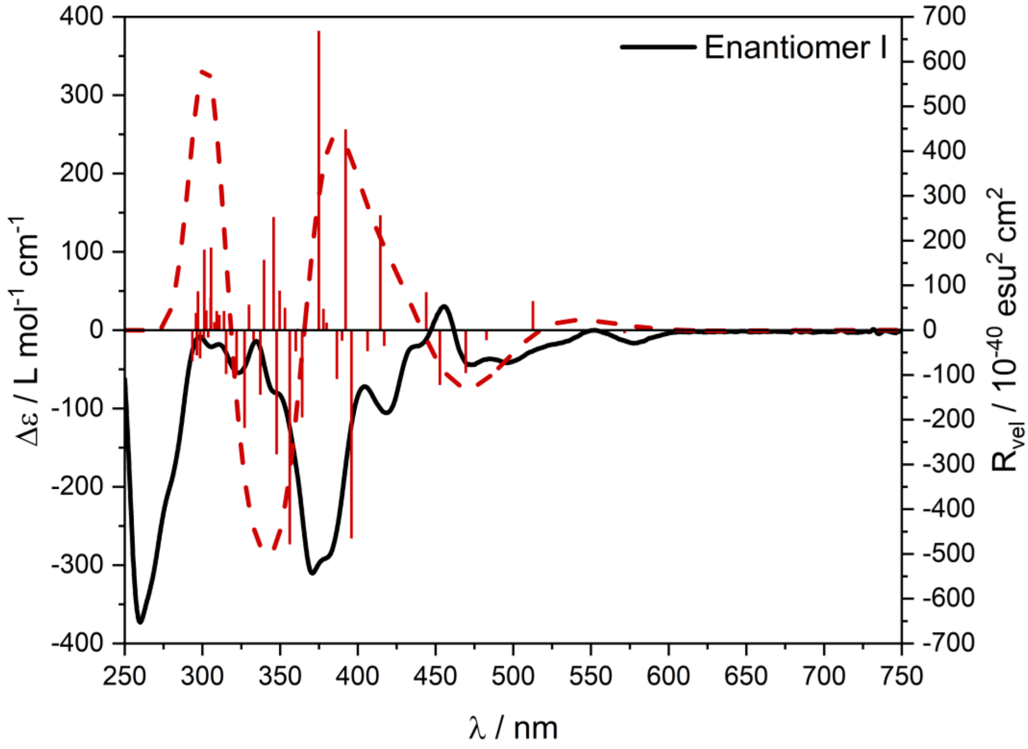


**Fig. S48** Experimental ECD spectrum of compound **2** in $CH_2Cl_2$ and calculated rotatory strength (R) of the 50 lowest energetic electronic transitions for (*P*,*M*,*M*)-**2**. Correction: –0.40 eV.

**Table S5.** Calculated 50 lowest electronic transitions for compound (*M*,*M*,*M*)-**2**, their energies (in nm), oscillator strength (in cgs units) and rotatory strength.

| Transition number | λ | *f* | *R* | Transition number | λ | *f* | *R* |
|---|---|---|---|---|---|---|---|
| 1 | 579.82 | 1.1793 | -43.0663 | 26 | 338.66 | 0.0604 | 0.7902 |
| 2 | 515.38 | 0.0925 | 17.4593 | 27 | 333.04 | 0.0873 | 90.7939 |
| 3 | 482.57 | 0.0842 | -126.0828 | 28 | 330.16 | 0.1522 | 273.637 |
| 4 | 469.88 | 0.0373 | -105.2802 | 29 | 328.51 | 0.0301 | 121.2138 |
| 5 | 452.29 | 0.5003 | -121.1282 | 30 | 327.56 | 0.0905 | -13.6443 |
| 6 | 445.31 | 0.0135 | 9.9886 | 31 | 322.21 | 0.1888 | 119.1735 |
| 7 | 418.12 | 1.8676 | 61.5676 | 32 | 318.30 | 0.0346 | -65.1538 |
| 8 | 415.94 | 0.4517 | 171.0132 | 33 | 316.16 | 0.0968 | 245.0746 |
| 9 | 407.15 | 0.0833 | -33.0578 | 34 | 315.01 | 0.0155 | -52.498 |
| 10 | 398.25 | 0.2940 | 15.5522 | 35 | 314.51 | 0.0495 | -197.6249 |
| 11 | 392.23 | 0.3783 | 20.9386 | 36 | 311.39 | 0.1041 | 19.6208 |
| 12 | 391.16 | 0.3618 | 24.9864 | 37 | 310.37 | 0.0833 | -9.7295 |
| 13 | 388.53 | 0.3920 | -43.9226 | 38 | 308.25 | 0.0499 | 88.4051 |
| 14 | 380.47 | 0.3129 | 234.1635 | 39 | 306.17 | 0.2162 | 50.9757 |
| 15 | 379.48 | 0.5167 | 525.1058 | 40 | 305.69 | 0.0010 | 0.4178 |
| 16 | 375.79 | 0.6335 | -1967.8031 | 41 | 304.98 | 0.0139 | -0.5484 |
| 17 | 364.71 | 0.1506 | -328.5814 | 42 | 303.77 | 0.0831 | 44.893 |
| 18 | 361.82 | 0.0370 | 240.4815 | 43 | 302.35 | 0.1127 | 1.4545 |
| 19 | 361.34 | 0.1336 | 68.1866 | 44 | 301.93 | 0.1240 | -41.7921 |
| 20 | 357.44 | 0.0900 | 1003.8491 | 45 | 301.37 | 0.0723 | -88.6044 |
| 21 | 354.09 | 0.3456 | 1012.0178 | 46 | 299.00 | 0.0261 | -22.3816 |
| 22 | 350.70 | 0.7545 | -1752.3459 | 47 | 297.90 | 0.0004 | -6.8788 |
| 23 | 348.61 | 0.3444 | -1225.9253 | 48 | 297.10 | 0.0961 | -18.6249 |
| 24 | 346.62 | 0.3313 | -550.3729 | 49 | 296.22 | 0.0290 | 17.6289 |
| 25 | 340.30 | 0.3883 | 722.0401 | 50 | 293.70 | 0.0317 | 19.6382 |

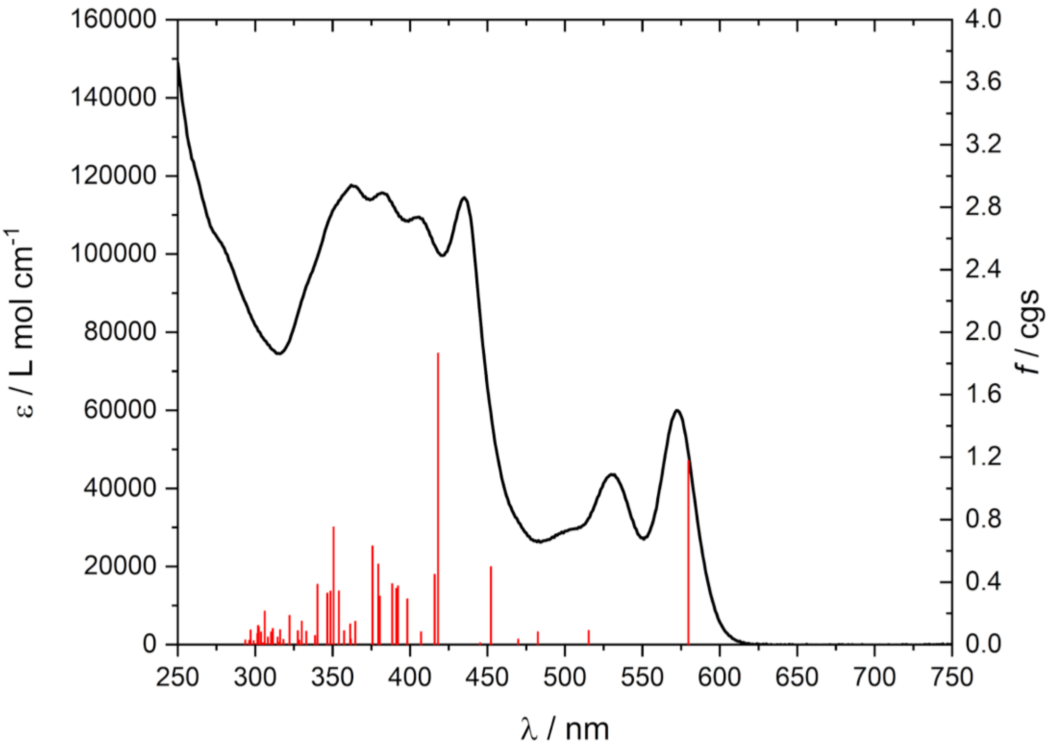


**Fig. S49** Experimental UV-Vis spectrum of compound **2** in $CH_2Cl_2$ and calculated oscillator strength (*f*) of the 50 lowest energetic electronic transitions of (*M*,*M*,*M*)-**2**. Correction: -0.4 eV.

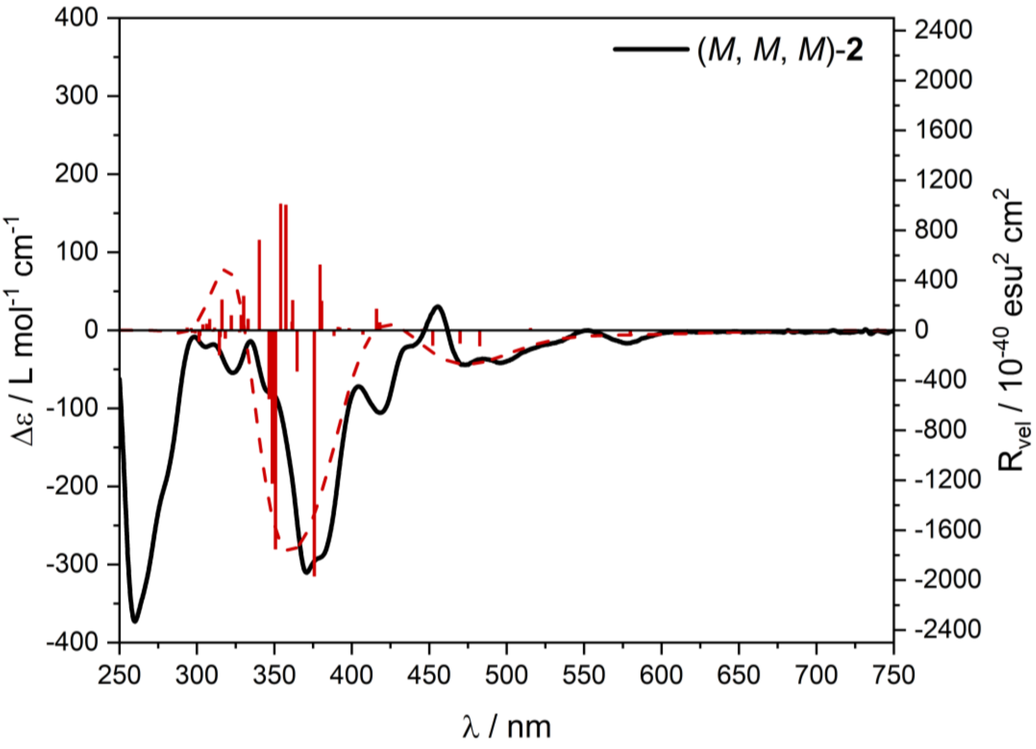


**Fig. S50** Experimental ECD spectrum of compound **2** in $CH_2Cl_2$ and calculated rotatory strength (R) of the 50 lowest energetic electronic transitions for (*M*,*M*,*M*)-**2**. Correction: –0.4 eV.

**15. Harmonic oscillator model of aromaticity (HOMA) and nucleus independent chemical shift (NICS)**

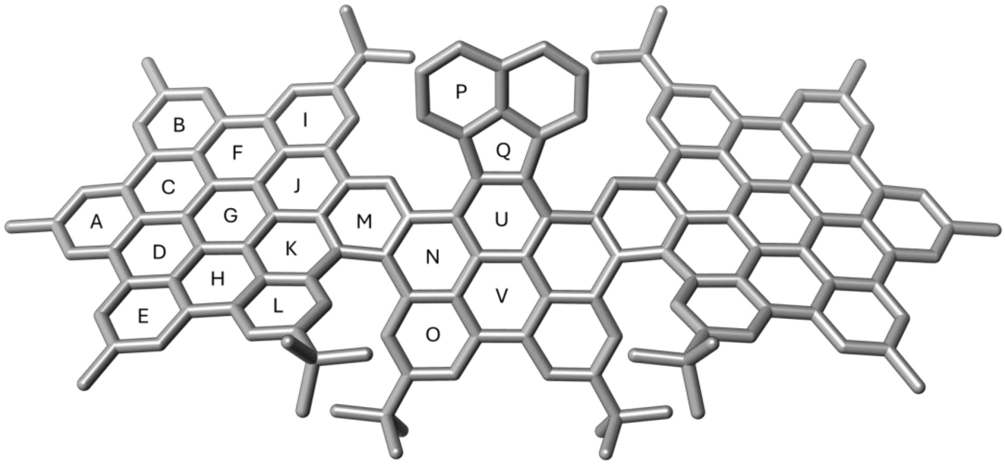


| Ring | HOMA | $NICS(0)_{iso}$ | Ring | HOMA | $NICS(0)_{iso}$ |
|---|---|---|---|---|---|
| A | 0.97 | -9.4041 | K | 0.84 | 0.8864 |
| B | 0.97 | -9.4783 | L | 0.98 | -8.6415 |
| C | 0.82 | 1.0925 | M | 0.94 | -5.9465 |
| D | 0.82 | 0.8525 | N | 0.78 | 0.9934 |
| E | 0.97 | -9.2066 | O | 0.97 | -8.3648 |
| F | 0.81 | 1.0582 | P | 0.96 | -6.9254 |
| G | 0.94 | -12.3775 | Q | 0.65 | 5.5777 |
| H | 0.82 | 1.9431 | U | 0.95 | -7.0456 |
| I | 0.98 | -8.8775 | V | 0.80 | 1.2261 |
| J | 0.82 | 2.1857 | | | |

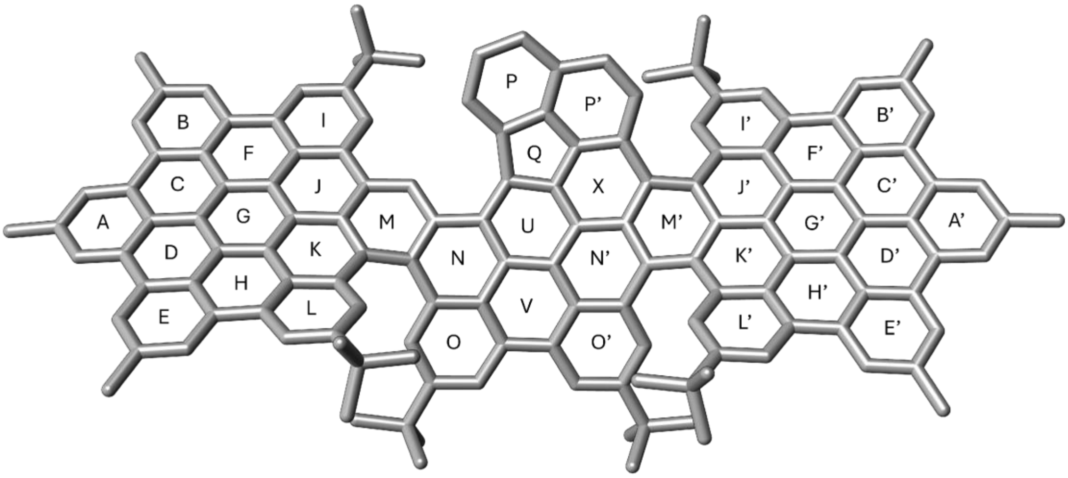


| Ring | HOMA | NICS(0)$_{iso}$ | Ring | HOMA | NICS(0)$_{iso}$ |
|---|---|---|---|---|---|
| A | 0.97 | -9.4528 | A´ | 0.97 | -9.4406 |
| B | 0.97 | -9.3685 | B´ | 0.97 | -9.2840 |
| C | 0.82 | 1.1225 | C´ | 0.81 | 1.0387 |
| D | 0.82 | 0.8340 | D´ | 0.81 | 1.0303 |
| E | 0.97 | -9.2806 | E´ | 0.98 | -9.3128 |
| F | 0.81 | 1.1006 | F´ | 0.82 | 1.6420 |
| G | 0.94 | -12.3975 | G´ | 0.94 | -12.3506 |
| H | 0.82 | 1.9817 | H´ | 0.82 | 1.8547 |
| I | 0.98 | -8.7659 | I´ | 0.98 | -8.6226 |
| J | 0.82 | 2.2007 | J´ | 0.83 | 1.4167 |
| K | 0.84 | 0.9762 | K´ | 0.82 | 1.3458 |
| L | 0.97 | -8.6647 | L´ | 0.98 | -8.6048 |
| M | 0.94 | -6.2797 | M´ | 0.90 | -7.0005 |
| N | 0.76 | 1.2821 | N´ | 0.81 | 2.1822 |
| O | 0.97 | -8.4411 | O´ | 0.97 | -8.1950 |
| P | 0.96 | -6.6499 | P´ | 0.82 | -5.3454 |
| Q | 0.69 | 7.7852 | X | 0.82 | -1.0444 |
| U | 0.93 | -8.5432 | | | |
| V | 0.81 | 1.5739 | | | |

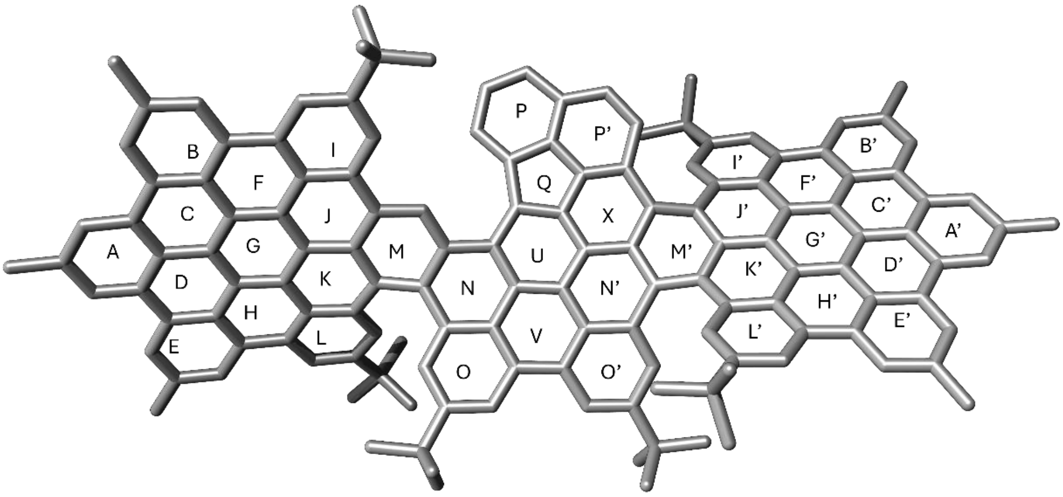


| Ring | HOMA | NICS(0)iso | Ring | HOMA | NICS(0)iso |
|---|---|---|---|---|---|
| A | 0.97 | -9.4820 | A´ | 0.97 | -9.4655 |
| B | 0.93 | -9.4687 | B´ | 0.97 | -9.2971 |
| C | 0.82 | 1.0216 | C´ | 0.81 | 1.0658 |
| D | 0.82 | 0.9760 | D´ | 0.81 | 0.9792 |
| E | 0.97 | -9.2896 | E´ | 0.97 | -9.3212 |
| F | 0.81 | 1.1595 | F´ | 0.82 | 1.5626 |
| G | 0.95 | -12.4010 | G´ | 0.94 | -12.3190 |
| H | 0.83 | 1.5047 | H´ | 0.82 | 1.8134 |
| I | 0.98 | -8.8473 | I´ | 0.98 | -8.5730 |
| J | 0.82 | 2.0625 | J´ | 0.83 | 1.4366 |
| K | 0.83 | 1.3896 | K´ | 0.82 | 1.2295 |
| L | 0.98 | -8.7566 | L´ | 0.98 | -8.6588 |
| M | 0.95 | -6.1022 | M´ | 0.90 | -6.8372 |
| N | 0.75 | 1.3645 | N´ | 0.82 | 2.1030 |
| O | 0.96 | -8.3919 | O´ | 0.97 | -8.2767 |
| P | 0.95 | -6.5866 | P´ | 0.92 | -5.2067 |
| Q | 0.69 | 7.8411 | X | 0.81 | -1.3644 |
| U | 0.93 | -8.4308 | | | |
| V | 0.82 | 1.4987 | | | |

**16. Anisotropy of the induced current density (ACID)**

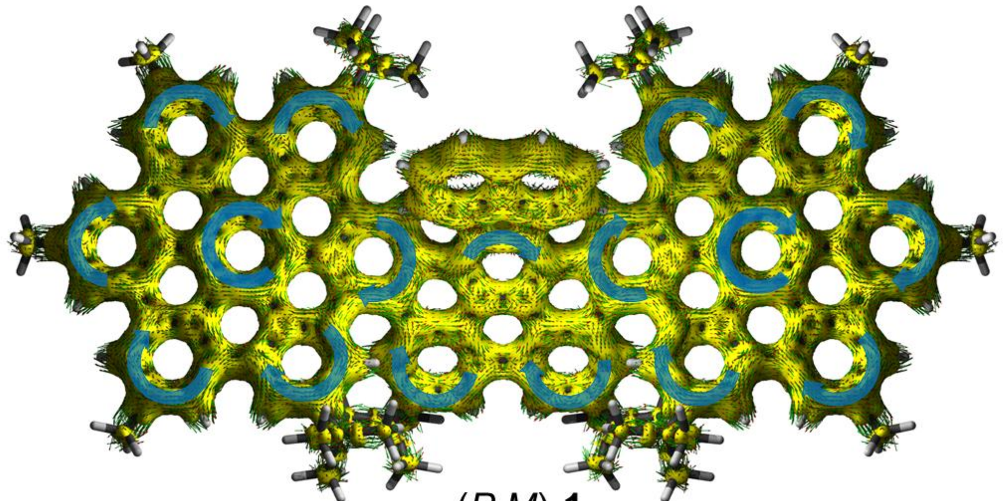

(*P*,*M*)-**1**

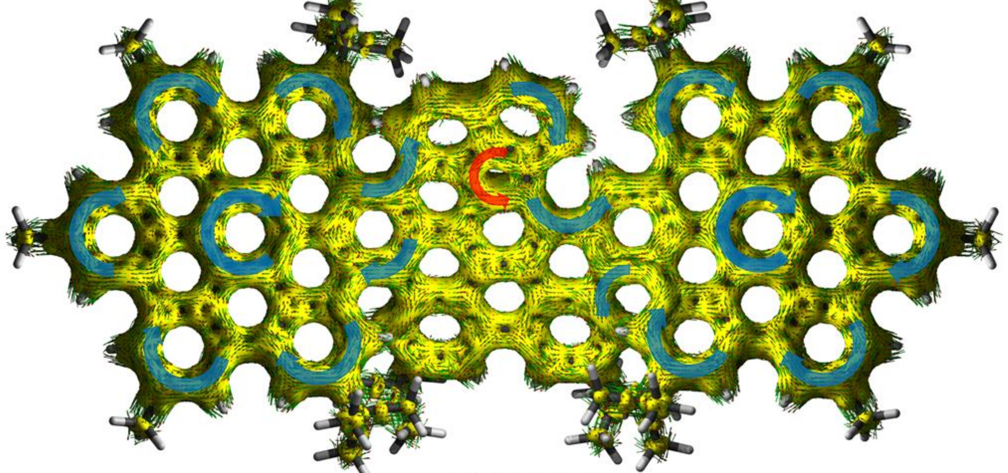

(*P*,*P*,*M*)-**2**

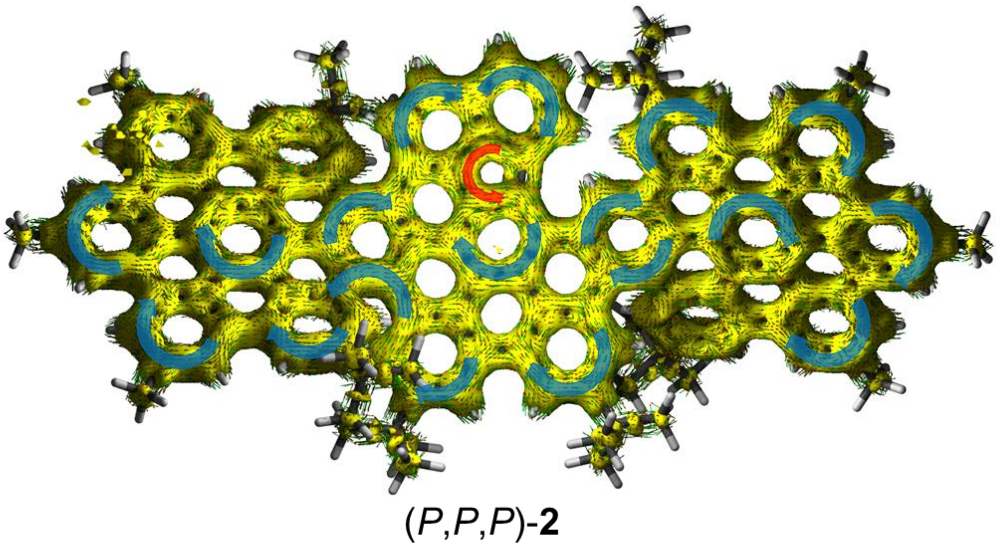

(*P*,*P*,*P*)-**2**

**Fig. S51** ACID plots (isovalue = 0.05) of compound (*P*,*M*)-**1** and (*P*,*M*,*M*)-**2**, diatropic (blue) and paratropic (red) ring currents are depicted for better visibility. Magnetic field oriented towards the spectator.

## 17. Cartesian coordinates

Compound (*P*,*M*)-**1** (*E* = -5472.510999 Hartrees, zero imaginary frequencies)

| | | | |
|---|---|---|---|
| C | -2.27053800 | 3.12605300 | -2.38906100 |
| C | -3.15067300 | 4.24076700 | -2.41886000 |
| C | -3.67328700 | 4.80213100 | -1.26894300 |
| C | -3.31050100 | 4.27215800 | 0.00000000 |
| C | -2.46241600 | 3.14866500 | 0.00000000 |
| C | -1.93724500 | 2.54808800 | -1.17322100 |
| C | -3.67328700 | 4.80213100 | 1.26894300 |
| C | -3.15067300 | 4.24076700 | 2.41886000 |
| C | -2.27053800 | 3.12605300 | 2.38906100 |
| C | -1.93724500 | 2.54808800 | 1.17322100 |
| C | -1.01662300 | 1.47616900 | 0.71240400 |
| C | -1.01662300 | 1.47616900 | -0.71240400 |
| C | -0.11909100 | 0.64856900 | 1.42826800 |
| C | 0.95990200 | 0.06777900 | 0.71326800 |
| C | 0.95990200 | 0.06777900 | -0.71326800 |
| C | -0.11909100 | 0.64856900 | -1.42826800 |
| C | -0.21240800 | 0.40451800 | -2.86660400 |
| C | -0.37601100 | 0.13910600 | -5.66823600 |
| C | 2.02516000 | -0.58096600 | -1.43955500 |
| C | 4.01973100 | -2.02439800 | -2.86465200 |
| C | 2.02516000 | -0.58096600 | 1.43955500 |
| C | 4.01973100 | -2.02439800 | 2.86465200 |
| C | -0.21240800 | 0.40451800 | 2.86660400 |
| C | -0.37601100 | 0.13910600 | 5.66823600 |
| C | -0.45860300 | 0.01564800 | -7.10536200 |
| C | -0.45860300 | 0.01564800 | 7.10536200 |
| C | 0.95408000 | 0.08249200 | -3.60149100 |
| C | 0.88674200 | 0.23758700 | -5.02755200 |
| C | -1.57049600 | 0.13173500 | -4.88388600 |
| C | -1.45324500 | 0.38400200 | -3.52003800 |
| C | 3.04414800 | -1.28491200 | -0.73527300 |
| C | 4.01884600 | -1.97337400 | -1.46600100 |
| C | 3.03399500 | -1.30598900 | -3.53724700 |
| C | 2.05688000 | -0.54756700 | -2.86589500 |
| C | 2.05688000 | -0.54756700 | 2.86589500 |
| C | 3.03399500 | -1.30598900 | 3.53724700 |
| C | 4.01884600 | -1.97337400 | 1.46600100 |
| C | 3.04414800 | -1.28491200 | 0.73527300 |
| C | -1.45324500 | 0.38400200 | 3.52003800 |
| C | -1.57049600 | 0.13173500 | 4.88388600 |
| C | 0.88674200 | 0.23758700 | 5.02755200 |
| C | 0.95408000 | 0.08249200 | 3.60149100 |
| C | 0.71780600 | 0.10489100 | -7.89309200 |
| C | 0.65141100 | -0.05741200 | -9.30229100 |
| C | -0.59177200 | -0.34802900 | -9.92219300 |
| C | -1.77797000 | -0.41506500 | -9.13783800 |
| C | -1.71337000 | -0.21933400 | -7.73205500 |
| C | -1.71337000 | -0.21933400 | 7.73205500 |
| C | -1.77797000 | -0.41506500 | 9.13783800 |
| C | -0.59177200 | -0.34802900 | 9.92219300 |
| C | 0.65141100 | -0.05741200 | 9.30229100 |
| C | 0.71780600 | 0.10489100 | 7.89309200 |
| C | -2.91952900 | -0.26847900 | -6.93839900 |

| | | | |
|---|---|---|---|
| C | -5.29876200 | -0.33395300 | -5.37136700 |
| C | -3.04876200 | -0.68007300 | -9.77307900 |
| C | -5.54470800 | -1.17277800 | -11.02653300 |
| C | -0.64765800 | -0.57995300 | -11.34638300 |
| C | -0.74187300 | -1.14378900 | -14.12304600 |
| C | 1.84963400 | 0.07560900 | -10.09746300 |
| C | 4.20595500 | 0.31592900 | -11.65376600 |
| C | 1.96342300 | 0.45506000 | -7.26300500 |
| C | 4.28107100 | 1.57432300 | -6.05546000 |
| C | -2.91952900 | -0.26847900 | 6.93839900 |
| C | -5.29876200 | -0.33395300 | 5.37136700 |
| C | -3.04876200 | -0.68007300 | 9.77307900 |
| C | -5.54470800 | -1.17277800 | 11.02653300 |
| C | -0.64765800 | -0.57995300 | 11.34638300 |
| C | -0.74187300 | -1.14378900 | 14.12304600 |
| C | 1.84963400 | 0.07560900 | 10.09746300 |
| C | 4.20595500 | 0.31592900 | 11.65376600 |
| C | 1.96342300 | 0.45506000 | 7.26300500 |
| C | 4.28107100 | 1.57432300 | 6.05546000 |
| C | -4.05174900 | -0.12949400 | -4.78219100 |
| C | -2.86488600 | -0.08371000 | -5.53015000 |
| C | -4.18161300 | -0.49830300 | -7.55427500 |
| C | -5.33349300 | -0.51593800 | -6.75712900 |
| C | -5.46495400 | -0.96434400 | -9.65097400 |
| C | -4.24710900 | -0.71492600 | -9.00288700 |
| C | -3.11699000 | -0.90782900 | -11.17787400 |
| C | -4.36580700 | -1.14410800 | -11.76919900 |
| C | -1.90107600 | -1.16945900 | -13.34894500 |
| C | -1.88735600 | -0.88858000 | -11.97573100 |
| C | 0.54033300 | -0.52887500 | -12.13196400 |
| C | 0.46267200 | -0.82107000 | -13.50111300 |
| C | 2.99290100 | -0.03292200 | -12.24402900 |
| C | 1.81113000 | -0.16570400 | -11.49922300 |
| C | 3.07784200 | 0.46620400 | -9.48922900 |
| C | 4.22871700 | 0.56440500 | -10.28100100 |
| C | 4.23525200 | 1.30339600 | -7.43052700 |
| C | 3.11273400 | 0.74575600 | -8.05064700 |
| C | 2.04118900 | 0.59199800 | -5.85165200 |
| C | 3.18288700 | 1.19286400 | -5.28795200 |
| C | -2.86488600 | -0.08371000 | 5.53015000 |
| C | -4.05174900 | -0.12949400 | 4.78219100 |
| C | -5.33349300 | -0.51593800 | 6.75712900 |
| C | -4.18161300 | -0.49830300 | 7.55427500 |
| C | -4.24710900 | -0.71492600 | 9.00288700 |
| C | -5.46495400 | -0.96434400 | 9.65097400 |
| C | -4.36580700 | -1.14410800 | 11.76919900 |
| C | -3.11699000 | -0.90782900 | 11.17787400 |
| C | -1.88735600 | -0.88858000 | 11.97573100 |
| C | -1.90107600 | -1.16945900 | 13.34894500 |
| C | 0.46267200 | -0.82107000 | 13.50111300 |
| C | 0.54033300 | -0.52887500 | 12.13196400 |
| C | 1.81113000 | -0.16570400 | 11.49922300 |
| C | 2.99290100 | -0.03292200 | 12.24402900 |
| C | 4.22871700 | 0.56440500 | 10.28100100 |
| C | 3.07784200 | 0.46620400 | 9.48922900 |
| C | 3.11273400 | 0.74575600 | 8.05064700 |
| C | 4.23525200 | 1.30339600 | 7.43052700 |

| | | | |
|---|---|---|---|
| C | 3.18288700 | 1.19286400 | 5.28795200 |
| C | 2.04118900 | 0.59199800 | 5.85165200 |
| C | 5.07417300 | -2.87758000 | -3.59567100 |
| C | 4.92030000 | -4.35697000 | -3.16783300 |
| C | 6.49105100 | -2.38327000 | -3.21881500 |
| C | 4.92966700 | -2.80328200 | -5.12737200 |
| C | 5.07417300 | -2.87758000 | 3.59567100 |
| C | 4.92966700 | -2.80328200 | 5.12737200 |
| C | 6.49105100 | -2.38327000 | 3.21881500 |
| C | 4.92030000 | -4.35697000 | 3.16783300 |
| C | -6.60814100 | -0.37158400 | -4.56052100 |
| C | -7.28400100 | -1.75214500 | -4.73863000 |
| C | -7.56065400 | 0.73503200 | -5.07322500 |
| C | -6.37310300 | -0.14214700 | -3.05555800 |
| C | 5.50552200 | 2.29459300 | -5.45910800 |
| C | 5.61258200 | 3.70738100 | -6.08251000 |
| C | 6.79223200 | 1.49849400 | -5.78101500 |
| C | 5.40649500 | 2.44781400 | -3.92963200 |
| C | -6.60814100 | -0.37158400 | 4.56052100 |
| C | -7.56065400 | 0.73503200 | 5.07322500 |
| C | -7.28400100 | -1.75214500 | 4.73863000 |
| C | -6.37310300 | -0.14214700 | 3.05555800 |
| C | 5.50552200 | 2.29459300 | 5.45910800 |
| C | 5.61258200 | 3.70738100 | 6.08251000 |
| C | 5.40649500 | 2.44781400 | 3.92963200 |
| C | 6.79223200 | 1.49849400 | 5.78101500 |
| H | -1.87281100 | 2.75997200 | -3.32676700 |
| H | -3.40761000 | 4.66611800 | -3.38442300 |
| H | -4.33688000 | 5.66028400 | -1.32771100 |
| H | -4.33688000 | 5.66028400 | 1.32771100 |
| H | -3.40761000 | 4.66611800 | 3.38442300 |
| H | -1.87281100 | 2.75997200 | 3.32676700 |
| H | -2.33684100 | 0.50522400 | -2.91071600 |
| H | 4.79188000 | -2.51433900 | -0.93700200 |
| H | 2.99385700 | -1.34929300 | -4.61425100 |
| H | 2.99385700 | -1.34929300 | 4.61425100 |
| H | 4.79188000 | -2.51433900 | 0.93700200 |
| H | -2.33684100 | 0.50522400 | 2.91071600 |
| H | -3.99752000 | -0.00969800 | -3.71027500 |
| H | -6.29497400 | -0.68041400 | -7.22464200 |
| H | -6.38614900 | -1.00087800 | -9.08225900 |
| H | -4.43889200 | -1.30679800 | -12.83764300 |
| H | -2.83046200 | -1.42812800 | -13.84178200 |
| H | 1.35819600 | -0.80881700 | -14.11061900 |
| H | 2.98258100 | -0.20090200 | -13.31419500 |
| H | 5.17738100 | 0.82620600 | -9.82654700 |
| H | 5.08622000 | 1.58162800 | -8.03886400 |
| H | 3.18498800 | 1.37750100 | -4.22373900 |
| H | -3.99752000 | -0.00969800 | 3.71027500 |
| H | -6.29497400 | -0.68041400 | 7.22464200 |
| H | -6.38614900 | -1.00087800 | 9.08225900 |
| H | -4.43889200 | -1.30679800 | 12.83764300 |
| H | -2.83046200 | -1.42812800 | 13.84178200 |
| H | 1.35819600 | -0.80881700 | 14.11061900 |
| H | 2.98258100 | -0.20090200 | 13.31419500 |
| H | 5.17738100 | 0.82620600 | 9.82654700 |
| H | 5.08622000 | 1.58162800 | 8.03886400 |

| | | | |
|---|---|---|---|
| H | 3.18498800 | 1.37750100 | 4.22373900 |
| H | 3.92725200 | -4.73789600 | -3.42786700 |
| H | 5.66621600 | -4.97770100 | -3.67642400 |
| H | 5.05772200 | -4.48650700 | -2.09020500 |
| H | 6.67940400 | -2.45705600 | -2.14366300 |
| H | 6.63353100 | -1.33797300 | -3.51226300 |
| H | 7.24951000 | -2.98592300 | -3.73035200 |
| H | 5.03883700 | -1.77975700 | -5.49979700 |
| H | 5.70768200 | -3.41271400 | -5.59783000 |
| H | 3.96180800 | -3.18791800 | -5.46486400 |
| H | 3.96180800 | -3.18791800 | 5.46486400 |
| H | 5.70768200 | -3.41271400 | 5.59783000 |
| H | 5.03883700 | -1.77975700 | 5.49979700 |
| H | 6.67940400 | -2.45705600 | 2.14366300 |
| H | 7.24951000 | -2.98592300 | 3.73035200 |
| H | 6.63353100 | -1.33797300 | 3.51226300 |
| H | 5.66621600 | -4.97770100 | 3.67642400 |
| H | 3.92725200 | -4.73789600 | 3.42786700 |
| H | 5.05772200 | -4.48650700 | 2.09020500 |
| H | -6.63412300 | -2.55569600 | -4.37686700 |
| H | -8.22023000 | -1.79075100 | -4.17096100 |
| H | -7.52167600 | -1.95870200 | -5.78632800 |
| H | -7.11073900 | 1.72592500 | -4.95217700 |
| H | -8.49918800 | 0.71681400 | -4.50837900 |
| H | -7.80734900 | 0.60522200 | -6.13110700 |
| H | -5.73455000 | -0.91689400 | -2.61876700 |
| H | -5.91525400 | 0.83244400 | -2.85733500 |
| H | -7.33153600 | -0.17006600 | -2.52783600 |
| H | 4.71819300 | 4.29999700 | -5.86398300 |
| H | 6.47986300 | 4.23754200 | -5.67358100 |
| H | 5.72936000 | 3.66406000 | -7.16956500 |
| H | 6.75213500 | 0.49385600 | -5.34744700 |
| H | 7.66603300 | 2.01190300 | -5.36526000 |
| H | 6.95318500 | 1.39245300 | -6.85775700 |
| H | 5.33162600 | 1.47818400 | -3.42682600 |
| H | 6.30375600 | 2.94910900 | -3.55329900 |
| H | 4.54363000 | 3.05350800 | -3.63466100 |
| H | -7.11073900 | 1.72592500 | 4.95217700 |
| H | -8.49918800 | 0.71681400 | 4.50837900 |
| H | -7.80734900 | 0.60522200 | 6.13110700 |
| H | -6.63412300 | -2.55569600 | 4.37686700 |
| H | -8.22023000 | -1.79075100 | 4.17096100 |
| H | -7.52167600 | -1.95870200 | 5.78632800 |
| H | -7.33153600 | -0.17006600 | 2.52783600 |
| H | -5.91525400 | 0.83244400 | 2.85733500 |
| H | -5.73455000 | -0.91689400 | 2.61876700 |
| H | 5.72936000 | 3.66406000 | 7.16956500 |
| H | 6.47986300 | 4.23754200 | 5.67358100 |
| H | 4.71819300 | 4.29999700 | 5.86398300 |
| H | 5.33162600 | 1.47818400 | 3.42682600 |
| H | 6.30375600 | 2.94910900 | 3.55329900 |
| H | 4.54363000 | 3.05350800 | 3.63466100 |
| H | 7.66603300 | 2.01190300 | 5.36526000 |
| H | 6.95318500 | 1.39245300 | 6.85775700 |
| H | 6.75213500 | 0.49385600 | 5.34744700 |
| C | 5.47142500 | 0.40001900 | 12.47370400 |
| H | 6.03095200 | -0.54260400 | 12.43165200 |

| | | | |
|---|---|---|---|
| H | 6.13535000 | 1.18712300 | 12.10419000 |
| H | 5.25254800 | 0.60297100 | 13.52571500 |
| C | -0.79561600 | -1.43194100 | 15.60453800 |
| H | -1.00181000 | -0.51858600 | 16.17610600 |
| H | -1.58612600 | -2.14932600 | 15.84324100 |
| H | 0.15387400 | -1.83559300 | 15.96730500 |
| C | -6.87797400 | -1.39792100 | 11.69917100 |
| H | -7.32636400 | -0.44701000 | 12.01246400 |
| H | -7.58796900 | -1.88537800 | 11.02487400 |
| H | -6.77421400 | -2.01862100 | 12.59372900 |
| C | 5.47142500 | 0.40001900 | -12.47370400 |
| H | 6.13535000 | 1.18712300 | -12.10419000 |
| H | 6.03095200 | -0.54260400 | -12.43165200 |
| H | 5.25254800 | 0.60297100 | -13.52571500 |
| C | -0.79561600 | -1.43194100 | -15.60453800 |
| H | -1.58612600 | -2.14932600 | -15.84324100 |
| H | -1.00181000 | -0.51858600 | -16.17610600 |
| H | 0.15387400 | -1.83559300 | -15.96730500 |
| C | -6.87797400 | -1.39792100 | -11.69917100 |
| H | -7.58796900 | -1.88537800 | -11.02487400 |
| H | -7.32636400 | -0.44701000 | -12.01246400 |
| H | -6.77421400 | -2.01862100 | -12.59372900 |

Compound (*P*,*P*)-**1** – (*E* = -5472.506179 Hartrees, zero imaginary frequencies)

| | | | |
|---|---|---|---|
| C | -2.29834800 | 3.78628600 | 0.65381600 |
| C | -2.31102200 | 5.20565900 | 0.72342800 |
| C | -1.21355500 | 5.97457000 | 0.38066600 |
| C | -0.00012300 | 5.33754400 | 0.00012100 |
| C | -0.00007400 | 3.93033000 | 0.00018000 |
| C | -1.14517000 | 3.13238400 | 0.24667000 |
| C | 1.21326600 | 5.97462000 | -0.38048300 |
| C | 2.31080400 | 5.20575800 | -0.72312300 |
| C | 2.29822600 | 3.78637900 | -0.65340100 |
| C | 1.14506300 | 3.13242700 | -0.24626900 |
| C | 0.71124100 | 1.72070500 | -0.06750000 |
| C | -0.71130500 | 1.72068300 | 0.06793400 |
| C | 1.43727000 | 0.50902400 | -0.03501400 |
| C | 0.71113600 | -0.71276100 | -0.05214800 |
| C | -0.71118800 | -0.71277700 | 0.05236100 |
| C | -1.43732600 | 0.50900100 | 0.03534700 |
| C | -2.90021900 | 0.44046100 | -0.05362700 |
| C | -5.72338200 | 0.40102400 | -0.03369400 |
| C | -1.42702300 | -1.96225900 | 0.17312300 |
| C | -2.85199100 | -4.42620800 | 0.24878000 |
| C | 1.42697100 | -1.96224000 | -0.17301300 |
| C | 2.85196400 | -4.42616100 | -0.24876100 |
| C | 2.90017200 | 0.44048600 | 0.05389400 |
| C | 5.72336200 | 0.40110100 | 0.03372700 |
| C | -7.16757700 | 0.38436700 | -0.01301100 |
| C | 7.16756200 | 0.38442300 | 0.01298100 |
| C | -3.57791900 | -0.70983400 | 0.41560400 |
| C | -4.98453900 | -0.57900900 | 0.67675600 |
| C | -5.03244700 | 1.38355800 | -0.80665000 |
| C | -3.64570900 | 1.43700400 | -0.70110400 |
| C | -0.73021700 | -3.20081200 | 0.07953900 |
| C | -1.45919900 | -4.39506000 | 0.12296200 |
| C | -3.51228300 | -3.20827400 | 0.39411400 |
| C | -2.83553300 | -1.97482000 | 0.40043300 |
| C | 2.83548200 | -1.97476300 | -0.40032500 |
| C | 3.51224900 | -3.20820900 | -0.39403400 |
| C | 1.45915800 | -4.39505200 | -0.12303600 |
| C | 0.73016900 | -3.20080700 | -0.07953300 |
| C | 3.64569800 | 1.43702000 | 0.70133500 |
| C | 5.03246100 | 1.38364000 | 0.80670300 |
| C | 4.98445800 | -0.57896500 | -0.67662700 |
| C | 3.57785000 | -0.70976800 | -0.41543500 |
| C | -7.85416400 | -0.53823900 | 0.81714500 |
| C | -9.27322800 | -0.58915100 | 0.81180200 |
| C | -10.00694200 | 0.25558700 | -0.06174200 |
| C | -9.32165200 | 1.19440900 | -0.88389700 |
| C | -7.90345300 | 1.26975300 | -0.84759000 |
| C | 7.90351300 | 1.27010100 | 0.84716700 |
| C | 9.32171800 | 1.19484500 | 0.88328700 |
| C | 10.00695000 | 0.25559800 | 0.06156700 |
| C | 9.27314900 | -0.58958600 | -0.81146300 |

| | | | |
|---|---|---|---|
| C | 7.85408800 | -0.53855700 | -0.81681600 |
| C | -7.20839900 | 2.24003200 | -1.66167900 |
| C | -5.83796800 | 4.20029800 | -3.21032400 |
| C | -10.06929600 | 2.06748700 | -1.75993500 |
| C | -11.53711400 | 3.77162500 | -3.48718700 |
| C | -11.44635000 | 0.15575000 | -0.11712300 |
| C | -14.26402100 | -0.10409200 | -0.29367100 |
| C | -9.96211300 | -1.50121800 | 1.69442600 |
| C | -11.31369800 | -3.30095900 | 3.41657400 |
| C | -7.10313000 | -1.36195200 | 1.72763600 |
| C | -5.62201400 | -2.65492900 | 3.78179500 |
| C | 7.20850700 | 2.24052200 | 1.66115100 |
| C | 5.83809400 | 4.20067100 | 3.20999000 |
| C | 10.06945100 | 2.06842700 | 1.75877700 |
| C | 11.53748400 | 3.77373300 | 3.48468600 |
| C | 11.44639500 | 0.15595600 | 0.11667200 |
| C | 14.26428700 | -0.10247500 | 0.29180100 |
| C | 9.96192200 | -1.50257000 | -1.69325000 |
| C | 11.31297100 | -3.30559800 | -3.41237800 |
| C | 7.10299900 | -1.36251500 | -1.72703800 |
| C | 5.62203800 | -2.65513900 | -3.78150300 |
| C | -5.14079900 | 3.29576300 | -2.41038500 |
| C | -5.78958900 | 2.32334800 | -1.63331400 |
| C | -7.93424900 | 3.13457500 | -2.49691900 |
| C | -7.23177800 | 4.09397100 | -3.23759200 |
| C | -10.14803000 | 3.85439700 | -3.41340700 |
| C | -9.39510400 | 3.03100800 | -2.56460700 |
| C | -11.48891300 | 1.97021000 | -1.83207700 |
| C | -12.18675700 | 2.82801700 | -2.69364000 |
| C | -13.58171000 | 0.82609200 | -1.07631300 |
| C | -12.19024700 | 0.98112100 | -1.00817800 |
| C | -12.13497500 | -0.78714300 | 0.70012600 |
| C | -13.52794800 | -0.89750500 | 0.58417000 |
| C | -12.02060700 | -2.51029700 | 2.51391700 |
| C | -11.37927400 | -1.61412200 | 1.64456100 |
| C | -9.23068600 | -2.28475100 | 2.63497300 |
| C | -9.92455700 | -3.17317800 | 3.46495500 |
| C | -7.01743500 | -2.77208000 | 3.70603600 |
| C | -7.77167200 | -2.15322100 | 2.70396600 |
| C | -5.68304200 | -1.34172800 | 1.70955700 |
| C | -4.97979400 | -1.95064800 | 2.76609500 |
| C | 5.78966600 | 2.32357400 | 1.63313900 |
| C | 5.14090200 | 3.29592800 | 2.41030700 |
| C | 7.23193500 | 4.09465800 | 3.23685800 |
| C | 7.93440300 | 3.13534900 | 2.49605200 |
| C | 9.39530900 | 3.03218200 | 2.56324200 |
| C | 10.14835600 | 3.85615700 | 3.41135300 |
| C | 12.18706500 | 2.82987100 | 2.69139600 |
| C | 11.48913300 | 1.97150000 | 1.83046800 |
| C | 12.19040300 | 0.98219600 | 1.00679500 |
| C | 13.58201500 | 0.82787800 | 1.07422800 |
| C | 13.52802500 | -0.89701100 | -0.58490800 |
| C | 12.13497800 | -0.78749100 | -0.70003900 |

| | | | |
|---|---|---|---|
| C | 11.37903800 | -1.61590500 | -1.64308400 |
| C | 12.02009200 | -2.51389800 | -2.51075300 |
| C | 9.92396300 | -3.17690900 | -3.46154200 |
| C | 9.23037700 | -2.28684700 | -2.63306700 |
| C | 7.77150600 | -2.15442700 | -2.70286700 |
| C | 7.01738200 | -2.77300500 | -3.70519600 |
| C | 4.97976000 | -1.95058400 | -2.76602000 |
| C | 5.68292500 | -1.34183600 | -1.70932500 |
| C | -3.59504800 | -5.77539100 | 0.19900200 |
| C | -3.06320900 | -6.70306500 | 1.31729200 |
| C | -3.34783800 | -6.44216200 | -1.17564500 |
| C | -5.11468200 | -5.61364600 | 0.38944700 |
| C | 3.59506800 | -5.77532300 | -0.19900800 |
| C | 5.11473000 | -5.61347900 | -0.38914200 |
| C | 3.06347000 | -6.70287300 | -1.31750600 |
| C | 3.34762500 | -6.44228000 | 1.17551000 |
| C | -5.13564600 | 5.28357600 | -4.05091900 |
| C | -5.64616400 | 6.67928700 | -3.61967000 |
| C | -5.45749400 | 5.06023700 | -5.54795300 |
| C | -3.60509100 | 5.25962600 | -3.87857200 |
| C | -4.86564400 | -3.27860200 | 4.97022500 |
| C | -5.14322800 | -4.79956400 | 5.02435700 |
| C | -5.35595500 | -2.62475900 | 6.28444000 |
| C | -3.34267000 | -3.07265300 | 4.86668200 |
| C | 5.13573300 | 5.28383900 | 4.05070400 |
| C | 5.64594000 | 6.67964200 | 3.61939000 |
| C | 5.45786200 | 5.06053400 | 5.54768300 |
| C | 3.60514900 | 5.25963700 | 3.87860200 |
| C | 4.86582400 | -3.27834200 | -4.97027300 |
| C | 5.35683400 | -2.62444100 | -6.28420200 |
| C | 3.34289700 | -3.07184300 | -4.86719300 |
| C | 5.14285800 | -4.79939300 | -5.02459400 |
| H | -1.99340000 | -6.90527200 | 1.20994300 |
| H | -3.58611600 | -7.66526900 | 1.28922900 |
| H | -3.22205500 | -6.25952100 | 2.30578100 |
| H | -2.28380500 | -6.62293300 | -1.35520900 |
| H | -3.72348900 | -5.81243600 | -1.98878500 |
| H | -3.86422800 | -7.40703200 | -1.22650700 |
| H | -5.35905800 | -5.15147600 | 1.35121500 |
| H | -5.59422300 | -6.59719800 | 0.36192500 |
| H | -5.56293500 | -5.00729300 | -0.40421500 |
| H | 5.35927700 | -5.15128500 | -1.35086000 |
| H | 5.59435400 | -6.59698800 | -0.36150700 |
| H | 5.56276300 | -5.00707900 | 0.40461800 |
| H | 3.22246600 | -6.25920200 | -2.30591000 |
| H | 1.99364600 | -6.90512600 | -1.21035900 |
| H | 3.58640600 | -7.66506600 | -1.28946700 |
| H | 3.72298300 | -5.81258900 | 1.98881400 |
| H | 3.86414700 | -7.40708500 | 1.22638300 |
| H | 2.28357300 | -6.62323700 | 1.35479500 |
| H | -6.72634800 | 6.78201300 | -3.75867800 |
| H | -5.15883200 | 7.45957000 | -4.21446700 |
| H | -5.42384200 | 6.86834300 | -2.56428500 |

| | | | |
|---|---|---|---|
| H | -6.53228200 | 5.11055900 | -5.74615000 |
| H | -5.09978300 | 4.08138500 | -5.88384200 |
| H | -4.96963200 | 5.82809900 | -6.15837300 |
| H | -3.30745700 | 5.43540200 | -2.83968900 |
| H | -3.15622500 | 6.04961300 | -4.48881600 |
| H | -3.17178500 | 4.30785000 | -4.20298200 |
| H | -6.20850500 | -5.01931200 | 5.14050800 |
| H | -4.61721100 | -5.24886400 | 5.87378500 |
| H | -4.79595400 | -5.29434700 | 4.11129200 |
| H | -6.42826800 | -2.77732000 | 6.43935000 |
| H | -5.16720100 | -1.54625500 | 6.27964000 |
| H | -4.82991400 | -3.05805700 | 7.14225700 |
| H | -2.93042700 | -3.52896900 | 3.96089700 |
| H | -2.85101000 | -3.53874100 | 5.72633200 |
| H | -3.07265000 | -2.01162600 | 4.86853200 |
| H | 6.72612700 | 6.78255300 | 3.75823800 |
| H | 5.15855800 | 7.45982900 | 4.21427400 |
| H | 5.42341800 | 6.86867200 | 2.56404400 |
| H | 6.53267900 | 5.11096500 | 5.74567900 |
| H | 5.10031100 | 4.08164200 | 5.88362200 |
| H | 4.97002200 | 5.82833800 | 6.15818800 |
| H | 3.30733000 | 5.43533400 | 2.83976200 |
| H | 3.15625200 | 6.04957200 | 4.48888800 |
| H | 3.17203500 | 4.30780200 | 4.20310700 |
| H | 5.16852900 | -1.54586900 | -6.27924800 |
| H | 4.83090100 | -3.05735800 | -7.14228000 |
| H | 6.42913600 | -2.77741900 | -6.43877700 |
| H | 3.07326700 | -2.01072300 | -4.86898600 |
| H | 2.93019800 | -3.52813000 | -3.96159900 |
| H | 2.85133800 | -3.53763700 | -5.72706700 |
| H | 6.20809000 | -5.01952300 | -5.14045200 |
| H | 4.61693800 | -5.24833400 | -5.87427200 |
| H | 4.79511100 | -5.29421400 | -4.11173000 |
| H | 14.16397700 | 1.43616100 | 1.75590900 |
| H | 14.06691600 | -1.62159700 | -1.18334700 |
| H | 13.09908900 | -2.60814100 | -2.49532700 |
| H | 9.38007300 | -3.80360700 | -4.15908900 |
| H | 13.26690900 | 2.77447300 | 2.75679000 |
| H | 9.65376800 | 4.58377800 | 4.04345000 |
| H | 4.06228100 | 3.34559000 | 2.39051400 |
| H | 7.78337200 | 4.78933800 | 3.85618700 |
| H | 7.52993000 | -3.32918400 | -4.47961200 |
| H | 3.90508300 | -1.84412900 | -2.78702400 |
| H | 4.58746600 | -3.19908000 | -0.47657300 |
| H | 0.93614100 | -5.33663700 | -0.02480300 |
| H | -0.93615900 | -5.33661700 | 0.02464400 |
| H | -4.58750300 | -3.19915200 | 0.47669800 |
| H | -3.90508200 | -1.84461300 | 2.78676300 |
| H | -7.52987900 | -3.32808400 | 4.48063500 |
| H | -9.38091800 | -3.79905300 | 4.16343900 |
| H | -13.09967600 | -2.60371000 | 2.49912100 |
| H | -14.06679700 | -1.62207500 | 1.18266600 |
| H | -14.16353000 | 1.43352400 | -1.75887600 |

| | | | |
|---|---|---|---|
| H | -13.26657800 | 2.77244700 | -2.75930600 |
| H | -9.65337500 | 4.58177500 | -4.04572900 |
| H | -7.78319500 | 4.78852000 | -3.85707800 |
| H | -4.06219300 | 3.34564600 | -2.39032300 |
| H | -3.09861400 | 2.22672900 | -1.19130600 |
| H | -3.20000500 | 3.24865000 | 0.91904900 |
| H | -3.22669100 | 5.69532800 | 1.04110800 |
| H | -1.26960800 | 7.05895600 | 0.41317500 |
| H | 1.26923600 | 7.05901500 | -0.41312300 |
| H | 3.22644200 | 5.69545100 | -1.04082900 |
| H | 3.19992200 | 3.24879000 | -0.91856600 |
| H | 3.09863100 | 2.22671400 | 1.19162100 |
| C | 12.02456000 | -4.29577000 | -4.30343200 |
| H | 11.90892400 | -5.31908800 | -3.92615300 |
| H | 11.61977300 | -4.28018300 | -5.32024900 |
| H | 13.09571600 | -4.08454200 | -4.35948500 |
| C | 15.76714200 | -0.22700600 | 0.37241700 |
| H | 16.09680000 | -1.24252900 | 0.13521400 |
| H | 16.25696400 | 0.44930200 | -0.33900400 |
| H | 16.13608100 | 0.02811900 | 1.36995900 |
| C | 12.32176200 | 4.70141100 | 4.38190900 |
| H | 12.61023700 | 5.61504400 | 3.84758400 |
| H | 11.73416100 | 5.00587200 | 5.25274500 |
| H | 13.24195200 | 4.22931000 | 4.73792300 |
| C | -12.32134700 | 4.69864200 | -4.38513400 |
| H | -12.61132600 | 5.61191600 | -3.85100700 |
| H | -11.73313000 | 5.00378000 | -5.25530500 |
| H | -13.24069300 | 4.22566700 | -4.74218500 |
| C | -15.76673600 | -0.22951000 | -0.37562900 |
| H | -16.09647500 | -1.24362100 | -0.13265600 |
| H | -16.25784100 | 0.45112600 | 0.33075200 |
| H | -16.13424200 | 0.01937200 | -1.37529100 |
| C | -12.02556600 | -4.28878500 | 4.31001300 |
| H | -11.90541100 | -5.31364900 | 3.93840500 |
| H | -11.62449700 | -4.26687500 | 5.32819200 |
| H | -13.09751100 | -4.08044200 | 4.36117400 |

Compound (*P*,*M*,*M*)-**2** – (*E* = -5471.332429 Hartrees, zero imaginary frequencies)

| | | | |
|---|---|---|---|
| C | -2.77421500 | 2.71605500 | -0.94486700 |
| C | -3.17653400 | 3.82605400 | -1.78058300 |
| C | -2.25303000 | 4.64440700 | -2.41292800 |
| C | -0.82805900 | 4.44631100 | -2.30399400 |
| C | -0.44875600 | 3.37536300 | -1.49470900 |
| C | -1.40298300 | 2.56986200 | -0.86856300 |
| C | 0.24104800 | 5.11779000 | -2.95583900 |
| C | 1.53719600 | 4.64791200 | -2.77967500 |
| C | 1.87147600 | 3.51981400 | -1.97303700 |
| C | 0.86534100 | 2.84825500 | -1.28937700 |
| C | 0.68363800 | 1.59705100 | -0.45415300 |
| C | -0.71504600 | 1.49185700 | -0.28272800 |
| C | 1.44860900 | 0.51818400 | 0.05710300 |
| C | 0.74438900 | -0.63739900 | 0.56046800 |
| C | -0.67527900 | -0.69460700 | 0.62532500 |
| C | -1.42642200 | 0.41488000 | 0.20036700 |
| C | -2.86596200 | 0.46061900 | 0.14302000 |
| C | -5.65023100 | 0.40355100 | -0.06204900 |
| C | -1.38531200 | -1.79095100 | 1.20422600 |
| C | -2.78214600 | -3.71241600 | 2.72253600 |
| C | 1.48210100 | -1.77496500 | 1.05495100 |
| C | 2.96449300 | -3.89964300 | 2.23072800 |
| C | 2.90946400 | 0.49243400 | 0.09113300 |
| C | 5.72385400 | 0.45650300 | -0.10879600 |
| C | -7.09455800 | 0.37156600 | -0.17413100 |
| C | 7.16490300 | 0.43680400 | -0.21089000 |
| C | -3.55799700 | -0.73559900 | 0.47174100 |
| C | -4.92242100 | -0.81749800 | 0.06955200 |
| C | -4.96998900 | 1.65615700 | -0.09487300 |
| C | -3.55994200 | 1.64738500 | -0.29800500 |
| C | -0.66239200 | -2.81569500 | 1.86839300 |
| C | -1.38366300 | -3.75300900 | 2.61773800 |
| C | -3.47193100 | -2.73694500 | 1.99582800 |
| C | -2.81303100 | -1.78534700 | 1.19925500 |
| C | 2.89370500 | -1.84637700 | 0.88370400 |
| C | 3.60055000 | -2.88653800 | 1.51473900 |
| C | 1.56623600 | -3.86369600 | 2.30388100 |
| C | 0.80557400 | -2.82856100 | 1.74969800 |
| C | 3.64935200 | 1.68241100 | 0.05124800 |
| C | 5.04108200 | 1.69736300 | 0.07299100 |
| C | 4.98361200 | -0.75506800 | -0.15280600 |
| C | 3.59935700 | -0.73945000 | 0.22328300 |
| C | -7.77329200 | -0.86954500 | -0.29986800 |
| C | -9.18871900 | -0.90357200 | -0.42318500 |
| C | -9.93479000 | 0.30475000 | -0.38403200 |
| C | -9.25973200 | 1.54781300 | -0.25063300 |
| C | -7.84111800 | 1.58017600 | -0.16509400 |
| C | 7.91074200 | 1.63517400 | -0.03753300 |
| C | 9.33078400 | 1.59567400 | -0.05955000 |
| C | 10.00600200 | 0.36203200 | -0.27866700 |
| C | 9.25940600 | -0.82238400 | -0.51089900 |

| | | | |
|---|---|---|---|
| C | 7.84109600 | -0.78513700 | -0.45982800 |
| C | -7.16037900 | 2.83510700 | 0.02120000 |
| C | -5.83614800 | 5.23714600 | 0.77971500 |
| C | -10.01271000 | 2.77985200 | -0.20858200 |
| C | -11.47727300 | 5.20741200 | -0.23706600 |
| C | -11.37527800 | 0.26923300 | -0.48130000 |
| C | -14.20410700 | 0.19991100 | -0.65706800 |
| C | -9.86752400 | -2.16854800 | -0.58340200 |
| C | -11.19647000 | -4.66331500 | -0.79574200 |
| C | -7.01078400 | -2.08638300 | -0.41075900 |
| C | -5.47877300 | -4.38438100 | -1.08789400 |
| C | 7.22555400 | 2.88899500 | 0.17459600 |
| C | 5.86766200 | 5.35884600 | 0.59390800 |
| C | 10.09114500 | 2.80979800 | 0.13091900 |
| C | 11.58671200 | 5.20212800 | 0.41751000 |
| C | 11.44858500 | 0.30867100 | -0.25547900 |
| C | 14.27404500 | 0.18164700 | -0.06998900 |
| C | 9.93549700 | -2.06469900 | -0.80238100 |
| C | 11.26121000 | -4.50530600 | -1.36461300 |
| C | 7.07527600 | -1.96769000 | -0.75450800 |
| C | 5.54770300 | -4.14006100 | -1.76637800 |
| C | -5.12386200 | 4.06038700 | 0.55581800 |
| C | -5.74349100 | 2.87378900 | 0.12274100 |
| C | -7.89558900 | 4.04262300 | 0.17568200 |
| C | -7.21637500 | 5.20913000 | 0.54678300 |
| C | -10.09271900 | 5.21088700 | -0.06584400 |
| C | -9.34579900 | 4.02674200 | -0.03153200 |
| C | -11.42897900 | 2.76258400 | -0.35088200 |
| C | -12.12490400 | 3.98081100 | -0.36744200 |
| C | -13.52453800 | 1.41259400 | -0.55625100 |
| C | -12.12691200 | 1.47937600 | -0.46837300 |
| C | -12.05914600 | -0.97592600 | -0.59145300 |
| C | -13.45837900 | -0.97747100 | -0.67785700 |
| C | -11.91778700 | -3.47221500 | -0.73523500 |
| C | -11.28912000 | -2.22227000 | -0.63320800 |
| C | -9.12077200 | -3.37584100 | -0.69744300 |
| C | -9.80339900 | -4.59560900 | -0.78633600 |
| C | -6.87880700 | -4.42847500 | -1.05153400 |
| C | -7.65749500 | -3.31628100 | -0.71559500 |
| C | -5.59483800 | -2.06149100 | -0.30189600 |
| C | -4.86271200 | -3.19985000 | -0.68907400 |
| C | 5.80602700 | 2.93523200 | 0.22676100 |
| C | 5.16390400 | 4.16465700 | 0.44358900 |
| C | 7.26317700 | 5.29530100 | 0.53420600 |
| C | 7.96070100 | 4.09703800 | 0.33510700 |
| C | 9.42618400 | 4.05842500 | 0.30129100 |
| C | 10.19312600 | 5.22411900 | 0.43544300 |
| C | 12.22694200 | 3.97358400 | 0.26684200 |
| C | 11.51517000 | 2.77332800 | 0.13256100 |
| C | 12.20691500 | 1.48939800 | -0.01315300 |
| C | 13.60200500 | 1.39410900 | 0.08101100 |
| C | 13.52518200 | -0.96085400 | -0.34553200 |
| C | 12.12717900 | -0.92897100 | -0.45010700 |

| | | | |
|---|---|---|---|
| C | 11.35616500 | -2.13359700 | -0.76832900 |
| C | 11.98424300 | -3.35467300 | -1.05732000 |
| C | 9.86874200 | -4.42321200 | -1.40596500 |
| C | 9.18706400 | -3.22824300 | -1.14471900 |
| C | 7.72429900 | -3.15174500 | -1.20496300 |
| C | 6.94698000 | -4.20140400 | -1.70439500 |
| C | 4.92856200 | -3.01275400 | -1.23079300 |
| C | 5.65765200 | -1.94334800 | -0.67742600 |
| C | -3.50078500 | -4.70780500 | 3.65576500 |
| C | -3.08385800 | -4.41205900 | 5.11719000 |
| C | -3.09891600 | -6.15801300 | 3.29783100 |
| C | -5.03439800 | -4.60056800 | 3.55838900 |
| C | 3.73797700 | -5.02183800 | 2.94958700 |
| C | 5.25716100 | -4.93217200 | 2.71096400 |
| C | 3.25098300 | -6.40018300 | 2.44308300 |
| C | 3.48085000 | -4.91956900 | 4.47239100 |
| C | -5.16652700 | 6.53652300 | 1.26552000 |
| C | -5.80246600 | 6.97770600 | 2.60523600 |
| C | -5.38083800 | 7.64551500 | 0.20751000 |
| C | -3.65133900 | 6.36712600 | 1.48444700 |
| C | -4.68926100 | -5.60863100 | -1.58940800 |
| C | -5.05132700 | -5.86821900 | -3.07177900 |
| C | -5.06220100 | -6.85348500 | -0.75026100 |
| C | -3.16584200 | -5.40278200 | -1.49352500 |
| C | 5.17174000 | 6.71382100 | 0.82354400 |
| C | 5.53508300 | 7.67645500 | -0.33229200 |
| C | 5.65085900 | 7.31822000 | 2.16501300 |
| C | 3.63753200 | 6.58555000 | 0.87697300 |
| C | 4.76172800 | -5.28473600 | -2.43362500 |
| C | 5.16312600 | -5.37033000 | -3.92623500 |
| C | 3.23827500 | -5.07217400 | -2.35389800 |
| C | 5.09814100 | -6.62577300 | -1.73967600 |
| H | -4.22911900 | 4.02186600 | -1.94616300 |
| H | -2.62298700 | 5.44711800 | -3.04534900 |
| H | 0.05446300 | 5.96975500 | -3.60352700 |
| H | 2.34844700 | 5.15114700 | -3.29767500 |
| H | 2.90856500 | 3.20705800 | -1.94091900 |
| H | -0.84879900 | -4.51466300 | 3.17057800 |
| H | -4.54992900 | -2.69134100 | 2.05512500 |
| H | 4.67783600 | -2.88057600 | 1.45142900 |
| H | 1.05756600 | -4.66566100 | 2.82360900 |
| H | 3.09703100 | 2.60797500 | 0.09467800 |
| H | -4.06083200 | 4.04018300 | 0.74164900 |
| H | -7.78338200 | 6.11683600 | 0.70831000 |
| H | -9.59311300 | 6.16893100 | 0.02129700 |
| H | -13.20035600 | 3.98992600 | -0.49631900 |
| H | -14.11393600 | 2.32139400 | -0.54096600 |
| H | -13.99621100 | -1.91333300 | -0.76982100 |
| H | -12.99888300 | -3.53599100 | -0.76135600 |
| H | -9.24667400 | -5.52476000 | -0.82917600 |
| H | -7.37474600 | -5.34396300 | -1.34813300 |
| H | -3.78561000 | -3.13134300 | -0.70078100 |
| H | 4.08598700 | 4.18698000 | 0.50625900 |

| | | | |
|---|---|---|---|
| H | 7.82007200 | 6.21440500 | 0.65796700 |
| H | 9.70620200 | 6.18503000 | 0.55026700 |
| H | 13.30993200 | 3.96809700 | 0.24085900 |
| H | 14.19369700 | 2.27701200 | 0.29121300 |
| H | 14.05763900 | -1.89607600 | -0.47011700 |
| H | 13.06532200 | -3.42421900 | -1.05143700 |
| H | 9.31273000 | -5.32504700 | -1.63543800 |
| H | 7.44209200 | -5.07774300 | -2.10248500 |
| H | 3.85176800 | -2.93460500 | -1.25420500 |
| H | -3.36713000 | -3.39480400 | 5.40671500 |
| H | -3.57769300 | -5.11041600 | 5.80206200 |
| H | -2.00333500 | -4.51214600 | 5.25773000 |
| H | -2.02393700 | -6.32824600 | 3.40495700 |
| H | -3.37553300 | -6.40061300 | 2.26670900 |
| H | -3.61113900 | -6.86355300 | 3.96087300 |
| H | -5.39135200 | -4.79531500 | 2.54185000 |
| H | -5.49495600 | -5.33989900 | 4.22107900 |
| H | -5.39734200 | -3.61451300 | 3.86518700 |
| H | 5.67709600 | -3.99836100 | 3.09849300 |
| H | 5.75801500 | -5.75654900 | 3.22828500 |
| H | 5.50923800 | -5.00446000 | 1.64801500 |
| H | 2.18523400 | -6.55668100 | 2.63340800 |
| H | 3.79799800 | -7.20247700 | 2.95007200 |
| H | 3.41733700 | -6.50450000 | 1.36590900 |
| H | 4.01796900 | -5.71431000 | 5.00189200 |
| H | 3.82638400 | -3.95704700 | 4.86369700 |
| H | 2.41786000 | -5.01677900 | 4.71288800 |
| H | -5.66430400 | 6.21158800 | 3.37524800 |
| H | -5.33408800 | 7.90283200 | 2.95879300 |
| H | -6.87575600 | 7.16567100 | 2.50721700 |
| H | -4.92851300 | 7.36571300 | -0.74962500 |
| H | -4.91831800 | 8.58085600 | 0.54122000 |
| H | -6.44253900 | 7.84363100 | 0.03259300 |
| H | -3.43144300 | 5.61522900 | 2.24945700 |
| H | -3.13590500 | 6.07961300 | 0.56265300 |
| H | -3.22240600 | 7.31582800 | 1.82206200 |
| H | -4.79006900 | -5.00678400 | -3.69510400 |
| H | -4.50384600 | -6.73920500 | -3.44874400 |
| H | -6.12019500 | -6.06335300 | -3.20056900 |
| H | -4.81655100 | -6.70337100 | 0.30609000 |
| H | -4.50708300 | -7.72780900 | -1.10700000 |
| H | -6.12806700 | -7.09048900 | -0.81535200 |
| H | -2.84277800 | -5.21684700 | -0.46430400 |
| H | -2.65243600 | -6.30295900 | -1.84602100 |
| H | -2.82686000 | -4.56750200 | -2.11459100 |
| H | 5.19761300 | 7.27693600 | -1.29437300 |
| H | 5.05348500 | 8.64870500 | -0.18043700 |
| H | 6.61377200 | 7.84588900 | -0.39913400 |
| H | 5.40272600 | 6.65762500 | 3.00225800 |
| H | 5.16492100 | 8.28456800 | 2.33931600 |
| H | 6.73215300 | 7.48389600 | 2.17717300 |
| H | 3.19473600 | 7.57325300 | 1.03935900 |
| H | 3.22500000 | 6.18916700 | -0.05661800 |

| | | | |
|---|---|---|---|
| H | 3.31015800 | 5.93946000 | 1.69811400 |
| H | 6.23260900 | -5.56669900 | -4.04869900 |
| H | 4.61550800 | -6.18076800 | -4.41995600 |
| H | 4.93114600 | -4.43633700 | -4.44845000 |
| H | 2.88979200 | -5.00626600 | -1.31813100 |
| H | 2.72559500 | -5.91719500 | -2.82405800 |
| H | 2.92464200 | -4.16400200 | -2.87865900 |
| H | 4.54620000 | -7.44425700 | -2.21449400 |
| H | 6.16339700 | -6.86619600 | -1.80131200 |
| H | 4.82121400 | -6.59999700 | -0.68062500 |
| C | 12.38225200 | 6.47496700 | 0.58361300 |
| H | 11.85628100 | 7.33348700 | 0.15602200 |
| H | 12.55576000 | 6.69521500 | 1.64418500 |
| H | 13.36145800 | 6.39924500 | 0.10239400 |
| C | 15.77418500 | 0.10621800 | 0.08622500 |
| H | 16.05377100 | -0.02362400 | 1.13901200 |
| H | 16.19167200 | -0.73849700 | -0.46880900 |
| H | 16.25884500 | 1.02113900 | -0.26698600 |
| C | 11.96366900 | -5.81662100 | -1.62486300 |
| H | 11.97632800 | -6.44228400 | -0.72391600 |
| H | 11.46102500 | -6.39021700 | -2.40943000 |
| H | 13.00224700 | -5.65923100 | -1.92847200 |
| C | -15.71264600 | 0.16153500 | -0.71642800 |
| H | -16.06817800 | -0.70873900 | -1.27549000 |
| H | -16.14461800 | 0.10100500 | 0.29011800 |
| H | -16.11660300 | 1.06097500 | -1.18981900 |
| C | -12.25402000 | 6.50239900 | -0.25513400 |
| H | -12.42252500 | 6.87508700 | 0.76261000 |
| H | -11.71372800 | 7.28337900 | -0.79895700 |
| H | -13.23317100 | 6.37453300 | -0.72436500 |
| C | -11.90440700 | -5.99625600 | -0.84577400 |
| H | -11.33008700 | -6.73212300 | -1.41607400 |
| H | -12.04569600 | -6.40467100 | 0.16251400 |
| H | -12.89412800 | -5.90605700 | -1.30224600 |

Compound (*M*,*M*,*M*)-**2** – (*E* = - 5471.329757 Hartrees, zero imaginary frequencies)

| | | | |
|---|---|---|---|
| C | 2.82388700 | -2.79269900 | 0.64813700 |
| C | 3.23688900 | -4.17997400 | 0.65424800 |
| C | 2.32265200 | -5.22226200 | 0.69469600 |
| C | 0.89475100 | -5.01221600 | 0.71090900 |
| C | 0.50627200 | -3.67666100 | 0.61722500 |
| C | 1.45094100 | -2.64890900 | 0.59178700 |
| C | -0.17368800 | -5.94392900 | 0.80222400 |
| C | -1.48042300 | -5.46875300 | 0.81116600 |
| C | -1.82729800 | -4.08945300 | 0.69733600 |
| C | -0.81959800 | -3.14189100 | 0.56595500 |
| C | -0.65456000 | -1.64064600 | 0.43252400 |
| C | 0.74319300 | -1.44032700 | 0.48710400 |
| C | -1.43406700 | -0.46475500 | 0.29555000 |
| C | -0.75732400 | 0.81123800 | 0.32760300 |
| C | 0.66132600 | 0.91511300 | 0.29018100 |
| C | 1.43341300 | -0.25519300 | 0.37751000 |
| C | 2.87277300 | -0.30101400 | 0.35333400 |
| C | 5.64856400 | -0.30827400 | 0.10739800 |
| C | 1.35391100 | 2.16227700 | 0.24644800 |
| C | 2.72989200 | 4.60734500 | 0.51150700 |
| C | -1.51904800 | 2.03685000 | 0.34817200 |
| C | -3.02202200 | 4.44155700 | 0.10947900 |
| C | -2.88660200 | -0.45121500 | 0.12430600 |
| C | -5.70101200 | -0.53718000 | -0.02120000 |
| C | 7.08792200 | -0.32801600 | -0.05659500 |
| C | -7.14327000 | -0.56987700 | -0.10739600 |
| C | 3.53849200 | 0.91267100 | 0.03690900 |
| C | 4.89500600 | 0.79762300 | -0.38695900 |
| C | 5.00080700 | -1.40002200 | 0.75842500 |
| C | 3.59069800 | -1.53162200 | 0.59793600 |
| C | 0.61769600 | 3.37186500 | 0.32346900 |
| C | 1.32797300 | 4.57046900 | 0.46054900 |
| C | 3.43019800 | 3.40813600 | 0.34731300 |
| C | 2.78051100 | 2.17403100 | 0.17314400 |
| C | -2.94184300 | 2.00422300 | 0.42179000 |
| C | -3.65389000 | 3.20767000 | 0.26165800 |
| C | -1.62301000 | 4.46396900 | 0.16333100 |
| C | -0.85247100 | 3.30272500 | 0.28672700 |
| C | -3.55053600 | -1.55183700 | -0.43472100 |
| C | -4.92822300 | -1.56759700 | -0.63617400 |
| C | -5.04619500 | 0.53586100 | 0.63851300 |
| C | -3.63413000 | 0.70768300 | 0.45488700 |
| C | 7.73034600 | 0.66191700 | -0.84645000 |
| C | 9.13907900 | 0.62570500 | -1.03360600 |
| C | 9.91564900 | -0.37761800 | -0.39438600 |
| C | 9.27808900 | -1.35573100 | 0.41542000 |
| C | 7.86512000 | -1.34179600 | 0.56460000 |
| C | -7.78928000 | -1.56207300 | -0.89348600 |
| C | -9.20226900 | -1.53633500 | -1.04564500 |
| C | -9.97149700 | -0.53644900 | -0.38789800 |

| | | | |
|---|---|---|---|
| C | -9.33002400 | 0.41388000 | 0.44923300 |
| C | -7.91618100 | 0.40781800 | 0.56979300 |
| C | 7.22339400 | -2.29866700 | 1.42770100 |
| C | 5.98654600 | -3.88505600 | 3.43933300 |
| C | 10.06284400 | -2.36713400 | 1.08450900 |
| C | 11.58946700 | -4.40400200 | 2.33245400 |
| C | 11.34903200 | -0.40460000 | -0.56810800 |
| C | 14.16251700 | -0.45903200 | -0.91162100 |
| C | 9.77995800 | 1.61332400 | -1.87062600 |
| C | 11.04030500 | 3.61331900 | -3.43536600 |
| C | 6.93536200 | 1.64222400 | -1.54012500 |
| C | 5.32415600 | 3.27297000 | -3.22447600 |
| C | -7.00948300 | -2.58817900 | -1.54592300 |
| C | -5.46835600 | -4.62435200 | -2.81018100 |
| C | -9.85914400 | -2.52845700 | -1.86558000 |
| C | -11.15650600 | -4.49955300 | -3.43782400 |
| C | -11.40179700 | -0.47845600 | -0.57424400 |
| C | -14.19037700 | -0.28882200 | -1.04197900 |
| C | -10.10778600 | 1.38480300 | 1.18125600 |
| C | -11.63102100 | 3.30219600 | 2.60615300 |
| C | -7.26233500 | 1.33195400 | 1.45815600 |
| C | -5.99546500 | 2.79147400 | 3.54044100 |
| C | 5.23527700 | -3.03927900 | 2.62462200 |
| C | 5.81117000 | -2.28866700 | 1.58349600 |
| C | 7.99463100 | -3.21639100 | 2.19217200 |
| C | 7.35785000 | -3.98514000 | 3.17383200 |
| C | 10.21319900 | -4.31511900 | 2.54072500 |
| C | 9.43590700 | -3.31236100 | 1.94739100 |
| C | 11.47229000 | -2.42723900 | 0.89303000 |
| C | 12.19977800 | -3.44837400 | 1.52216100 |
| C | 13.51963300 | -1.41872700 | -0.13119500 |
| C | 12.13052600 | -1.41909700 | 0.05669600 |
| C | 11.99605400 | 0.58431100 | -1.36389500 |
| C | 13.38914000 | 0.53170700 | -1.51353100 |
| C | 11.79029800 | 2.62745300 | -2.79669600 |
| C | 11.19605600 | 1.62438400 | -2.01647100 |
| C | 9.00132300 | 2.58502700 | -2.56208100 |
| C | 9.65145100 | 3.57098500 | -3.31525000 |
| C | 6.72293300 | 3.31970700 | -3.28856000 |
| C | 7.54027500 | 2.53443500 | -2.46885000 |
| C | 5.52561900 | 1.67933200 | -1.36752700 |
| C | 4.75313100 | 2.46200700 | -2.24641800 |
| C | -5.59435100 | -2.61255200 | -1.41397500 |
| C | -4.85988800 | -3.62384500 | -2.05337700 |
| C | -6.86094600 | -4.58340400 | -2.93056500 |
| C | -7.64609400 | -3.59168800 | -2.32893200 |
| C | -9.10314000 | -3.55989700 | -2.49452800 |
| C | -9.77298600 | -4.51828800 | -3.26724200 |
| C | -11.88443700 | -3.47954800 | -2.82879100 |
| C | -11.27119800 | -2.48790800 | -2.04973100 |
| C | -12.05298200 | -1.41693100 | -1.42478600 |
| C | -13.43257700 | -1.29410900 | -1.64104700 |
| C | -13.54862200 | 0.60356700 | -0.18484400 |

| | | | |
|---|---|---|---|
| C | -12.17205900 | 0.53237800 | 0.07094400 |
| C | -11.51717600 | 1.46044200 | 0.99766600 |
| C | -12.24439700 | 2.41883300 | 1.71863400 |
| C | -10.25045100 | 3.21390200 | 2.78354100 |
| C | -9.47336400 | 2.26629400 | 2.10404800 |
| C | -8.02568500 | 2.17508100 | 2.30835600 |
| C | -7.37533900 | 2.87632000 | 3.33545300 |
| C | -5.25030900 | 2.04896800 | 2.61911500 |
| C | -5.84332800 | 1.35309900 | 1.55503900 |
| C | 3.44185700 | 5.94846100 | 0.78023000 |
| C | 3.06640200 | 6.43888200 | 2.19969400 |
| C | 2.99203300 | 7.00407200 | -0.25730800 |
| C | 4.97563800 | 5.82593700 | 0.70266000 |
| C | -3.79805000 | 5.74590700 | -0.15802600 |
| C | -5.32337000 | 5.55029600 | -0.06251100 |
| C | -3.46038300 | 6.24305600 | -1.58469700 |
| C | -3.38631300 | 6.82979900 | 0.86590700 |
| C | 5.37041400 | -4.68965300 | 4.59963300 |
| C | 6.06715500 | -4.29993400 | 5.92508100 |
| C | 5.57519400 | -6.20094400 | 4.33808900 |
| C | 3.86053600 | -4.42800100 | 4.75580800 |
| C | 4.48394500 | 4.06982600 | -4.24050300 |
| C | 4.74291000 | 3.50354600 | -5.65783900 |
| C | 4.89122800 | 5.56170700 | -4.20770500 |
| C | 2.97375200 | 3.97842000 | -3.95115600 |
| C | -5.13596000 | -7.11623000 | -2.96835000 |
| C | -3.15441400 | -5.61998200 | -3.27253200 |
| C | -4.57265000 | 2.36639700 | 5.57238200 |
| C | -4.24734700 | 4.47793800 | 4.22998000 |
| H | 4.29042900 | -4.42937700 | 0.62868500 |
| H | 2.70238700 | -6.24052800 | 0.70395100 |
| H | 0.01779400 | -7.01066200 | 0.87663400 |
| H | -2.29328200 | -6.18286000 | 0.90630100 |
| H | -2.87776700 | -3.82475000 | 0.72683400 |
| H | 0.78304200 | 5.49858400 | 0.57934400 |
| H | 4.51051500 | 3.41554700 | 0.36934400 |
| H | -4.73125100 | 3.16007000 | 0.22428100 |
| H | -1.11667300 | 5.41502600 | 0.05742800 |
| H | 4.17684500 | -2.92689600 | 2.80598800 |
| H | 7.95398400 | -4.64762800 | 3.78817900 |
| H | 9.74378600 | -5.06308200 | 3.16924200 |
| H | 13.27153000 | -3.51828400 | 1.38110900 |
| H | 14.13227000 | -2.17721000 | 0.34086900 |
| H | 13.89950200 | 1.27330700 | -2.11608300 |
| H | 12.86693900 | 2.65979000 | -2.91191800 |
| H | 9.07307900 | 4.34277700 | -3.80989700 |
| H | 7.18379400 | 3.95015000 | -4.03803100 |
| H | 3.67848200 | 2.40096200 | -2.16967000 |
| H | 3.38409700 | 5.71702600 | 2.95921400 |
| H | 3.55674300 | 7.39522200 | 2.41323000 |
| H | 1.98719500 | 6.58480400 | 2.30588200 |
| H | 1.91617500 | 7.19629000 | -0.21223500 |
| H | 3.23245000 | 6.68260400 | -1.27590300 |

| | | | |
|---|---|---|---|
| H | 3.50406000 | 7.95460300 | -0.07220400 |
| H | 5.30846000 | 5.48083700 | -0.28160200 |
| H | 5.42923600 | 6.80558500 | 0.88280900 |
| H | 5.37058100 | 5.13891500 | 1.45783500 |
| H | -5.62659800 | 5.18046100 | 0.92222400 |
| H | -5.82603000 | 6.50832400 | -0.22849600 |
| H | -5.69463700 | 4.85124300 | -0.81863000 |
| H | -2.38980700 | 6.43698100 | -1.70230000 |
| H | -3.99574500 | 7.17470500 | -1.79977200 |
| H | -3.75221200 | 5.50226800 | -2.33635200 |
| H | -3.94478500 | 7.75345800 | 0.67899400 |
| H | -3.59824600 | 6.50635400 | 1.88963900 |
| H | -2.32157800 | 7.07281800 | 0.80462300 |
| H | 5.93756700 | -3.23344800 | 6.13645600 |
| H | 5.63658600 | -4.86581500 | 6.75850100 |
| H | 7.14059400 | -4.50966200 | 5.90115100 |
| H | 5.08231800 | -6.50741900 | 3.40957900 |
| H | 5.14826900 | -6.78804600 | 5.15863100 |
| H | 6.63474100 | -6.46202100 | 4.25862300 |
| H | 3.65037500 | -3.37525100 | 4.97136300 |
| H | 3.30180400 | -4.71328400 | 3.85898800 |
| H | 3.47205800 | -5.01927900 | 5.59097000 |
| H | 4.45208900 | 2.44953500 | -5.71647900 |
| H | 4.15985300 | 4.05878000 | -6.40102200 |
| H | 5.79835300 | 3.57650900 | -5.93723600 |
| H | 4.72502600 | 5.99389500 | -3.21558400 |
| H | 4.29410800 | 6.12956800 | -4.92935200 |
| H | 5.94432200 | 5.70816600 | -4.46471900 |
| H | 2.72636200 | 4.35897800 | -2.95489500 |
| H | 2.42420700 | 4.57840300 | -4.68329700 |
| H | 2.60417000 | 2.95080300 | -4.02624900 |
| H | -4.96004000 | -7.19305100 | -1.89034000 |
| H | -6.20169100 | -7.28676800 | -3.14730000 |
| H | -4.58250700 | -7.92299100 | -3.46129800 |
| H | -2.63808800 | -6.43607200 | -3.78767600 |
| H | -2.89560200 | -5.68512300 | -2.21067000 |
| H | -2.75476500 | -4.67897000 | -3.66452200 |
| H | -4.06703300 | 2.82112200 | 6.43134300 |
| H | -3.81815400 | 1.83343100 | 4.98669800 |
| H | -5.28789000 | 1.62864900 | 5.95066400 |
| H | -3.50287400 | 4.01464700 | 3.57627500 |
| H | -4.73442900 | 5.28187400 | 3.66817300 |
| H | -3.71650000 | 4.92993400 | 5.07496100 |
| C | -11.84970000 | -5.57354700 | -4.24189300 |
| H | -11.22784200 | -5.91156800 | -5.07613100 |
| H | -12.06555900 | -6.45201300 | -3.62136900 |
| H | -12.80122600 | -5.21789900 | -4.64670400 |
| C | -15.66615000 | -0.15643000 | -1.33414200 |
| H | -16.19804700 | 0.31113500 | -0.50076900 |
| H | -15.83711600 | 0.46602300 | -2.22112100 |
| H | -16.12354200 | -1.13084600 | -1.52860600 |
| C | -12.44625500 | 4.31295300 | 3.37733300 |
| H | -11.85845000 | 5.20564100 | 3.60964800 |

| | | | |
|---|---|---|---|
| H | -12.79309600 | 3.89445500 | 4.33022900 |
| H | -13.33265400 | 4.62316100 | 2.81669700 |
| C | 15.65743100 | -0.50815600 | -1.12005400 |
| H | 16.06542300 | 0.48608800 | -1.32260700 |
| H | 16.16930300 | -0.91611900 | -0.24365400 |
| H | 15.91480700 | -1.14718000 | -1.97373900 |
| C | 12.39253800 | -5.52299800 | 2.95175700 |
| H | 12.00580100 | -5.79263700 | 3.93898800 |
| H | 12.35318200 | -6.42606000 | 2.33023800 |
| H | 13.44502000 | -5.24678300 | 3.06026000 |
| C | 11.71542200 | 4.71428100 | -4.21797400 |
| H | 11.09670300 | 5.04559600 | -5.05703800 |
| H | 11.90045100 | 5.59052600 | -3.58444300 |
| H | 12.68162800 | 4.38801800 | -4.61293700 |
| H | -2.93947200 | -2.35932300 | -0.80576800 |
| H | -3.78358800 | -3.62440000 | -1.96380900 |
| H | -7.34353200 | -5.35121800 | -3.52030700 |
| H | -9.21637500 | -5.30884400 | -3.75584900 |
| H | -12.95884700 | -3.47431700 | -2.96721400 |
| H | -4.17684600 | 1.97999500 | 2.73945200 |
| H | -7.96963800 | 3.46155700 | 4.02230700 |
| H | -9.77686000 | 3.91834700 | 3.45768700 |
| H | -13.31873700 | 2.49014300 | 1.59746100 |
| H | -14.14843000 | 1.37499400 | 0.28301300 |
| H | -13.94313800 | -1.98549700 | -2.30058000 |
| C | -5.28969500 | 3.45542200 | 4.73871300 |
| C | -6.27400900 | 4.19479800 | 5.66520600 |
| H | -6.79685700 | 5.00460600 | 5.14564800 |
| H | -7.02354300 | 3.51831700 | 6.08887300 |
| H | -5.72429800 | 4.64138400 | 6.49968000 |
| C | -4.67172800 | -5.74256500 | -3.50897400 |
| C | -4.92628800 | -5.67994000 | -5.03400000 |
| H | -4.36953100 | -6.47461100 | -5.54264500 |
| H | -5.98494500 | -5.80721200 | -5.27836300 |
| H | -4.59966800 | -4.71945500 | -5.44601400 |

## 18. References


S1 G. R. Fulmer, A. J. M. Miller, N. H. Sherden, H. E. Gottlieb, A. Nudelman, B. M. Stoltz, J. E. Bercaw and K. I. Goldberg *Organometallics* **2010**, *29*, 2176-2179.

S2 S. Reguardati, J. Pahapill, A. Mikhailov, Y. Stepanenko, A. Rebane *Opt. Express* **2016**, *24*, 4915-4918.

S3 C. Xu, W. W. Webb J. Opt. Soc. Am. B. **1996**, *13*, 481-491.

S4 G. M. Sheldrick *Acta Crystallogr*. **2007**, *64*, 112-122

S5 G. M. Sheldrick *Acta Crystallogr*. **2015**, *71*, 3-8.

S6 C. F. Macrae, P. R. Edgington, P. McCabe, E. Pidcock, G. P. Shields, R. Taylor, M. Towler and J. Van De Streek *J. Appl. Crystallogr*. **2006**, *39*, 453-457.

S7 M. Strohalm, D. Kavan, P. Novák, M. Volný and V. Havlíček *Anal. Chem.* **2010**, *11*, 4648-4651.

S8 M. J. Frisch, G. W. Trucks, H. B. Schlegel, G. E. Scuseria, M. A. Robb, J. R. Cheeseman, G. Scalmani, V. Barone, G. A. Petersson, H. Nakatsuji, X. Li, M. Caricato, A. V. Marenich, J. Bloino, B. G. Janesko, R. Gomperts, B. Mennucci, H. P. Hratchian, J. V. Ortiz, A. F. Izmaylov, J. L. Sonnenberg, D. Williams-Young, F. Ding, F. Lipparini, F. Egidi, J. Goings, B. Peng, A. Petrone, T. Henderson, D. Ranasinghe, V. G. Zakrzewski, J. Gao, N. Rega, G. Zheng, W. Liang, M. Hada, M. Ehara, K. Toyota, R. Fukuda, J. Hasegawa, M. Ishida, T. Nakajima, Y. Honda, O. Kitao, H. Nakai, T. Vreven, K. Throssell, J. A. Montgomery, Jr., J. E. Peralta, F. Ogliaro, M. J. Bearpark, J. J. Heyd, E. N. Brothers, K. N. Kudin, V. N. Staroverov, T. A. Keith, R. Kobayashi, J. Normand, K. Raghavachari, A. P. Rendell, J. C. Burant, S. S. Iyengar, J. Tomasi, M. Cossi, J. M. Millam, M. Klene, C. Adamo, R. Cammi, J. W. Ochterski, R. L. Martin, K. Morokuma, O. Farkas, J. B. Foresman, and D. J. Fox, Gaussian, Inc., Wallingford CT, 2016.

S9 a) A.D. Becke *J. Chem. Phys.* **1993**, *98*, 5648-5652; b) C. Lee, W. Yang, R.G. Parr *Phys. Rev. B* **1988**, *37*, 785-789.

S10 S. Miertus, E. Scrocco, J. Tomasi *Chem. Phys.* **1981**, *55*, 117-129.

S11 P. v R. Schleyer, C. Maerker, A. Dransfeld, H. Jiao, N. J. R. van Eikema Hommes *J. Am. Chem. Soc.* **1996**, *118*, 6317-6318.

S12 D. Geuenich, K. Hess, F. Köhler and R. Herges *Chem. Rev.* **2005**, *105*, 3758-3772.

S13 H. E. Zaugg, R. T. Rapala, M. T. Leffler, *J. Am. Chem. Soc.* **1948**, *70*, 3224.

S14 F. Xiao, X. Liu, K. Lin, Y. Zhou, W. Gao, Y. Lei, M. Liu, X. Huang, H. Wu, *J. Phys. Chem. C* . **2021**, *125*, 16792.

S15 B. F. Plummer, L. K. Steffen, T. L. Braley, W. G. Reese, K. Zych, G. van Dyke, B. Tulley, *J. Am. Chem. Soc.* **1993**, *115*, 11542.

S16 N. Venkatramaiah, S. Kumar, S. Patil, *Chem. Commun*. **2012**, *48*, 5007.
